# Supplementary material for: Scalable Parameter Estimation for Genome-Scale Biochemical Reaction Networks
Source: PLoS Comput Biol. 2017 Jan 23;13(1):e1005331. doi: 10.1371/journal.pcbi.1005331 (PMC5256869; doi:10.1371/journal.pcbi.1005331)
Supplement: S1 Supporting Information — This document provides a detailed derivation of forward and adjoint sensitivity analysis and one additional numerical example for the comparision to the MATLAB toolbox odeSD. (PDF) [file pcbi.1005331.s001.pdf]

Supplement to
Scalable parameter estimation for genome-scale biochemical reaction networks

Fabian Fröhlich [1,2], Barbara Kaltenbacher [3], Fabian J. Theis [1,2] and Jan Hasenauer [1,2,]

[1]Helmholtz Zentrum München - German Research Center for Environmental Health,
Institute of Computational Biology, 85764 Neuherberg, Germany,
[2]Technische Universität München, Center for Mathematics,
Chair of Mathematical Modeling of Biological Systems, 85748 Garching, Germany,
[3]University of Klagenfurt, Institute of Mathematics,
A-9020 Klagenfurt, Austria

Contents

1 Derivation of Forward and Adjoint Sensitivity Analysis for Gradient Evaluation **1**

1.1 Objective function 1

1.2 First and second order forward sensitivity analysis 2

1.3 Adjoint sensitivity analysis 2

2 Accuracy of the Numerically Computed Gradient **3**

3 Scaling of Forward and Adjoint Sensitivity Analysis with respect to the Number of Time Points **4**

4 Eigenvalue Spectrum of the Fisher Information Matrix for the model of ErbB signaling. **5**

5 Comparison of AMICI and odeSD **5**

1 Derivation of Forward and Adjoint Sensitivity Analysis for Gradient Evaluation

AMICI supports first and second order forward sensitivities and first order adjoint sensitivities. In the following we will provide all necessary equations for sensitivity and gradient calculation.

1.1 Objective function

We consider the objective function

$$J(\theta) = \frac{1}{2}\sum_{j=1}^{N}\sum_{i=1}^{n_y} \log(2\pi\sigma_{ij}^2(\theta)) + \left(\frac{\bar{y}_{ij} - h_i(x(t_j),\theta)}{\sigma_{ij}(\theta)}\right)^2, \quad (1)$$

with parameter dependent noise variance $\sigma_{ij}^2(\theta)$. For simplicity of notation we assume that $t_0 \leq t_1 < ... < t_N$.

The derivative of the objective function with respect to parameter θ_k, $i = 1, ..., n_\theta$, is given by

$$\frac{\partial J}{\partial \theta_k} = \sum_{j=1}^{N} \sum_{i=1}^{n_y} \frac{1}{\sigma_{ij}(\theta)} \left(\frac{1}{2} - \frac{(\bar{y}_{ij} - h_i(x(t_j), \theta))^2}{\sigma_{ij}^2(\theta)} \right) \left. \frac{\partial \sigma_{ij}}{\partial \theta_k} \right|_{(\theta)} - \frac{(\bar{y}_{ij} - h_i(x(t_j), \theta))}{\sigma_{ij}^2(\theta)} s_{i,k}^y(t_j) \tag{2}$$

in which $s_{i,k}^y(t_j)$ denotes the sensitivity of the output $y_i = h_i(x, \theta)$ at time t_j with respect to parameter θ_k. The sensitivity of the output can be expressed in terms of the sensitivity of the state

$$s_{i,k}^y(t_j) = \left. \frac{\partial h_i}{\partial x} \right|_{(x(t_j),\theta)}^T s_k^x(t_j) + \left. \frac{\partial h_i}{\partial \theta_k} \right|_{(x(t_j),\theta)}. \tag{3}$$

1.2 First and second order forward sensitivity analysis

The governing equations for first order forward sensitivities $s_k^x(t) : [t_0, t_N] \mapsto \mathbb{R}^{n_x \times n_\theta}$ are

$$\dot{s}_k^x(t) = \left. \frac{\partial f_i}{\partial x} \right|_{(x(t),\theta)} s_k^x(t) + \left. \frac{\partial f_i}{\partial \theta_k} \right|_{(x(t),\theta)}, \qquad \text{with } s_k^x(t_0) = \left. \frac{\partial x_0}{\partial \theta_k} \right|_{(\theta)}. \tag{4}$$

Second order equations can be obtained by differentiating both sides of (4) with respect to the parameter θ_l:

$$\dot{s}_{kl}^{(2),x}(t) = \left. \frac{\partial f}{\partial x} \right|_{(x(t),\theta)} s_{kl}^{(2),x}(t) + s_l^x(t)^T \left. \frac{\partial^2 f}{\partial x^2} \right|_{(x(t),\theta)} s_k^x(t) + \left. \frac{\partial^2 f}{\partial \theta_k \partial \theta_l} \right|_{(x(t),\theta)}, \qquad \text{with } s_{kl}^{(2),x}(t_0) = \left. \frac{\partial^2 x_0}{\partial \theta_k \partial \theta_l} \right|_{(\theta)},$$

where $s_{kl}^{(2),x}(t) : [t_0, t_N] \mapsto \mathbb{R}^{n_x \times n_\theta \times n_\theta}$ denotes the second order sensitivity of states x with respect to parameters θ_k and θ_l. Note that for two times continuously differentiable functions f the second order sensitivities are, according to Schwarz' Theorem, symmetric in k and l.

1.3 Adjoint sensitivity analysis

The evaluation of every gradient entry $\partial J / \partial \theta_k$ using (2) requires the solution of (4) which can be prohibitively computationally demanding for complex systems with a large number of parameters. In the following we will derive an evaluation scheme of this gradient of objective functions of the form (1) which does depend on the state sensitivities $s_k^x(t)$ and thus does not require a solution to (4). This will be achieved by reformulating the term which contains the sensitivity of the state with respect to the parameters,

$$\sum_{j=1}^{N} \sum_{i=1}^{n_y} \left. \frac{\partial h_i}{\partial x} \right|_{(x(t_j),\theta)}^T \frac{(\bar{y}_{ij} - h_i(x(t_j), \theta))}{\sigma_{ij}^2(\theta)} s_k^x(t_j). \tag{5}$$

We introduce the adjoint state $p(t) : [t_0, t_N] \mapsto \mathbb{R}^{n_x}$ as the solution to the backward differential equation

$$\begin{aligned}
&\lim_{t \to t_j^+} p(t) = 0 \\
&\text{for } j = N : -1 : 1 \\
&\quad \dot{p}(t) = - \left. \frac{\partial f}{\partial x} \right|_{(x(t),\theta)}^T p(t), \quad t \in (t_{j-1}, t_j) \\
&\quad \text{with } p(t_j) = \lim_{t \to t_j^+} p(t) + \sum_{i=1}^{n_y} \left. \frac{\partial h_i}{\partial x} \right|_{(x(t),\theta)}^T \frac{(\bar{y}_{ij} - h_i(x(t_j), \theta))}{\sigma_{ij}^2(\theta)}
\end{aligned} \tag{6}$$

which is defined on intervals (t_{j-1}, t_j), $j = 1, \ldots, N$. The dimension of the adjoint state $p(t)$ equals the number of state equations, which is usually the same as the number of state variables $x(t)$. From (6) it follows that for each of the individual subintervals with $j \in \{0, \ldots, N-1\}$ it holds that:

$$0 = \int_{t_j}^{t_{j+1}} \left(\dot{p}(t) + \left. \frac{\partial f}{\partial x} \right|_{(x(t),\theta)}^T p(t) \right)^T s_k^x(t) dt, \tag{7}$$

By integration by parts of (7) we obtain

$$0 = \int_{t_j}^{t_{j+1}} p(t)^T \left(-\dot{s}_k^x(t) + \left. \frac{\partial f}{\partial x} \right|_{(x(t),\theta)}^T s_k^x(t) \right) dt + \lim_{t \to t_{j+1}^-} p(t)^T s_k^x(t) - \lim_{t \to t_j^+} p(t)^T s_k^x(t) \tag{8}$$

We proceed by simplifying the second term of the integral by using the forward sensitivity equation (4) and by evaluating the limit from above $\lim_{t\to t_j^+} p(t)$ using the definition of the end value on the intervals, see the last line of (6). This yields

$$
\begin{aligned}
0 = &-\int_{t_j}^{t_{j+1}} p(t)^T \frac{\partial f}{\partial \theta_k}(x(t),\theta)dt + p(t_{j+1})^T s_k^x(t_{j+1}) - p(t_j)^T s_k^x(t_j) \\
&+ \sum_{i=1}^{n_y} \left.\frac{\partial h_i}{\partial x}\right|_{(x(t_j),\theta)}^T \frac{(\bar{y}_{ij} - h_i(x(t_j),\theta))}{\sigma_{ij}^2(\theta)} s_k^x(t_j).
\end{aligned}
\tag{9}
$$

Note that the latter only holds for $j > 0$ while for $j = 0$ (for which no measurement is available), the last summand is 0. Summing (9) over j from 0 to $N-1$ yields

$$
\begin{aligned}
0 = &-\sum_{j=0}^{N-1} \int_{t_j}^{t_{j+1}} p(t)^T \left.\frac{\partial f}{\partial \theta_k}\right|_{(x(t),\theta)} dt + \underbrace{\sum_{j=0}^{N-1} p(t_{j+1}) s_k^x(t_{j+1}) - \sum_{j=0}^{N-1} p(t_j) s_k^x(t_j)}_{(*)} \\
&+ \sum_{j=1}^{N-1} \sum_{i=1}^{n_y} \left.\frac{\partial h_i}{\partial x}\right|_{(x(t),\theta)}^T \frac{(\bar{y}_{ij} - h_i(x(t_j),\theta))}{\sigma_{ij}^2(\theta)} s_k^x(t_j)
\end{aligned}
$$

All terms in $(*)$ either cancel or evaluate to 0 except for $p(t_N)s_k^x(t_N)$ and $p(t_0)^T s_k^x(t_0)$. Additionally merging the sum over integrals into one integral and using the last line of (6) for expressing the term $p(t_N)s_k^x(t_N)$ yields

$$
0 = -\int_{t_0}^{t_N} p(t)^T \left.\frac{\partial f}{\partial \theta_k}\right|_{(x(t),\theta)} dt - p(t_0)^T s_k^x(t_0) + \sum_{j=1}^{N} \sum_{i=1}^{n_y} \left.\frac{\partial h_i}{\partial x}\right|_{(x(t),\theta)}^T \frac{(\bar{y}_{ij} - h_i(x(t_j),\theta))}{\sigma_{ij}^2(\theta)} s_k^x(t_j).
$$

By replacing $p(t_0)^T s_k^x(t_0)$ with the definition of the initial condition of the forward sensitivity equation in (4) we obtain an expression for (5) which is independent of the state sensitivities $s_k^x(t_0)$:

$$
\sum_{j=1}^{N} \sum_{i=1}^{n_y} \left.\frac{\partial h_i}{\partial x}\right|_{(x(t_j),\theta)}^T \frac{(\bar{y}_{ij} - h_i(x(t_j),\theta))}{\sigma_{ij}^2(\theta)} s_k^x(t_j) = \int_{t_0}^{t_N} p(t)^T \left.\frac{\partial f}{\partial \theta_k}\right|_{(x(t),\theta)} dt + p(t_0)^T \left.\frac{\partial x_0}{\partial \theta_k}\right|_{(\theta)}.
$$

This alternative formulation of the problematic term (5) is then used to formulate the gradient (2). We obtain the objective function gradient

$$
\begin{aligned}
\frac{\partial J}{\partial \theta_k} = &\sum_{j=1}^{N} \sum_{i=1}^{n_y} \frac{1}{\sigma_{ij}(\theta)} \left(\frac{1}{2} - \frac{(\bar{y}_{ij} - h_i(x(t_j),\theta))^2}{\sigma_{ij}^2(\theta)} \right) \left.\frac{\partial \sigma_{ij}}{\partial \theta_k}\right|_{(\theta)} - \frac{(\bar{y}_{ij} - h_i(x(t_j),\theta))}{\sigma_{ij}^2(\theta)} \left.\frac{\partial h_i}{\partial \theta_k}\right|_{(x(t_j),\theta)} \\
&- \int_{t_0}^{t_N} p(t)^T \left.\frac{\partial f}{\partial \theta_k}\right|_{(x(t),\theta)} dt - p(t_0)^T \left.\frac{\partial x_0}{\partial \theta_k}\right|_{(\theta)},
\end{aligned}
\tag{10}
$$

with $k = 1, ..., n_\theta$. This expression no longer depends on the state sensitivities $s_k^x(t)$ but instead on the adjoint state $p(t)$. In contrast to the state sensitivities, the adjoint state does not depend on parameters und thus only needs to be evaluated once. This reduces the computational complexity of one gradient evaluation from $n_p + 1$ systems of the size n_x to 2 systems of the size n_x. The computational complexity of the numerical integration required to evaluate (10) is usually negligible.

2 Accuracy of the Numerically Computed Gradient

We compared the numerical gradient approximations obtained by finite differences, forward sensitivity analysis and adjoint sensitivity analysis. To evaluate the accuracy of the individual appraoches we investigated

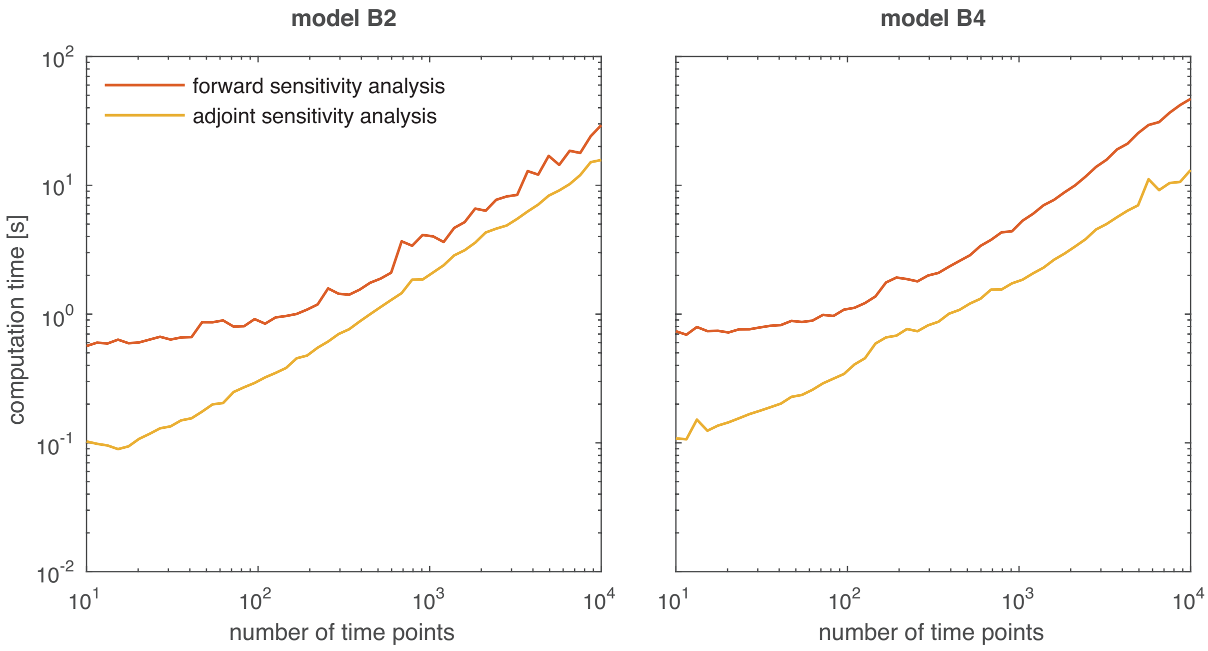

Supplement Figure 1: Dependence of the computation time on the number of time points for the B2 and B4 model. Data used for the adjoint approach was generated by simulating the system at nominal parameter and adding artificial noise.

the maximum relative difference

$$\Delta_{i,rel} = \begin{cases} 0 & \text{if } \frac{\partial J}{\partial \theta_i}^{\text{adjoint}'} = 0,\ \frac{\partial J}{\partial \theta_i}^{\text{method}} = 0 \\ \left| \frac{\frac{\partial J}{\partial \theta_i}^{\text{method}} - \frac{\partial J}{\partial \theta_i}^{\text{adjoint}'}}{\frac{\partial J}{\partial \theta_i}^{\text{adjoint}'}} \right| & \text{if } \frac{\partial J}{\partial \theta_i}^{\text{adjoint}'} \neq 0,\ \frac{\partial J}{\partial \theta_i}^{\text{method}} = 0 \\ \left| \frac{\frac{\partial J}{\partial \theta_i}^{\text{method}} - \frac{\partial J}{\partial \theta_i}^{\text{adjoint}'}}{\frac{\partial J}{\partial \theta_i}^{\text{method}}} \right| & \text{if } \frac{\partial J}{\partial \theta_i}^{\text{adjoint}'} = 0,\ \frac{\partial J}{\partial \theta_i}^{\text{method}} \neq 0 \\ \max\left(\left| \frac{\frac{\partial J}{\partial \theta_i}^{\text{method}} - \frac{\partial J}{\partial \theta_i}^{\text{adjoint}'}}{\frac{\partial J}{\partial \theta_i}^{\text{adjoint}'}} \right|, \left| \frac{\frac{\partial J}{\partial \theta_i}^{\text{method}} - \frac{\partial J}{\partial \theta_i}^{\text{adjoint}'}}{\frac{\partial J}{\partial \theta_i}^{\text{method}}} \right| \right) & \text{otherwise} \end{cases},$$

where method $\in$ {adjoint', forward, finite difference} indicates the employed gradient approximation scheme. For method $\in$ {adjoint, forward, finite difference} we used default tolerances default accuracies (absolute error $< 10^{-16}$, relative error $< 10^{-8}$)) and for method=adjoint' we used high accuracies (absolute error $< 10^{-32}$, relative error $< 10^{-16}$). For finite differences we used a step size of 10^{-3}. The maximum over both relative error variants ensures symmetry with respect to the choice of methods and accounts for the fact that we do not know the ground truth.

3 Scaling of Forward and Adjoint Sensitivity Analysis with respect to the Number of Time Points

We compared the scaling of adjoint and forward sensitivity analysis with respect to the number of time points at which data is available. We found that for both appraoches the computation time increases linearly with the number of time points (see Figure 2). An additional analysis suggests that for forward sensitivity analysis this is caused by the need to evaluate the sensitivities at more time points than required by the employed multi-step solver. This appears to a slight shortcoming of the the multi-step solver in CVODES [1], which requires the evaluation of a polynomial equation for each sensitivity. For adjoint sensitivity analysis the increase in computation time is caused by the reinitialization of adjoint sensitivities at the discontinuities at the data time points. This is also a shortcoming of using a multi-step solver as the order of the solver and the time-step needs are reset to small values at every discontinuity. For both, forward and adjoint sensitivity analysis, the adverse scaling with the number of time points could be eliminated by switching to a single-step solver. We are, however, not aware of any publicly available general purpose differential equation solvers with

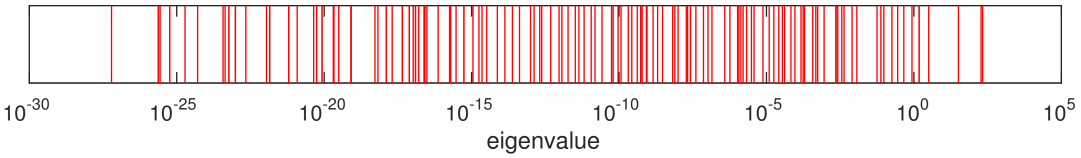

Supplement Figure 2: Eigenvalue spectrum of the Fisher Information Matrix at the nominal parameter value for the ErbB signalling model.

similar capabilities, such as rootfinding and sensitivity support, as CVODES.

4 Eigenvalue Spectrum of the Fisher Information Matrix for the model of ErbB signaling.

A key challenge for large-scale mathematical modeling is data availability. Insufficient data can easily result in structural and practical identifiability problems. To assess the identifiability of the model for ErbB signalling we investigated the eigenvalue spectrum of the Fisher Information Matrix at the nominal parameter value. We found that the nonzero eigenvalues span almost 30 orders of magnitude and that there are 73 eigenvalues equal to zero. This suggests that most parameters in the model are poorly or not at all identifiable from the considered experimental data.

5 Comparison of AMICI and odeSD

We compared AMICI and odeSD [2], a specialized ODE integrator for the efficient computation of forward sensitivities. For our evaluation we used the newest publicly available version of odeSD (odeSD v1.1) and considered the Kholodenko model [3]. This model has been studied by [2]. To avoid spurious effects from thread starvation, MATLAB was started with the `-singeCompThread` flag to ensure single-threading. This was necessary as odeSD uses multi-threaded routines from the MATLAB Math Kernel Library, which resulted in generally slower computation times during high computational load on the testing machine.

For the Kholodenko model with ten equidistantly spaced time points between $t = 0$ and $t = 50$, odeSD with mex right hand side required 14 seconds for 100 repeated integrations while AMICI required 17 seconds for 100 repeated integrations for the calculation of the solution. Accordingly, the computation time is comparable while AMICI offers additional options, including adjoint sensitivity analysis. If the number of intermediate time points increases, the computation time for odeSD remains roughly the same. For AMICI the overall computation time increases slightly as the time requirement for evaluating the (differentiated) interpolating polynomial becomes increasingly important for the total computation time.

References

[1] Serban R, Hindmarsh AC. CVODES: An ODE solver with sensitivity analysis capabilities. ACM T Math Software; 2005;31(3):363–396.

[2] Gonnet P, Dimopoulos S, Widmer L, Stelling J. A specialized ODE integrator for the efficient computation of parameter sensitivities. BMC Syst Biol; 2012;6(46).

[3] Kholodenko BN, Demin OV, Moehren G, Hoek JB. Quantification of short term signaling by the epidermal growth factor receptor. J Biol Chem; 1999;274(42):30169–30181.
