# Supplementary material for: Scalable Parameter Estimation for Genome-Scale Biochemical Reaction Networks
Source: PLoS Comput Biol. 2017 Jan 23;13(1):e1005331. doi: 10.1371/journal.pcbi.1005331 (PMC5256869; doi:10.1371/journal.pcbi.1005331)
Supplement: S1 Code — This zip-file contains the MATLAB code for the simulation and application examples presented in the paper. We provide implementations of all models, parameter estimation to allow everybody to reproduce the results. (ZIP) [file pcbi.1005331.s002.zip › code/AMICI/AMICI_guide.pdf]

AMICI

Generated by Doxygen 1.8.10

Wed May 4 2016 14:08:03

Contents

1 AMICI 0.1 General Documentation **1**

1.1 Introduction 1

1.2 Availability 1

1.3 Installation 2

2 Model Definition & Simulation **3**

2.1 Model Definition 3

2.2 Model Compilation 6

2.3 Model Simulation 6

3 Examples **8**

3.1 Example Events 9

3.2 Example Dirac 14

3.3 Example Steady State 18

3.4 Example JAK/STAT Adjoint 23

3.5 Example Dirac Adjoint 26

3.6 Example Dirac Second Order Forward 29

3.7 Example Dirac Directional Second Order Forward 32

3.8 Example Adjoint 35

4 Code Organization **39**

5 Hierarchical Index **39**

5.1 Class Hierarchy 40

6 Class Index **40**

6.1 Class List 40

7 Class Documentation **41**

7.1 amidata Class Reference 41

7.2 amievent Class Reference 44

7.3 amifun Class Reference 46

7.4 amimodel Class Reference 51

7.5 amioption Class Reference 65

7.6 ExpData Struct Reference 70

7.7 modelTest Class Reference 71

7.8 optsym Class Reference 71

7.9 ReturnData Struct Reference 72

7.10 SBMLode Class Reference 75

7.11 TempData Struct Reference 80

7.12 UserData Struct Reference 87

8 File Documentation 93

8.1 amiwrap.c File Reference . . . 93
8.2 amiwrap.m File Reference . . . 95
8.3 SBML2AMICI.m File Reference . . . 96
8.4 src/amici.c File Reference . . . 96
8.5 src/spline.c File Reference . . . 120
8.6 src/symbolic_functions.c File Reference . . . 126
8.7 symbolic/am_and.m File Reference . . . 145
8.8 symbolic/am_ge.m File Reference . . . 145
8.9 symbolic/am_gt.m File Reference . . . 146
8.10 symbolic/am_if.m File Reference . . . 146
8.11 symbolic/am_le.m File Reference . . . 146
8.12 symbolic/am_lt.m File Reference . . . 146
8.13 symbolic/am_max.m File Reference . . . 146
8.14 symbolic/am_min.m File Reference . . . 147
8.15 symbolic/am_or.m File Reference . . . 147
8.16 symbolic/am_stepfun.m File Reference . . . 147

Index 149

1 AMICI 0.1 General Documentation

1.1 Introduction

AMICI is a MATLAB interface for the `SUNDIALS` solvers CVODES (for ordinary differential equations) and IDAS (for algebraic differential equations). AMICI allows the user to specify differential equation models in terms of symbolic variables in MATLAB and automatically compiles such models as .mex simulation files. In contrast to the SUNDIALSTB interface, all necessary functions are transformed into native C code, which allows for a significantly faster numerical integration. Beyond forward integration, the compiled simulation file also allows for first and second order forward sensitivity analysis, steady state sensitivity analysis and adjoint sensitivity analysis for likelihood based output functions.

The interface was designed to provide routines for efficient gradient computation in parameter estimation of biochemical reaction models but is also applicable to a wider range of differential equation constrained optimization problems.

1.2 Availability

The sources for AMICI are accessible as

- Source `tarball`
- Source `zipball`
- GIT repository on `github`

Once you've obtained your copy check out the Installation

1.2.1 Obtaining AMICI via the GIT versioning system

In order to always stay up-to-date with the latest AMICI versions, simply pull it from our GIT repository and recompile it when a new release is available. For more information about GIT checkout their website

The GIT repository can currently be found at https://github.com/FFroehlich/AMICI and a direct clone is possible via

```
git clone https://github.com/FFroehlich/AMICI.git AMICI
```

1.2.2 License Conditions

This software is available under the BSD license

Copyright (c) 2015, Fabian Fröhlich and Jan Hasenauer All rights reserved.

Redistribution and use in source and binary forms, with or without modification, are permitted provided that the following conditions are met:

- Redistributions of source code must retain the above copyright notice, this list of conditions and the following disclaimer.
- Redistributions in binary form must reproduce the above copyright notice, this list of conditions and the following disclaimer in the documentation and/or other materials provided with the distribution.

THIS SOFTWARE IS PROVIDED BY THE COPYRIGHT HOLDERS AND CONTRIBUTORS "AS IS" AND ANY EXPRESS OR IMPLIED WARRANTIES, INCLUDING, BUT NOT LIMITED TO, THE IMPLIED WARRANTIES OF MERCHANTABILITY AND FITNESS FOR A PARTICULAR PURPOSE ARE DISCLAIMED. IN NO EVENT SHALL THE COPYRIGHT HOLDER OR CONTRIBUTORS BE LIABLE FOR ANY DIRECT, INDIRECT, INCIDENTAL, SPECIAL, EXEMPLARY, OR CONSEQUENTIAL DAMAGES (INCLUDING, BUT NOT LIMITED TO, PROCUREMENT OF SUBSTITUTE GOODS OR SERVICES; LOSS OF USE, DATA, OR PROFITS; OR BUSINESS INTERRUPTION) HOWEVER CAUSED AND ON ANY THEORY OF LIABILITY, WHETHER IN CONTRACT, STRICT LIABILITY, OR TORT (INCLUDING NEGLIGENCE OR OTHERWISE) ARISING IN ANY WAY OUT OF THE USE OF THIS SOFTWARE, EVEN IF ADVISED OF THE POSSIBILITY OF SUCH DAMAGE.

1.3 Installation

If AMICI was downloaded as a zip, it needs to be unpacked in a convenient directory. If AMICI was obtained via cloning of the git repository, no further unpacking is necessary.

To use AMICI, start MATLAB and add the AMICI direcory to the MATLAB path. To add all toolbox directories to the MATLAB path, execute the matlab script

```
installToolbox.m
```

To store the installation for further MATLAB session, the path can be saved via

```
savepath
```

For the compilation of .mex files, MATLAB needs to be configured with a working C compiler. The C compiler needs to be installed and configured via:

```
mex -setup c
```

For a list of supported compilers we refer to the mathworks documentation: mathworks.de

The tools SUNDIALS and SuiteSparse shipped with AMICI do **not** require further installation.

AMICI uses the following packages from SUNDIALS:

CVODES: the sensitivity-enabled ODE solver in SUNDIALS. Radu Serban and Alan C. Hindmarsh. *ASME 2005 International Design Engineering Technical Conferences and Computers and Information in Engineering Conference.* American Society of Mechanical Engineers, 2005. PDF

IDAS

AMICI uses the following packages from SuiteSparse:

Algorithm 907: KLU, A Direct Sparse Solver for Circuit Simulation Problems. Timothy A. Davis, Ekanathan Palamadai Natarajan, *ACM Transactions on Mathematical Software*, Vol 37, Issue 6, 2010, pp 36:1 - 36:17. PDF

Algorithm 837: AMD, an approximate minimum degree ordering algorithm, Patrick R. Amestoy, Timothy A. Davis, Iain S. Duff, *ACM Transactions on Mathematical Software*, Vol 30, Issue 3, 2004, pp 381 - 388. PDF

Algorithm 836: COLAMD, a column approximate minimum degree ordering algorithm, Timothy A. Davis, John R. Gilbert, Stefan I. Larimore, Esmond G. Ng *ACM Transactions on Mathematical Software*, Vol 30, Issue 3, 2004, pp 377 - 380. PDF

2 Model Definition & Simulation

In the following we will give a detailed overview how to specify models in AMIWRAP and how to call the generated simulation files.

2.1 Model Definition

This guide will guide the user on how to specify models in MATLAB. For example implementations see the examples in the example directory.

2.1.1 Header

The model definition needs to be defined as a function which returns a struct with all symbolic definitions and options.

```
function [model] = example_model_syms()
```

2.1.2 Options

Set the options by specifying the respective field of the modelstruct

```
model.(fieldname) = (value)
```

The options specify default options for simulation, parametrisation and compilation. All of these options are optional.

field	description	default
.param	parametrisation 'log'/'log10'/'lin'	'lin'
.debug	flag to compile with debug symbols	false
.forward	flag to activate forward sensitivities	true
.adjoint	flag to activate adjoint sensitivities	true

When set to true, the fields 'noforward' and 'noadjoint' will speed up the time required to compile the model but also disable the respective sensitivity computation.

2.1.3 States

Create the respective symbolic variables. The name of the symbolic variable can be chosen arbitrarily.

```
syms state1 state2 state3
```

Create the state vector containing all states:

```
x = [ state1 state2 state3 ];
```

2.1.4 Parameters

Create the respective symbolic variables. The name of the symbolic variable can be chosen arbitrarily. Sensitivities **will be derived** for all paramaters.

```
syms param1 param2 param3 param4 param5 param6
```

Create the parameters vector

```
p = [ param1 param2 param3 param4 param5 param6 ];
```

2.1.5 Constants

Create the respective symbolic variables. The name of the symbolic variable can be chosen arbitrarily. Sensitivities with respect to constants **will not be derived**.

```
syms const1 const2
```

Create the parameters vector

```
k = [ const1 const2 ];
```

2.1.6 Differential Equation

For time-dependent differential equations you can specify a symbolic variable for time. This **needs** to be denoted by t.

```
syms t
```

Specify the right hand side of the differential equation f or xdot

```
xdot(1) = [ const1 - param1*state1 ];
xdot(2) = [ +param2*state1 + dirac(t-param3) - const2*state2 ];
xdot(3) = [ param4*state2 ];
```

or

```
f(1) = [ const1 - param1*state1 ];
f(2) = [ +param2*state1 + dirac(t-param3) - const2*state2 ];
f(3) = [ param4*state2 ];
```

The specification of f or xdot may depend on States, Parameters and Constants.

For DAEs also specify the mass matrix.

```
M = [1, 0, 0;...
0, 1, 0;...
0, 0, 0];
```

The specification of M may depend on parameters and constants.

For ODEs the integrator will solve the equation $\dot{x} = f$ and for DAEs the equations $M \cdot \dot{x} = f$. AMICI will decide whether to use CVODES (for ODEs) or IDAS (for DAEs) based on whether the mass matrix is defined or not.

In the definition of the differential equation you can use certain symbolic functions. For a full list of available functions see symbolic_functions.c.

Dirac functions can be used to cause a jump in the respective states at the specified time-point. This is typically used to model injections, or other external stimuli. Spline functions can be used to model time/state dependent response with unkown time/state dependence.

2.1.7 Initial Conditions

Specify the initial conditions. These may depend on Parameters on Constants and must have the same size as x.

```
x0 = [ param4, 0, 0 ];
```

2.1.8 Observables

Specify the observables. These may depend on Parameters and Constants.

```
y(1) = state1 + state2;
y(2) = state3 - state2;
```

In the definition of the observable you can use certain symbolic functions. For a full list of available functions see symbolic_functions.c. Dirac functions in observables will have no effect.

2.1.9 Events

Specifying events is optional. Events are specified in terms of a trigger function, a bolus fuction and an output function. The roots of the trigger function defines the occurences of the event. The bolus function defines the change in the state on event occurences. The output function defines the expression which is evaluated and reported by the simulation routine on every event occurence. The user can create events by constructing a vector of objects of the class amievent.

```
event(1) = amievent(state1 - state2,0,[]);
```

Events may depend on States, Parameters and Constants but **not** on Observables

2.1.10 Standard Deviation

Specifying of standard deviations is optional. It only has an effect when computing adjoint sensitivities. It allows the user to specify standard deviations of experimental data for Observables and Events.

Standard deviaton for observable data is denoted by sigma_y

```
sigma_y(1) = param5;
```

Standard deviaton for event data is denoted by sigma_y

```
sigma_t(1) = param6;
```

Both sigma_y and sigma_t can either be a scalar or of the same dimension as the Observables / Events function. They can depend on time and Parameters but must not depend on the States or Observables. The values provided in sigma_y and sigma_t will only be used if the value in Sigma_Y or Sigma_T in the user-provided data struct is NaN. See Model Simulation for details.

2.1.11 Attach to Model Struct

Eventually all symbolic expressions need to be attached to the model struct.

```
model.sym.x = x;
model.sym.k = k;
model.sym.event = event;
model.sym.xdot = xdot;
% or
model.sym.f = f;
model.sym.M = M; %only for DAEs
model.sym.p = p;
model.sym.x0 = x0;
model.sym.y = y;
model.sym.sigma_y = sigma_y;
model.sym.sigma_t = sigma_t;
```

2.2 Model Compilation

The model can then be compiled by calling amiwrap:

```
amiwrap(modelname,'example_model_syms',dir,o2flag)
```

Here modelname should be a string defining the modelname, dir should be a string containing the path to the directory in which simulation files should be placed and o2flag is a flag indicating whether second order sensitivities should also be compiled. The user should make sure that the previously defined function 'example_model_syms' is in the user path. Alternatively, the user can also call the function 'example_model_syms'

```
[model] = example_model_syms()
```

and subsequently provide the generated struct to amiwrap(), instead of providing the symbolic function:

```
amiwrap(modelname,model,dir,o2flag)
```

In a similar fashion, the user could also generate multiple model and pass them directly to amiwrap() without generating respective model definition scripts.

See also

amiwrap()

2.3 Model Simulation

After the call to amiwrap() two files will be placed in the specified directory. One is a am_*modelname*.mex and the other is simulate_*modelname*.m. The mex file should never be called directly. Instead the MATLAB script, which acts as a wrapper around the .mex simulation file should be used.

The simulate_*modelname*.m itself carries extensive documentation on how to call the function, what it returns and what additional options can be specified. In the following we will give a short overview of possible function calls.

2.3.1 Integration

Define a time vector:

```
t = linspace(0,10,100)
```

Generate a parameter vector:

```
theta = ones(6,1);
```

Generate a constants vector:

```
kappa = ones(2,1);
```

Integrate:

```
sol = simulate_modelname(t,theta,kappa,[],options)
```

The integration status will be indicated by the sol.status flag. Negative values indicated failed integration. The states will then be available as sol.x. The observables will then be available as sol.y. The events will then be available as sol.root. If no event occured there will be an event at the end of the considered interval with the final value of the root function stored in sol.rval.

Alternatively the integration call also be called via

```
[status,t,x,y] = simulate_modelname(t,theta,kappa,[],options)
```

The integration status will be indicated by the status flag. Negative values indicated failed integration. The states will then be available as x. The observables will then be available as y. No event output will be given.

2.3.2 Forward Sensitivities

Define a time vector:

```
t = linspace(0,10,100)
```

Generate a parameter vector:

```
theta = ones(6,1);
```

Generate a constants vector:

```
kappa = ones(2,1);
```

Set the sensitivity computation to forward sensitivities and Integrate:

```
options.sensi = 1;
options.forward = true;
sol = simulate_modelname(t,theta,kappa,[],options)
```

The integration status will be indicated by the sol.status flag. Negative values indicated failed integration. The states will then be available as sol.x, with the derivative with respect to the parameters in sol.sx. The observables will then be available as sol.y, with the derivative with respect to the parameters in sol.sy. The events will then be available as sol.root, with the derivative with respect to the parameters in sol.sroot. If no event occured there will be an event at the end of the considered interval with the final value of the root function stored in sol.rootval, with the derivative with respect to the parameters in sol.srootval

Alternatively the integration call also be called via

```
[status,t,x,y,sx,sy] = simulate_modelname(t,theta,kappa,[],options)
```

The integration status will be indicated by the status flag. Negative values indicated failed integration. The states will then be available as x, with derivative with respect to the parameters in sx. The observables will then be available as y, with derivative with respect to the parameters in sy. No event output will be given.

2.3.3 Adjoint Sensitivities

Define a time vector:

```
t = linspace(0,10,100)
```

Generate a parameter vector:

```
theta = ones(6,1);
```

Set the sensitivity computation to adjoint sensitivities:

```
options.sensi = 1;
options.adjoint = true;
```

Define Experimental Data:

```
D.Y = [NaN(1,2)],ones(length(t)-1,2)];
D.Sigma_Y = [0.1*ones(length(t)-1,2),NaN(1,2)];
D.T = ones(1,1);
D.Sigma_T = NaN;
```

The NaN values in Sigma_Y and Sigma_T will be replaced by the specification in Standard Deviation. Data points with NaN value will be completely ignored.

Generate a constants vector:

```
kappa = ones(2,1);
```

Integrate:

```
sol = simulate_modelname(t,theta,kappa,D,options)
```

The integration status will be indicated by the sol.status flag. Negative values indicated failed integration. The log-likelihood will then be available as sol.llh and the derivative with respect to the parameters in sol.sllh. Notice that for adjoint sensitivities no state, observable and event sensitivities will be available. Yet this approach can be expected to be significantly faster for systems with a large number of parameters.

2.3.4 Steady State Sensitivities

This will compute state sensitivities according to the formula $s_k^x = -\left(\frac{\partial f}{\partial x}\right)^{-1}\frac{\partial f}{\partial \theta_k}$

In the current implementation this formulation does not allow for conservation laws as this would result in a singular Jacobian.

Define a final timepoint t:

```
t = 100
```

Generate a parameter vector:

```
theta = ones(6,1);
```

Generate a constants vector:

```
kappa = ones(2,1);
```

Set the sensitivity computation to steady state sensitivities:

```
options.sensi = 1;
options.ss = 1;
```

Integrate:

```
sol = simulate_modelname(t,theta,kappa,D,options)
```

The states will then be available as sol.x, with the derivative with respect to the parameters in sol.sx. The observables will then be available as sol.y, with the derivative with respect to the parameters in sol.sy. Notice that for steady state sensitivities no event sensitivities will be available. For the accuracy of the computed derivatives it is essential that the system is sufficiently close to a steady state. This can be checked by examining the right hand side of the system at the final time-point via sol.xdot.

3 Examples

In this section we include multiple examples on defining and simulating models.

Example Events : Forward Sensitivities for model with events and discontinuities.

Example Dirac : Forward Sensitivities for mRNA transfection model with bolus injection.

Example Steady State : Steady State Sensitivities.

Example JAK/STAT Adjoint : Adjoint Sensitivities for JAK/STAT model with parametric standard deviation.

Example Dirac Adjoint : Adjoint Sensitivities for mRNA transfection model with bolus injection.

Example Dirac Second Order Forward : Second order forward sensitivities for mRNA transfection model with bolus injection.

Example Dirac Directional Second Order Forward : Directional second order forward sensitivities for mRNA transfection model with bolus injection.

Example Adjoint : Adjoint Sensitivities for simple model with analytic solution.

3.1 Example Events

3.1.1 Model Definition

```
function [model] = model_events_syms()


% set the parametrisation of the problem options are 'log', 'log10' and
% 'lin' (default).
model.param = 'log10';
```

STATES

```
% create state syms
syms x1 x2 x3

% create state vector
model.sym.x = [
x1 x2 x3
];
```

PARAMETERS (for these sensitivities will be computed)

```
% create parameter syms
syms p1 p2 p3 p4

% create parameter vector
model.sym.p = [p1,p2,p3,p4];

% set the parametrisation of the problem options are 'log', 'log10' and
% 'lin' (default).
model.param = 'log10';
```

CONSTANTS (for these no sensitivities will be computed) this part is optional and can be ommited

```
% create parameter syms
syms k1 k2 k3 k4

% create parameter vector
model.sym.k = [k1 k2 k3 k4];
```

SYSTEM EQUATIONS

```
% create symbolic variable for time
syms t

model.sym.xdot = sym(zeros(size(model.sym.x)));

% piecewise defined function
model.sym.xdot(1) = -p1*heaviside(t-p4)*x1;
% inhomogeneous
model.sym.xdot(2) = +p2*x1*exp(-0.1*t)-p3*x2 ;
model.sym.xdot(3) = -1.5*x3;
```

INITIAL CONDITIONS

```
model.sym.x0 = sym(zeros(size(model.sym.x)));

model.sym.x0(1) = k1;
model.sym.x0(2) = k2;
model.sym.x0(3) = k3;
```

OBSERVALES

```
model.sym.y = sym(zeros(1,1));

model.sym.y(1) = p4 * (x1+x2+x3);
```

EVENTS this part is optional and can be ommited

```
syms t

% events fire when there is a zero crossing of the root function
model.event(1) = amievent(x3-x2,0,t);
model.event(2) = amievent(x3-x1,0,t);


end
```

```
ans =
    param: 'log10'
      sym: [1x1 struct]
    event: [1x2 amievent]
```

3.1.2 Simulation

```
function example_events()
```

COMPILATION

```
[exdir,~,~]=fileparts(which('example_events.m'));
% compile the model
amiwrap('model_events','model_events_syms',exdir)
```

```
Generating model struct ...
Parsing model struct ...

Generating C code ...
headers | wrapfunctions |
Compiling mex file ...
amici | Building with 'Xcode with Clang'.
MEX completed successfully.
Building with 'Xcode with Clang'.
MEX completed successfully.
```

SIMULATION

```
% time vector
t = linspace(0,10,20);
p = [0.5;2;0.5;0.5];
k = [4,8,10,4];

options = amioption('sensi',0,...
    'maxsteps',1e4,...
    'nmaxevent', 2);
D = amidata(length(t),1,2,2,4);
% load mex into memory
[~] = which('simulate_model_events'); % fix for inaccessability problems
sol = simulate_model_events(t,log10(p),k,D,options);

tic
sol = simulate_model_events(t,log10(p),k,D,options);
disp(['Time elapsed with cvodes: ' num2str(toc) ])
```

```
Time elapsed with cvodes: 0.0037064
```

ODE15S

```
ode_system = @(t,x,p,k) [-p(1)*heaviside(t-p(4))*x(1);
    +p(2)*x(1)*exp(-0.1*t)-p(3)*x(2);
    -1.5*x(3)];
% event_fn = @(t,x) [x(3) - x(2);
%     x(3) - x(1)];
% 'Events',event_fn
options_ode15s = odeset('RelTol',options.rtol,'AbsTol',options.atol,'MaxStep',options.maxsteps);

tic
[~, X_ode15s] = ode15s(@(t,x) ode_system(t,x,p,k),t,k(1:3),options_ode15s);
disp(['Time elapsed with ode15s: ' num2str(toc) ])
```

```
Time elapsed with ode15s: 0.074866
```

PLOTTING

```
figure
c_x = get(gca,'ColorOrder');
subplot(2,2,1)
for ix = 1:size(sol.x,2)
    plot(t,sol.x(:,ix),'.-','Color',c_x(ix,:))
    hold on
    plot(t,X_ode15s(:,ix),'d','Color',c_x(ix,:))
end
stem(sol.z(:,1),sol.z(:,1)*0+10,'r')
stem(sol.z(:,2),sol.z(:,2)*0+10,'k')
legend('x1','x1_ode15s','x2','x2_ode15s','x3','x3_ode15s','x3==x2','x3==x1','Location','NorthEastOutside')
legend boxoff
xlabel('time t')
ylabel('x')
box on
subplot(2,2,2)
plot(t,abs(sol.x-X_ode15s),'--')
set(gca,'YScale','log')
legend('error x1','error x2','error x3','Location','NorthEastOutside')
legend boxoff
ylabel('x')

subplot(2,2,3)
plot(t,sol.y,'.-','Color',c_x(1,:))
hold on
plot(t,p(4)*sum(X_ode15s,2),'d','Color',c_x(1,:))
legend('y1','y1_ode15s','Location','NorthEastOutside')
legend boxoff
xlabel('time t')
ylabel('y')
box on

subplot(2,2,4)
plot(t,abs(sol.y-p(4)*sum(X_ode15s,2)),'--')
set(gca,'YScale','log')
legend('error y1','Location','NorthEastOutside')
legend boxoff
xlabel('time t')
ylabel('y')
box on

set(gcf,'Position',[100 300 1200 500])
```

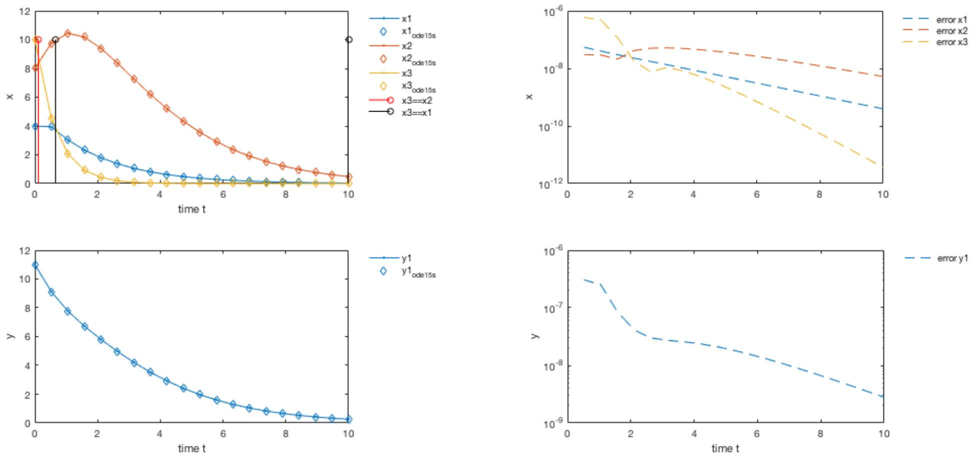

FORWARD SENSITIVITY ANALYSIS

```
options.sensi = 1;

sol = simulate_model_events(t,log10(p),k,D,options);
```

FINITE DIFFERENCES

```
eps = 1e-4;
xi = log10(p);
for ip = 1:4;
    xip = xi;
    xip(ip) = xip(ip) + eps;
    solp = simulate_model_events(t,xip,k,D,options);
    sx_fd(:,:,ip) = (solp.x - sol.x)/eps;
    sy_fd(:,:,ip) = (solp.y - sol.y)/eps;
    sz_fd(:,:,ip) = (solp.z - sol.z)/eps;
end
```

PLOTTING

```
figure
for ip = 1:4
    subplot(4,2,ip*2-1)
    hold on
    for ix = 1:size(sol.x,2)
        plot(t,sol.sx(:,ix,ip),'.-','Color',c_x(ix,:))
        plot(t,sx_fd(:,ix,ip),'d','Color',c_x(ix,:))
    end
    legend('sx1','sx1_fd','sx2','sx2_fd','sx3','sx3_fd','Location','NorthEastOutside')
    legend boxoff
    title(['state sensitivity for p' num2str(ip)])
    xlabel('time t')
    ylabel('sx')
    box on

    subplot(4,2,ip*2)
    plot(t,abs(sol.sx(:,:,ip)-sx_fd(:,:,ip)),'--')
    legend('error sx1','error sx2','error sx3','Location','NorthEastOutside')
    legend boxoff
    title(['state sensitivity for p' num2str(ip)])
    xlabel('time t')
    ylabel('error')
    set(gca,'YScale','log')
    box on
end
set(gcf,'Position',[100 300 1200 500])

figure
for ip = 1:4
    subplot(4,2,ip*2-1)
    hold on
    for iy = 1:size(sol.y,2)
        plot(t,sol.sy(:,iy,ip),'.-','Color',c_x(iy,:))
```

```
        plot(t,sy_fd(:,iy,ip),'d','Color',c_x(iy,:))
    end
    legend('sy1','sy1_fd','Location','NorthEastOutside')
    legend boxoff
    title(['observable sensitivity for p' num2str(ip)])
    xlabel('time t')
    ylabel('sy')
    box on

    subplot(4,2,ip*2)
    plot(t,abs(sol.sy(:,:,ip)-sy_fd(:,:,ip)),'--')
    legend('error sy1','Location','NorthEastOutside')
    legend boxoff
    title(['error observable sensitivity for p' num2str(ip)])
    xlabel('time t')
    ylabel('error')
    set(gca,'YScale','log')
    box on
end
set(gcf,'Position',[100 300 1200 500])

figure
for ip = 1:4
subplot(4,2,2*ip-1)
bar(1:options.nmaxevent,sol.sz(1:options.nmaxevent,:,ip),0.8)
hold on
bar(1:options.nmaxevent,sz_fd(1:options.nmaxevent,:,ip),0.4)
legend('x3==x2','x3==x1','x3==x2 fd','x3==x1 fd','Location','NorthEastOutside')
legend boxoff
title(['event sensitivity for p' num2str(ip)])
xlabel('event #')
ylabel('sz')
box on

subplot(4,2,2*ip)
bar(1:options.nmaxevent,sol.sz(1:options.nmaxevent,:,ip)-sz_fd(1:options.nmaxevent,:,ip),0.8)
legend('error x3==x2','error x3==x1','Location','NorthEastOutside')
legend boxoff
title(['error event sensitivity for p' num2str(ip)])
xlabel('event #')
ylabel('sz')
box on
end
set(gcf,'Position',[100 300 1200 500])

drawnow
```

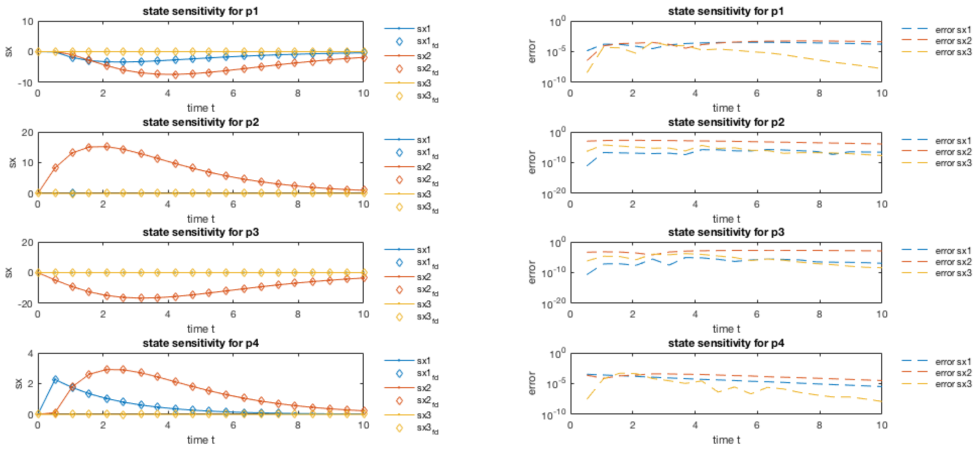

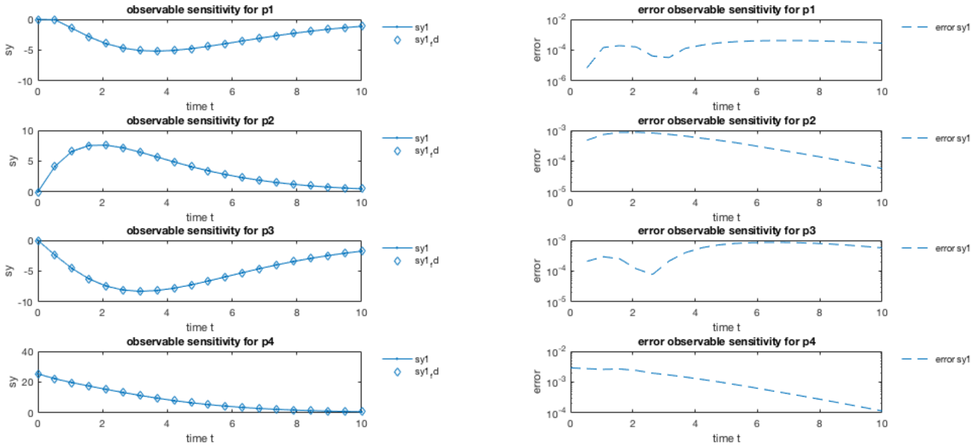

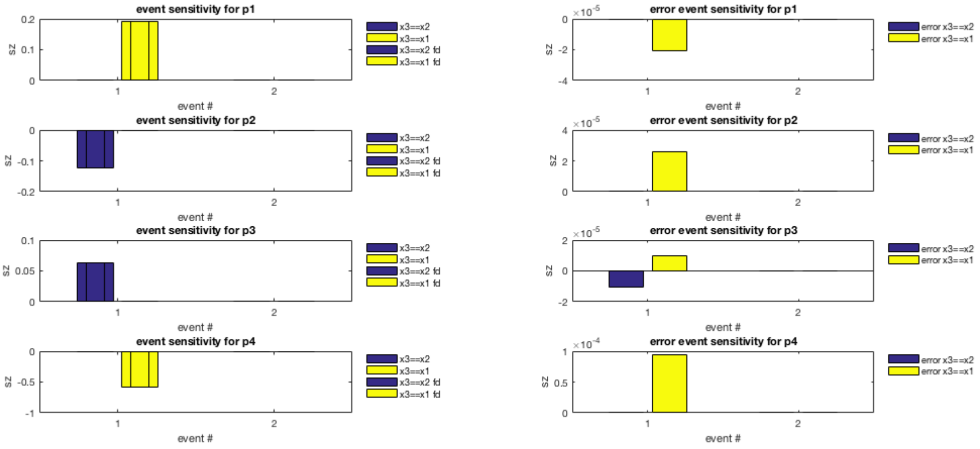


```
end
```

3.2 Example Dirac

3.2.1 Model Definition

```
function [model] = model_dirac_syms()
```

STATES

```
% create state syms
syms x1 x2

% create state vector
model.sym.x = [ x1 x2 ];
```

PARAMETERS (for these sensitivities will be computed)

```
% create parameter syms
syms p1 p2 p3 p4

% create parameter vector
model.sym.p = [p1,p2,p3,p4];

% set the parametrisation of the problem options are 'log', 'log10' and
% 'lin' (default).
model.param = 'log10';
```

SYSTEM EQUATIONS

```
% create symbolic variable for time
syms t

model.sym.xdot = sym(zeros(size(model.sym.x)));

% piecewise defined function
model.sym.xdot(1) = -p1*x1 + dirac(t-p2);
% inhomogeneous
model.sym.xdot(2) = p3*x1 - p4*x2 ;
```

INITIAL CONDITIONS

```
model.sym.x0 = sym(zeros(size(model.sym.x)));

model.sym.x0(1) = 0;
model.sym.x0(2) = 0;
```

OBSERVALES

```
model.sym.y = sym(zeros(1,1));

model.sym.y(1) = x2;


end
```

```
ans =
      sym: [1x1 struct]
    param: 'log10'
```

3.2.2 Simulation

```
function example_dirac()
```

COMPILATION

```
    [exdir,~,~]=fileparts(which('example_dirac.m'));
    % compile the model
    amiwrap('model_dirac','model_dirac_syms',exdir)
```

```
Generating model struct ...
Parsing model struct ...

Generating C code ...
headers | wrapfunctions |
Compiling mex file ...
amici | Building with 'Xcode with Clang'.
MEX completed successfully.
Building with 'Xcode with Clang'.
MEX completed successfully.
```

SIMULATION

```
    % time vector
    t = linspace(0,3,1001);
    p = [1;0.5;2;3];
    k = [];

    options = amioption('sensi',0,...
        'maxsteps',1e4);

    % load mex into memory
    [msg] = which('simulate_model_dirac'); % fix for inaccessability problems
    sol = simulate_model_dirac(t,log10(p),k,[],options);

    tic
    sol = simulate_model_dirac(t,log10(p),k,[],options);
    disp(['Time elapsed with amiwrap: ' num2str(toc) ])
```

```
Time elapsed with amiwrap: 0.0045507
```

ODE15S

```
sig = 1e-2;
delta_num = @(tau) exp(-1/2*(tau/sig).^2)/(sqrt(2*pi)*sig);

ode_system = @(t,x,p,k) [-p(1)*x(1)+delta_num(t-p(2));
    +p(3)*x(1) - p(4)*x(2)];

options_ode45 = odeset('RelTol',options.rtol,'AbsTol',options.atol,'MaxStep',options.maxsteps);

tic
[~, X_ode45] = ode45(@(t,x) ode_system(t,x,p,k),t,[0;0],options_ode45);
disp(['Time elapsed with ode45: ' num2str(toc) ])
```

```
Time elapsed with ode45: 0.080371
```

PLOTTING

```
figure
c_x = get(gca,'ColorOrder');
subplot(2,2,1)
for ix = 1:size(sol.x,2)
    plot(t,sol.x(:,ix),'.-','Color',c_x(ix,:))
    hold on
    plot(t,X_ode45(:,ix),'--','Color',c_x(ix,:))
end

legend('x1','x1_ode45','x2','x2_ode15s','Location','NorthEastOutside')
legend boxoff
xlabel('time t')
ylabel('x')
box on
subplot(2,2,2)
plot(t,abs(sol.x-X_ode45),'--')
set(gca,'YScale','log')
ylim([1e-10,1e0])
legend('error x1','error x2','Location','NorthEastOutside')
legend boxoff

subplot(2,2,3)
plot(t,sol.y,'.-','Color',c_x(1,:))
hold on
plot(t,X_ode45(:,2),'--','Color',c_x(1,:))
legend('y1','y1_ode45','Location','NorthEastOutside')
legend boxoff
xlabel('time t')
ylabel('y')
box on

subplot(2,2,4)
plot(t,abs(sol.y-X_ode45(:,2)),'--')
set(gca,'YScale','log')
ylim([1e-10,1e0])
legend('error y1','Location','NorthEastOutside')
legend boxoff
xlabel('time t')
ylabel('y')
box on
set(gcf,'Position',[100 300 1200 500])
```

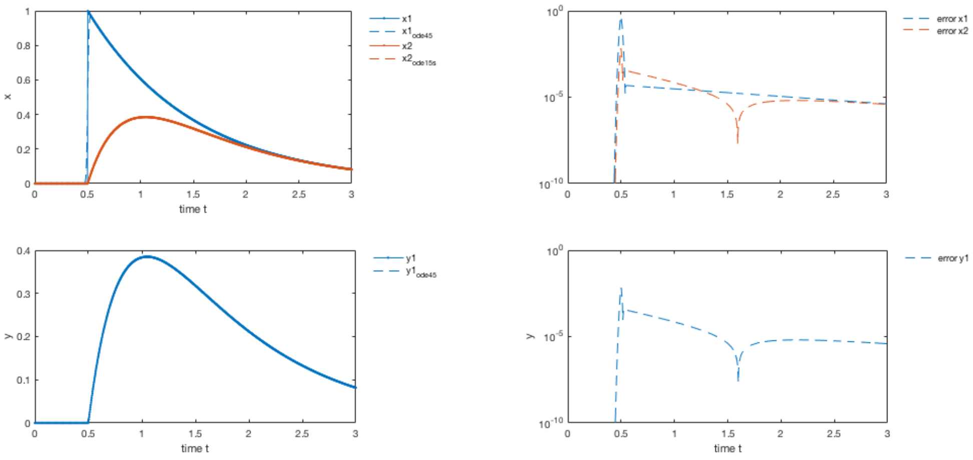

FORWARD SENSITIVITY ANALYSIS

```
options.sensi = 1;

sol = simulate_model_dirac(t,log10(p),k,[],options);
```

FINITE DIFFERENCES

```
eps = 1e-4;
xi = log10(p);
for ip = 1:4;
    xip = xi;
    xip(ip) = xip(ip) + eps;
    solp = simulate_model_dirac(t,xip,k,[],options);
    sx_fd(:,:,ip) = (solp.x - sol.x)/eps;
    sy_fd(:,:,ip) = (solp.y - sol.y)/eps;
end
```

PLOTTING

```
figure
for ip = 1:4
    subplot(4,2,ip*2-1)
    hold on
    for ix = 1:size(sol.x,2)
        plot(t,sol.sx(:,ix,ip),'.-','Color',c_x(ix,:))
        plot(t,sx_fd(:,ix,ip),'--','Color',c_x(ix,:))
    end
    ylim([-2,2])
    legend('x1','x1_fd','x2','x2_fd','Location','NorthEastOutside')
    legend boxoff
    title(['state sensitivity for p' num2str(ip)])
    xlabel('time t')
    ylabel('x')
    box on

    subplot(4,2,ip*2)
    plot(t,abs(sol.sx(:,:,ip)-sx_fd(:,:,ip)),'r--')
    legend('error x1','error x2','Location','NorthEastOutside')
    legend boxoff
    title(['state sensitivity for p' num2str(ip)])
    xlabel('time t')
    ylabel('error')
    ylim([1e-12,1e0])
    set(gca,'YScale','log')
    box on
end
set(gcf,'Position',[100 300 1200 500])

figure
for ip = 1:4
    subplot(4,2,ip*2-1)
    hold on
    for iy = 1:size(sol.y,2)
```

```
            plot(t,sol.sy(:,iy,ip),'.-','Color',c_x(iy,:))
            plot(t,sy_fd(:,iy,ip),'--','Color',c_x(iy,:))
        end
        ylim([-2,2])
        legend('y1','y1_fd','Location','NorthEastOutside')
        legend boxoff
        title(['observable sensitivity for p' num2str(ip)])
        xlabel('time t')
        ylabel('y')
        box on

        subplot(4,2,ip*2)
        plot(t,abs(sol.sy(:,:,ip)-sy_fd(:,:,ip)),'r--')
        legend('error y1','Location','NorthEastOutside')
        legend boxoff
        title(['observable sensitivity for p' num2str(ip)])
        xlabel('time t')
        ylabel('error')
        ylim([1e-12,1e0])
        set(gca,'YScale','log')
        box on
    end
    set(gcf,'Position',[100 300 1200 500])

    drawnow
```

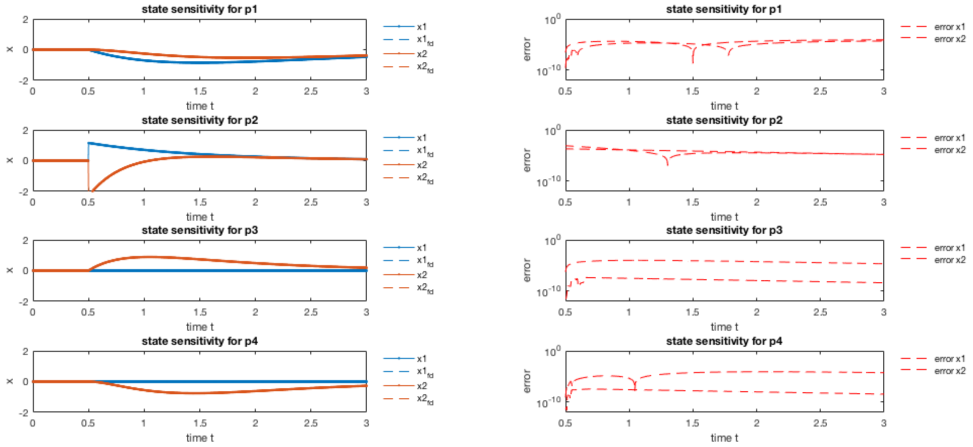

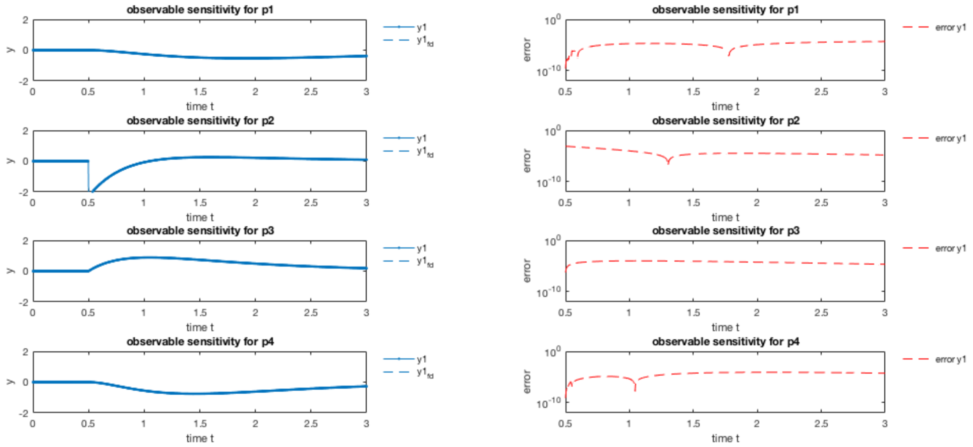


```
end
```

3.3 Example Steady State

3.3.1 Model Definition

```
function [model] = model_steadystate_syms()
```

STATES

```
% create state syms
syms x1 x2 x3

% create state vector
model.sym.x = [
x1 x2 x3
];
```

PARAMETERS (for these sensitivities will be computed)

```
% create parameter syms
syms p1 p2 p3 p4 p5

% create parameter vector
model.sym.p = [p1,p2,p3,p4,p5];

% set the parametrisation of the problem options are 'log', 'log10' and
% 'lin' (default).
model.param = 'log10';
```

CONSTANTS (for these no sensitivities will be computed) this part is optional and can be ommited

```
% create parameter syms
syms k1 k2 k3 k4

% create parameter vector
model.sym.k = [k1 k2 k3 k4];
```

SYSTEM EQUATIONS

```
% create symbolic variable for time
syms t

model.sym.xdot = sym(zeros(size(model.sym.x)));

% piecewise defined function
model.sym.xdot(1) = -2*p1*x1^2 - p2*x1*x2 + 2*p3*x2 + p4*x3 + p5;
% inhomogeneous
model.sym.xdot(2) = +p1*x1^2 - p2*x1*x2 - p3*x2 + p4*x3;
model.sym.xdot(3) = p2*x1*x2 - p4*x3 - k4*x3;
```

INITIAL CONDITIONS

```
model.sym.x0 = sym(zeros(size(model.sym.x)));

model.sym.x0(1) = k1;
model.sym.x0(2) = k2;
model.sym.x0(3) = k3;
```

OBSERVALES

```
model.sym.y = model.sym.x;
```

```
end
```

```
ans =
      sym: [1x1 struct]
    param: 'log10'
```

3.3.2 Simulation

```
function example_steadystate
```

COMPILATION

```
    [exdir,~,~]=fileparts(which('example_steadystate.m'));
    % compile the model
    amiwrap('model_steadystate','model_steadystate_syms',exdir)
```

```
Generating model struct ...
Parsing model struct ...

Generating C code ...
headers | wrapfunctions |
Compiling mex file ...
amici | Building with 'Xcode with Clang'.
MEX completed successfully.
Building with 'Xcode with Clang'.
MEX completed successfully.
```

SIMULATION

```
    % time vector
    t = linspace(0,300,20);
    p = [1;0.5;0.4;2;0.1];
    k = [0.1,0.4,0.7,1];

    options = amioption('sensi',0,...
        'maxsteps',1e4);
    % load mex into memory
    sol = simulate_model_steadystate(t,log10(p),k,[],options);

    tic
    sol = simulate_model_steadystate(t,log10(p),k,[],options);
    disp(['Time elapsed with cvodes: ' num2str(toc) ])
```

```
Time elapsed with cvodes: 0.004374
```

ODE15S

```
    ode_system = @(t,x,p,k) [-2*p(1)*x(1)^2 - p(2)*x(1)*x(2) + 2*p(3)*x(2) + p(4)*x(3) + p(5);
        + p(1)*x(1)^2 - p(2)*x(1)*x(2) - p(3)*x(2) + p(4)*x(3);
        + p(2)*x(1)*x(2) - p(4)*x(3) - k(4)*x(3)];
    options_ode15s = odeset('RelTol',options.rtol,'AbsTol',options.atol,'MaxStep',options.maxsteps);

    tic
    [~, X_ode15s] = ode15s(@(t,x) ode_system(t,x,p,k),t,k(1:3),options_ode15s);
    disp(['Time elapsed with ode15s: ' num2str(toc) ])
```

```
Time elapsed with ode15s: 0.066508
```

PLOTTING

```
    figure
    c_x = get(gca,'ColorOrder');
    subplot(2,2,1)
    for ix = 1:size(sol.x,2)
        plot(t,sol.x(:,ix),'.-','Color',c_x(ix,:))
        hold on
        plot(t,X_ode15s(:,ix),'d','Color',c_x(ix,:))
    end
    legend('x1','x1_ode15s','x2','x2_ode15s','x3','x3_ode15s','Location','NorthEastOutside')
    legend boxoff
    xlabel('time t')
    ylabel('x')
    box on
    subplot(2,2,2)
    plot(t,abs(sol.x-X_ode15s),'--')
    set(gca,'YScale','log')
    legend('error x1','error x2','error x3','Location','NorthEastOutside')
    legend boxoff
    set(gcf,'Position',[100 300 1200 500])
```

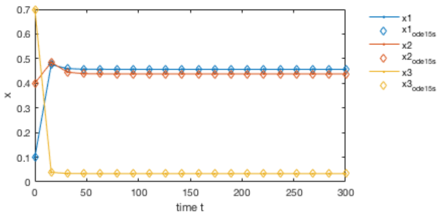

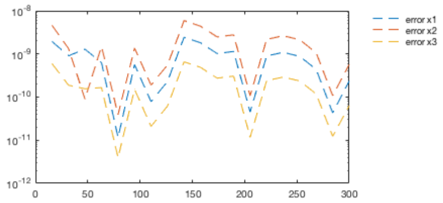

FORWARD SENSITIVITY ANALYSIS

```
options.sensi = 1;
options.sens_ind = [3,1,2,4];

sol = simulate_model_steadystate(t,log10(p),k,[],options);
```

FINITE DIFFERENCES

```
eps = 1e-3;

xi = log10(p);
for ip = 1:4;
    xip = xi;
    xip(ip) = xip(ip) + eps;
    solp = simulate_model_steadystate(t,xip,k,[],options);
    sx_fd(:,:,ip) = (solp.x - sol.x)/eps;
    sy_fd(:,:,ip) = (solp.y - sol.y)/eps;
end
```

PLOTTING

```
figure
for ip = 1:4
    subplot(4,2,ip*2-1)
    hold on
    for ix = 1:size(sol.x,2)
        plot(t,sol.sx(:,ix,ip),'.-','Color',c_x(ix,:))
        plot(t,sx_fd(:,ix,options.sens_ind(ip)),'d','Color',c_x(ix,:))
    end
    legend('x1','x1_fd','x2','x2_fd','x3','x3_fd','Location','NorthEastOutside')
    legend boxoff
    title(['state sensitivity for p' num2str(options.sens_ind(ip))])
    xlabel('time t')
    ylabel('x')
    box on

    subplot(4,2,ip*2)
    plot(t,abs(sol.sx(:,:,ip)-sx_fd(:,:,options.sens_ind(ip))),'--')
    legend('error x1','error x2','error x3','Location','NorthEastOutside')
    legend boxoff
    title(['error of state sensitivity for p' num2str(options.sens_ind(ip))])
    xlabel('time t')
    ylabel('error')
    set(gca,'YScale','log')
    box on
end
set(gcf,'Position',[100 300 1200 500])
```

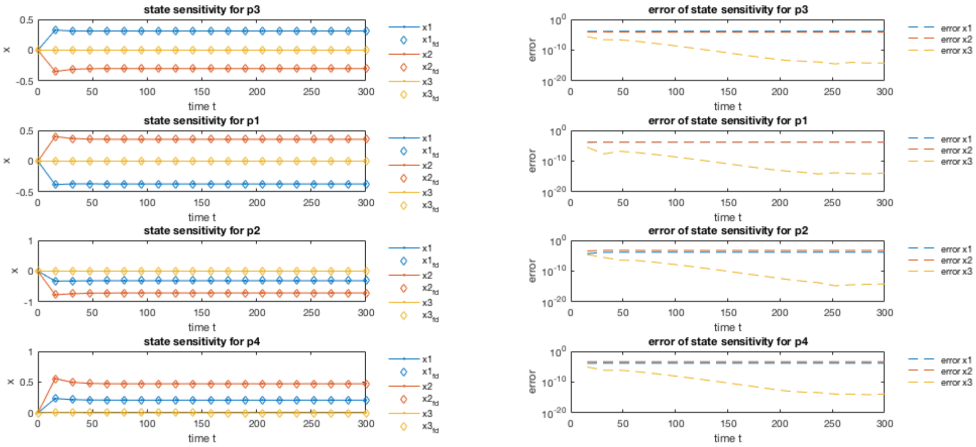

STEADY STATE SENSITIVITY

```
sssens = NaN(size(sol.sx));
for it = 2:length(t)
    tt = [0,t(it)];
    options.sensi_meth = 'ss';
    solss = simulate_model_steadystate(tt,log10(p),k,[],options);
    sssens(it,:,:) = solss.sx;
    ssxdot(it,:) = solss.xdot;
end
```

PLOTTING

```
figure
for ip = 1:4
    subplot(4,2,ip*2-1)
    hold on
    for ix = 1:size(sol.x,2)
        plot(t,sol.sx(:,ix,ip),'.-','Color',c_x(ix,:))
        plot(t,sssens(:,ix,ip),'d-','Color',c_x(ix,:))
    end
    legend('x1','x1_ss','x2','x2_ss','x3','x3_ss','Location','NorthEastOutside')
    legend boxoff
    title(['state steady sensitivity for p' num2str(ip)])
    xlabel('time t')
    ylabel('x')
    box on

    subplot(4,2,ip*2)
    plot(t,abs(sol.sx(:,:,ip)-sssens(:,:,ip)),'--')
    legend('error x1','error x2','error x3','Location','NorthEastOutside')
    legend boxoff
    title(['error of steady state sensitivity for p' num2str(ip)])
    xlabel('time t')
    ylabel('error')
    set(gca,'YScale','log')
    box on
end
set(gcf,'Position',[100 300 1200 500])

figure
scatter(sqrt(sum((ssxdot./sol.x).^2,2)),sqrt(sum(sum((sol.sx-sssens).^2,2),3)))
hold on
plot([1e-15,1e5],[1e-15,1e5],'k:')
set(gca,'YScale','log')
set(gca,'XScale','log')
box on
axis square
xlabel('||dxdt/x||_2')
ylabel('error steady state approximation')
set(gca,'FontSize',15)
set(gca,'LineWidth',1.5)
set(gcf,'Position',[100 300 1200 500])

drawnow
```

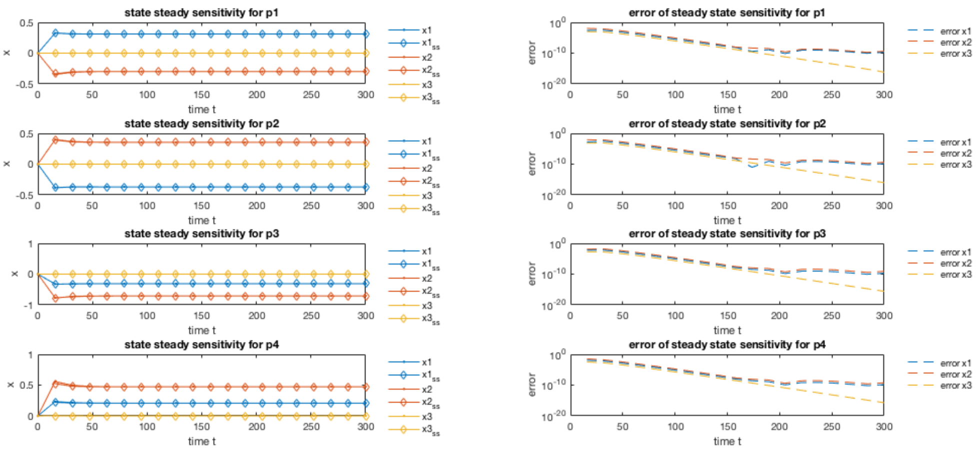

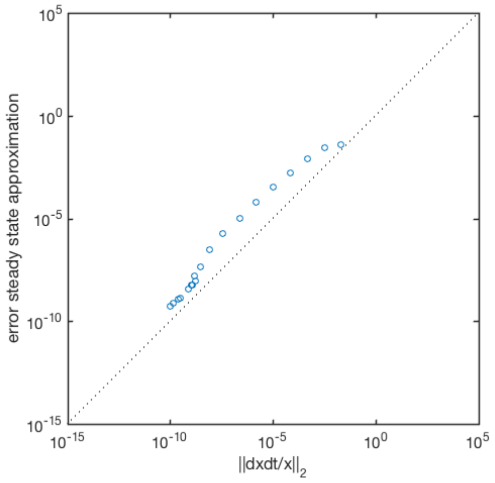


```
end
```

3.4 Example JAK/STAT Adjoint

3.4.1 Model Definition

```
function [model] = model_jakstat_syms()
```

STATES

```
    syms STAT pSTAT pSTAT_pSTAT npSTAT_npSTAT nSTAT1 nSTAT2 nSTAT3 nSTAT4 nSTAT5

    model.sym.x = [
        STAT, pSTAT, pSTAT_pSTAT, npSTAT_npSTAT, nSTAT1, nSTAT2, nSTAT3, nSTAT4, nSTAT5 ...
        ];
```

PARAMETERS

```
    syms p1 p2 p3 p4 init_STAT Omega_cyt Omega_nuc sp1 sp2 sp3 sp4 sp5 offset_tSTAT offset_pSTAT scale_tSTAT scale_pSTAT sigma

    model.sym.p = [p1,p2,p3,p4,init_STAT,sp1,sp2,sp3,sp4,sp5,offset_tSTAT,offset_pSTAT,scale_tSTAT,scale_pSTAT,sigma_pSTAT,sig

    model.param = 'log10';

    model.sym.k = [Omega_cyt,Omega_nuc];
```

INPUT

```
    syms t
    u(1) = spline_pos5(t, 0.0, sp1, 5.0, sp2, 10.0, sp3, 20.0, sp4, 60.0, sp5, 0, 0.0);


Warning: Support of strings that are not valid variable names or define a number
will be removed in a future release. To create symbolic expressions, first
create symbolic variables and then use operations on them.
```

SYSTEM EQUATIONS

```
    model.sym.xdot = sym(zeros(size(model.sym.x)));

    model.sym.xdot(1) = (Omega_nuc*p4*nSTAT5 - Omega_cyt*STAT*p1*u(1))/Omega_cyt;
    model.sym.xdot(2) = STAT*p1*u(1) - 2*p2*pSTAT^2;
    model.sym.xdot(3) = p2*pSTAT^2 - p3*pSTAT_pSTAT;
    model.sym.xdot(4) = -(Omega_nuc*p4*npSTAT_npSTAT - Omega_cyt*p3*pSTAT_pSTAT)/Omega_nuc;
    model.sym.xdot(5) = -p4*(nSTAT1 - 2*npSTAT_npSTAT);
    model.sym.xdot(6) = p4*(nSTAT1 - nSTAT2);
    model.sym.xdot(7) = p4*(nSTAT2 - nSTAT3);
    model.sym.xdot(8) = p4*(nSTAT3 - nSTAT4);
    model.sym.xdot(9) = p4*(nSTAT4 - nSTAT5);
```

INITIAL CONDITIONS

```
    model.sym.x0 = sym(zeros(size(model.sym.x)));

    model.sym.x0(1) = init_STAT;
```

OBSERVABLES

```
    model.sym.y = sym(zeros(3,1));

    model.sym.y(1) = offset_pSTAT + scale_pSTAT/init_STAT*(pSTAT + 2*pSTAT_pSTAT);
    model.sym.y(2) = offset_tSTAT + scale_tSTAT/init_STAT*(STAT + pSTAT + 2*(pSTAT_pSTAT));
    model.sym.y(3) = u(1);
```

SIGMA

```
    model.sym.sigma_y = sym(size(model.sym.y));

    model.sym.sigma_y(1) = sigma_pSTAT;
    model.sym.sigma_y(2) = sigma_tSTAT;
    model.sym.sigma_y(3) = sigma_pEpoR;


end


ans =
      sym: [1x1 struct]
    param: 'log10'
```

3.4.2 Simulation

```
function example_jakstat_adjoint()

    % compile the model
    [exdir,~,~]=fileparts(which('example_jakstat_adjoint.m'));
    amiwrap('model_jakstat','model_jakstat_adjoint_syms',exdir)

    num = xlsread(fullfile(exdir,'pnas_data_original.xls'));

    D.t = num(:,1);
    D.condition= [1.4,0.45];
    D.Y = num(:,[2,4,6]);
    D.Sigma_Y = NaN(size(D.Y));
    D = amidata(D);
```

```
    xi =  [0.60
        3
        -0.95
        -0.0075
        0
        -2.8
        -0.26
        -0.075
        -0.41
        -5
        -0.74
        -0.64
        -0.11
        0.027
        -0.5
        0
        -0.5];

    options.sensi = 0;
    sol = simulate_model_jakstat([],xi,[],D,options);

    figure
    for iy = 1:3
        subplot(2,2,iy)
        plot(D.t,D.Y(:,iy),'rx')
        hold on
        plot(sol.t,sol.y(:,iy),'.-')
        xlim([0,60])
        xlabel('t')
        switch(iy)
            case 1
                ylabel('pStat')
            case 2
                ylabel('tStat')
            case 3
                ylabel('pEpoR')
        end
        ylim([0,1.2])
    end
    set(gcf,'Position',[100 300 1200 500])

    % generate new
    xi_rand = xi + 0.1;
    options.sensi = 1;
    options.sensi_meth = 'adjoint';
    sol = simulate_model_jakstat([],xi_rand,[],D,options);

    options.sensi = 0;
    eps = 1e-4;
    fd_grad = NaN(length(xi),1);
    for ip = 1:length(xi)
        xip = xi_rand;
        xip(ip) = xip(ip) + eps;
        psol = simulate_model_jakstat([],xip,[],D,options);
        fd_grad(ip) = (psol.llh-sol.llh)/eps;
    end

    figure
    scatter(abs(sol.sllh),abs(fd_grad))
    set(gca,'XScale','log')
    set(gca,'YScale','log')
    xlim([1e-2,1e2])
    ylim([1e-2,1e2])
    box on
    hold on
    axis square
    plot([1e-2,1e2],[1e-2,1e2],'k:')
    xlabel('adjoint sensitivity absolute value of gradient element')
    ylabel('finite difference absolute value of gradient element')
    set(gcf,'Position',[100 300 1200 500])


    drawnow

end
```

```
Generating model struct ...
Parsing model struct ...

Generating C code ...
headers | wrapfunctions |
Compiling mex file ...
amici | Building with 'Xcode with Clang'.
MEX completed successfully.
Building with 'Xcode with Clang'.
MEX completed successfully.
```

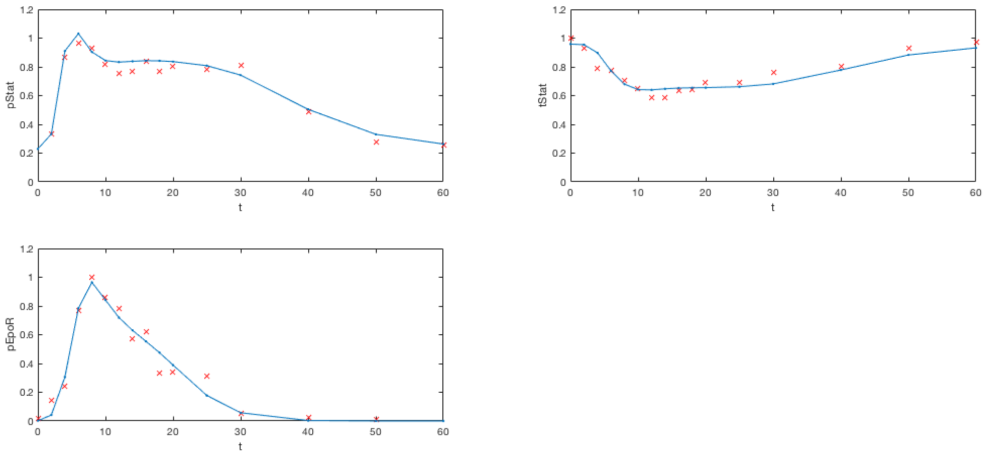

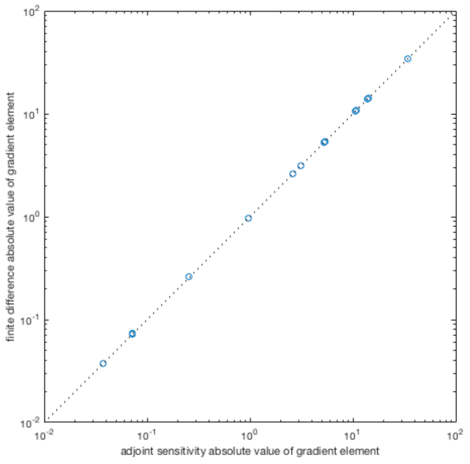

3.5 Example Dirac Adjoint

3.5.1 Model Definition

```
function [model] = model_dirac_adjoint_syms()
```

STATES

```
% create state syms
syms x1 x2

% create state vector
model.sym.x = [ x1 x2 ];
```

PARAMETERS (for these sensitivities will be computed)

```
% create parameter syms
syms p1 p2 p3 p4

% create parameter vector
model.sym.p = [p1,p2,p3,p4];

% set the parametrisation of the problem options are 'log', 'log10' and
% 'lin' (default).
model.param = 'log10';
```

SYSTEM EQUATIONS

```
% create symbolic variable for time
syms t

model.sym.xdot = sym(zeros(size(model.sym.x)));

% piecewise defined function
model.sym.xdot(1) = -p1*x1 + dirac(t-p2);
% inhomogeneous
model.sym.xdot(2) = p3*x1 - p4*x2 ;
```

INITIAL CONDITIONS

```
model.sym.x0 = sym(zeros(size(model.sym.x)));

model.sym.x0(1) = 0;
model.sym.x0(2) = 0;
```

OBSERVALES

```
model.sym.y = sym(zeros(1,1));

model.sym.y(1) = x2;


end


ans =
      sym: [1x1 struct]
    param: 'log10'
```

3.5.2 Simulation

```
function example_dirac_adjoint()
```

COMPILATION

```
    [exdir,~,~]=fileparts(which('example_dirac_adjoint.m'));
    % compile the model
    amiwrap('model_dirac_adjoint','model_dirac_adjoint_syms',exdir)


Generating model struct ...
Parsing model struct ...

Generating C code ...
headers | wrapfunctions |
Compiling mex file ...
amici | Building with 'Xcode with Clang'.
MEX completed successfully.
Building with 'Xcode with Clang'.
MEX completed successfully.
```

SIMULATION

```
    % time vector
    tout = linspace(0,4,9);
    tfine = linspace(0,4,10001);
    p = [1;0.4;2;3];
    k = [];

    D.Y = [  0.00714742903826096
        -0.00204966058299775
        0.382159034587845
        0.33298932672138
        0.226111476113441
        0.147028440865854
        0.0882468698791813
        0.0375887796628869
        0.0373422340295005];

    D.Sigma_Y = 0.01*ones(size(D.Y));
```

```
options.sensi = 1;
options.sensi_meth = 'adjoint';
options.maxsteps = 1e5;
sol = simulate_model_dirac_adjoint(tout,log10(p),k,D,options);
options.sensi = 0;
solfine = simulate_model_dirac_adjoint(tfine,log10(p),k,[],options);
figure
errorbar(tout,D.Y,D.Sigma_Y)
hold on
plot(tfine,solfine.y)
legend('data','simulation')
xlabel('time t')
ylabel('observable')
title(['log-likelihood: ' num2str(sol.llh) ])
```

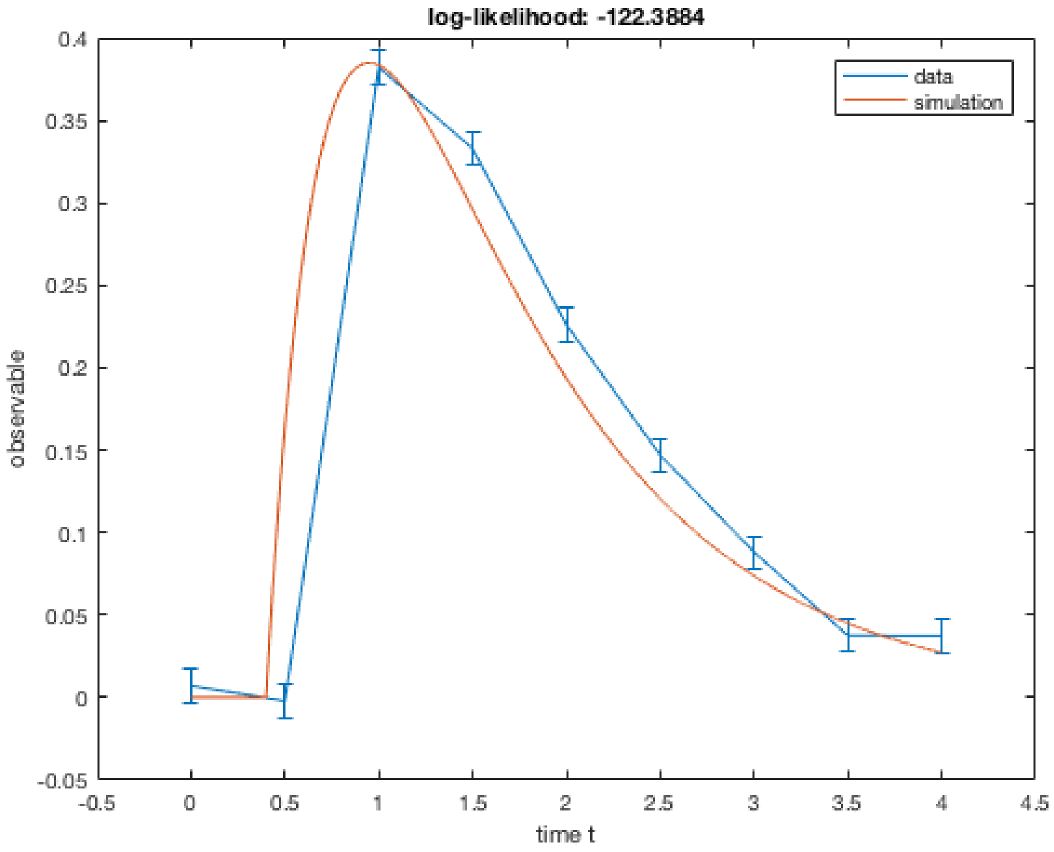

FD

```
eps = 1e-4;
xi = log10(p);
grad_fd_f = NaN(4,1);
grad_fd_b = NaN(4,1);
for ip = 1:4;
    options.sensi = 0;
    xip = xi;
    xip(ip) = xip(ip) + eps;
    solpf = simulate_model_dirac_adjoint(tout,xip,k,D,options);
    grad_fd_f(ip,1) = (solpf.llh-sol.llh)/eps;
    xip = xi;
    xip(ip) = xip(ip) - eps;
    solpb = simulate_model_dirac_adjoint(tout,xip,k,D,options);
    grad_fd_b(ip,1) = -(solpb.llh-sol.llh)/eps;
end

figure
plot(abs(grad_fd_f),abs(sol.sllh),'o')
hold on
plot(abs(grad_fd_b),abs(sol.sllh),'o')
set(gca,'XScale','log')
set(gca,'YScale','log')
```

```
    hold on
    axis square
    plot([1e2,1e4],[1e2,1e4],'k:')
    xlim([1e2,1e4])
    ylim([1e2,1e4])
    legend('forward FD','backward FD','Location','SouthEast')
    xlabel('adjoint sensitivity absolute value of gradient element')
    ylabel('computed absolute value of gradient element')
    set(gcf,'Position',[100 300 1200 500])

    drawnow
```

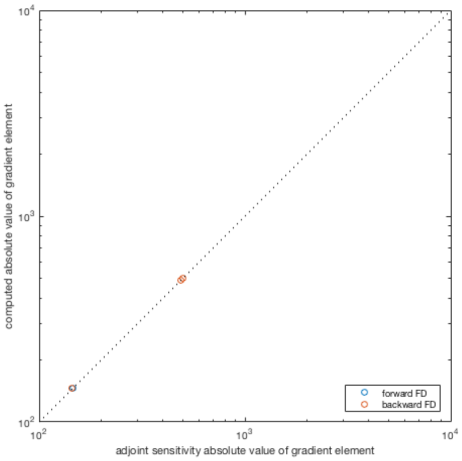


```
end
```

3.6 Example Dirac Second Order Forward

3.6.1 Model Definition

```
function [model] = model_dirac_secondorder_syms()
```

STATES

```
% create state syms
syms x1 x2

% create state vector
model.sym.x = [ x1 x2 ];
```

PARAMETERS (for these sensitivities will be computed)

```
% create parameter syms
syms p1 p2 p3 p4

% create parameter vector
model.sym.p = [p1,p2,p3,p4];

% set the parametrisation of the problem options are 'log', 'log10' and
% 'lin' (default).
model.param = 'log10';
```

SYSTEM EQUATIONS

```
% create symbolic variable for time
syms t

model.sym.xdot = sym(zeros(size(model.sym.x)));
```

```
% piecewise defined function
model.sym.xdot(1) = -p1*x1 + dirac(t-p2);
% inhomogeneous
model.sym.xdot(2) = p3*x1 - p4*x2 ;
```

INITIAL CONDITIONS

```
model.sym.x0 = sym(zeros(size(model.sym.x)));

model.sym.x0(1) = 0;
model.sym.x0(2) = 0;
```

OBSERVALES

```
model.sym.y = sym(zeros(1,1));

model.sym.y(1) = x2;


end
```

```
ans =
      sym: [1x1 struct]
    param: 'log10'
```

3.6.2 Simulation

```
function example_dirac_secondorder()
```

COMPILATION

```
    [exdir,~,~]=fileparts(which('example_dirac_secondorder.m'));
    % compile the model
    amiwrap('model_dirac_secondorder','model_dirac_secondorder_syms',exdir,1)
```

```
Generating model struct ...
x | k | p | deltax | xdot | deltaxdot | ddeltaxdx | ddeltaxdp | ddeltaxdt | root | drootdx | sx | drootdp | drootdt | dtaudp
z |

Generating C code ...
deltasx | deltax | dsigma_ydp | dsigma_zdp | dydp | dzdp | root | sigma_y | sigma_z | stau | xdot | y | z | headers | wrapfunc
headers | wrapfunctions |
Compiling mex file ...
amici | Building with 'Xcode with Clang'.
MEX completed successfully.
Building with 'Xcode with Clang'.
MEX completed successfully.
amici | Building with 'Xcode with Clang'.
MEX completed successfully.
Building with 'Xcode with Clang'.
MEX completed successfully.
```

SIMULATION

```
    % time vector
    t = linspace(0,3,1001);
    p = [1;0.5;2;3];
    k = [];

    options = amioption('sensi',0,...
        'maxsteps',1e4);

    % load mex into memory
    [msg] = which('simulate_model_secondorder_dirac'); % fix for inaccessability problems
    options.sensi = 2;
    sol = simulate_model_dirac_secondorder(t,log10(p),k,[],options);
```

FORWARD SENSITIVITY ANALYSIS

```
options.sensi = 2;

sol = simulate_model_dirac_secondorder(t,log10(p),k,[],options);
```

FINITE DIFFERENCES

```
options.sensi = 1;

eps = 1e-4;
xi = log10(p);
for ip = 1:4;
    xip = xi;
    xip(ip) = xip(ip) + eps;
    solp = simulate_model_dirac_secondorder(t,xip,k,[],options);
    s2x_fd(:,:,:,ip) = (solp.sx - sol.sx)/eps;
    s2y_fd(:,:,:,ip) = (solp.sy - sol.sy)/eps;
end
```

PLOTTING

```
figure
c_x = get(gca,'ColorOrder');
for ip = 1:4
    for jp = 1:4
        subplot(4,4,(ip-1)*4+jp)
        hold on
        for ix = 1:size(sol.x,2)
            plot(t,sol.s2x(:,ix,ip,jp),'.-','Color',c_x(ix,:))
            plot(t,s2x_fd(:,ix,ip,jp),'--','Color',c_x(ix,:))
        end
        ylim([-10,10])
        legend('x1','x1_fd','x2','x2_fd','Location','NorthEastOutside')
        legend boxoff
        title(['state sensitivity for p' num2str(ip) '/p' num2str(jp)])
        xlabel('time t')
        ylabel('x')
        box on
    end
end
set(gcf,'Position',[100 300 1200 500])
figure
for ip = 1:4
    for jp = 1:4
        subplot(4,4,(ip-1)*4+jp)
        plot(t,abs(sol.s2x(:,:,ip,jp)-s2x_fd(:,:,ip,jp)),'r--')
        legend('error x1','error x2','Location','NorthEastOutside')
        legend boxoff
        title(['state sensitivity for p' num2str(ip) '/p' num2str(jp)])
        xlabel('time t')
        ylabel('error')
        ylim([1e-12,1e0])
        set(gca,'YScale','log')
        box on
    end
end
set(gcf,'Position',[100 300 1200 500])

drawnow
```

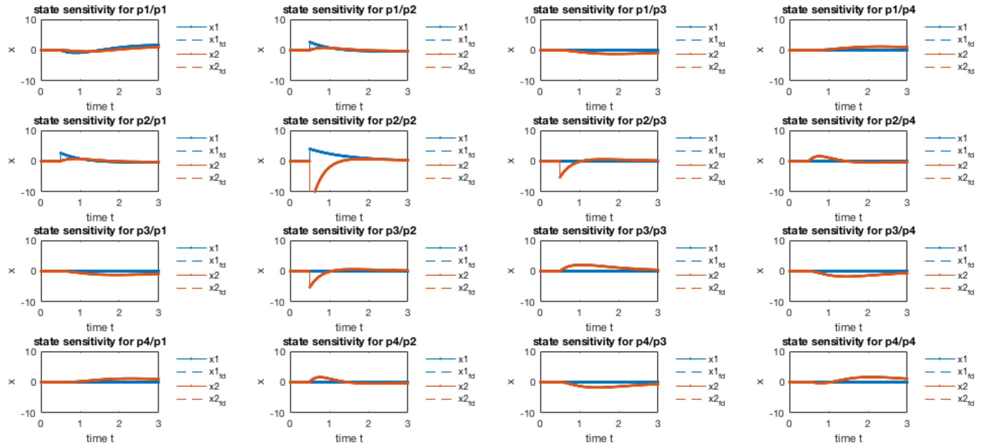

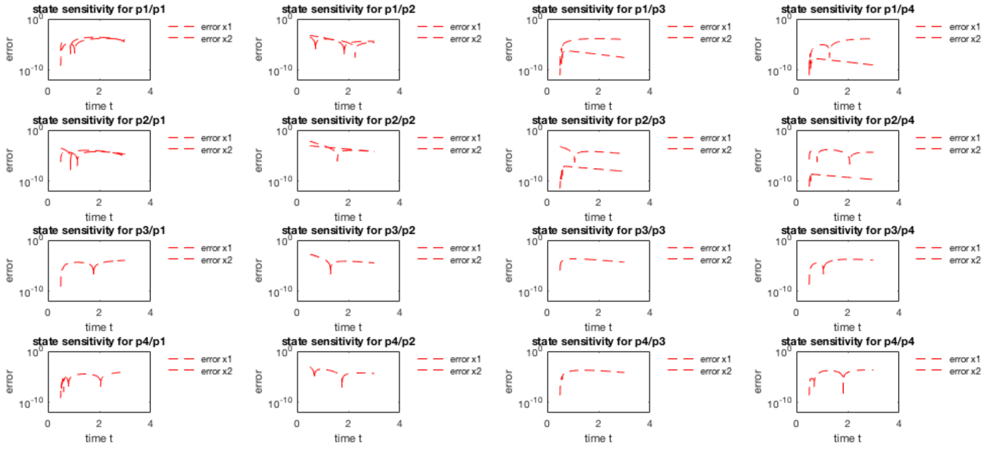


```
end
```

3.7 Example Dirac Directional Second Order Forward

3.7.1 Model Definition

```
function [model] = model_dirac_secondorder_vectmult_syms()
```

STATES

```
% create state syms
syms x1 x2

% create state vector
model.sym.x = [ x1 x2 ];
```

PARAMETERS (for these sensitivities will be computed)

```
% create parameter syms
syms p1 p2 p3 p4

% create parameter vector
model.sym.p = [p1,p2,p3,p4];

% set the parametrisation of the problem options are 'log', 'log10' and
% 'lin' (default).
model.param = 'log10';
```

SYSTEM EQUATIONS

```
% create symbolic variable for time
syms t

model.sym.xdot = sym(zeros(size(model.sym.x)));

% piecewise defined function
model.sym.xdot(1) = -p1*x1 + dirac(t-p2);
% inhomogeneous
model.sym.xdot(2) = p3*x1 - p4*x2 ;
```

INITIAL CONDITIONS

```
model.sym.x0 = sym(zeros(size(model.sym.x)));

model.sym.x0(1) = 0;
model.sym.x0(2) = 0;
```

OBSERVALES

```
model.sym.y = sym(zeros(1,1));

model.sym.y(1) = x2;


end


ans =
      sym: [1x1 struct]
    param: 'log10'
```

3.7.2 Simulation

```
function example_dirac_secondorder_vectmult()
```

COMPILATION

```
    [exdir,~,~]=fileparts(which('example_dirac_secondorder_vectmult.m'));
    % compile the model
    amiwrap('model_dirac_secondorder_vectmult','model_dirac_secondorder_vectmult_syms',exdir,2)


Generating model struct ...
x | k | p | deltax | xdot | deltaxdot | ddeltaxdx | ddeltaxdp | ddeltaxdt | root | drootdx | sx | drootdp | drootdt | dtaudp
z |

Generating C code ...
deltasx | deltax | dsigma_ydp | dsigma_zdp | dydp | dzdp | root | sigma_y | sigma_z | stau | xdot | y | z | headers | wrapfunc
headers | wrapfunctions |
Compiling mex file ...
amici | Building with 'Xcode with Clang'.
MEX completed successfully.
Building with 'Xcode with Clang'.
MEX completed successfully.
amici | Building with 'Xcode with Clang'.
MEX completed successfully.
Building with 'Xcode with Clang'.
MEX completed successfully.
```

SIMULATION

```
    % time vector
    t = linspace(0,3,1001);
    p = [1;0.5;2;3];
    k = [];
    v = [0.7;0.3;1.4;0.1];

    options = amioption('sensi',0,...
```

```
    'maxsteps',1e4);

% load mex into memory
[msg] = which('model_dirac_secondorder_vectmult'); % fix for inaccessability problems
options.sensi = 2;
sol = simulate_model_dirac_secondorder_vectmult(t,log10(p),k,[],options,v);
```

FORWARD SENSITIVITY ANALYSIS

```
options.sensi = 2;

sol = simulate_model_dirac_secondorder_vectmult(t,log10(p),k,[],options,v);
```

FINITE DIFFERENCES

```
options.sensi = 1;

eps = 1e-4;
xi = log10(p);
for ip = 1:4;
    xip = xi;
    xip(ip) = xip(ip) + eps;
    solp = simulate_model_dirac_secondorder_vectmult(t,xip,k,[],options);
    s2x_fd(:,:,ip) = sum(bsxfun(@times,(solp.sx - sol.sx)/eps,permute(v,[3,2,1])),3);
    s2y_fd(:,:,ip) = sum(bsxfun(@times,(solp.sy - sol.sy)/eps,permute(v,[3,2,1])),3);
end
```

PLOTTING

```
figure
c_x = get(gca,'ColorOrder');
for ip = 1:4
    subplot(4,2,ip*2-1)
    hold on
    for ix = 1:size(sol.x,2)
        plot(t,sol.s2x(:,ix,ip),'.-','Color',c_x(ix,:))
        plot(t,s2x_fd(:,ix,ip),'--','Color',c_x(ix,:))
    end
    ylim([-10,10])
    legend('x1','x1_fd','x2','x2_fd','Location','NorthEastOutside')
    legend boxoff
    title(['state sensitivity for p' num2str(ip)])
    xlabel('time t')
    ylabel('x')
    box on

    subplot(4,2,ip*2)
    plot(t,abs(sol.s2x(:,:,ip)-s2x_fd(:,:,ip)),'r--')
    legend('error x1','error x2','Location','NorthEastOutside')
    legend boxoff
    title(['state sensitivity for p' num2str(ip)])
    xlabel('time t')
    ylabel('error')
    ylim([1e-12,1e0])
    set(gca,'YScale','log')
    box on
end
set(gcf,'Position',[100 300 1200 500])

figure
for ip = 1:4
    subplot(4,2,ip*2-1)
    hold on
    for iy = 1:size(sol.y,2)
        plot(t,sol.s2y(:,iy,ip),'.-','Color',c_x(iy,:))
        plot(t,s2y_fd(:,iy,ip),'--','Color',c_x(iy,:))
    end
    ylim([-10,10])
    legend('y1','y1_fd','Location','NorthEastOutside')
    legend boxoff
    title(['observable sensitivity for p' num2str(ip)])
    xlabel('time t')
    ylabel('y')
    box on

    subplot(4,2,ip*2)
    plot(t,abs(sol.s2y(:,:,ip)-s2y_fd(:,:,ip)),'r--')
```

```
        legend('error y1','Location','NorthEastOutside')
        legend boxoff
        title(['observable sensitivity for p' num2str(ip)])
        xlabel('time t')
        ylabel('error')
        ylim([1e-12,1e0])
        set(gca,'YScale','log')
        box on
    end
    set(gcf,'Position',[100 300 1200 500])

    drawnow
```

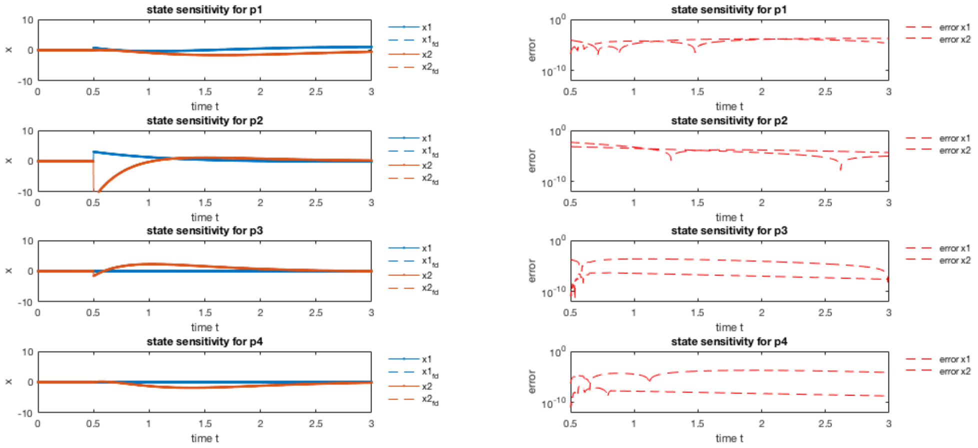

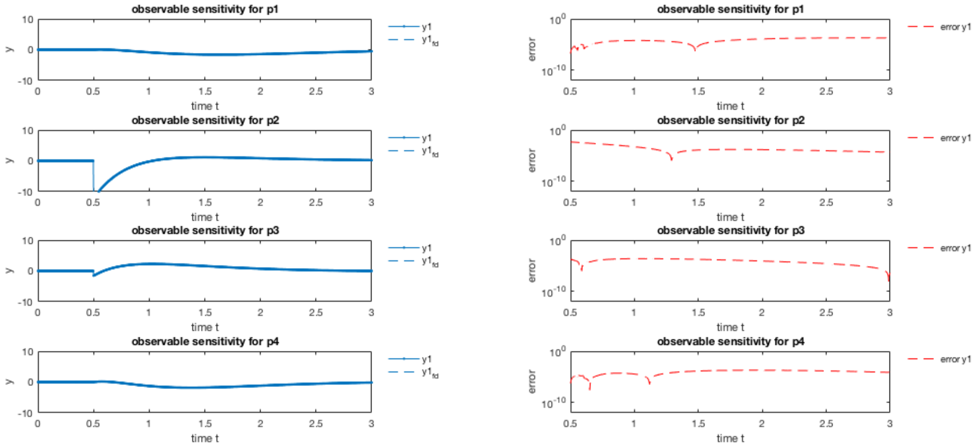


```
end
```

3.8 Example Adjoint

3.8.1 Model Definition

```
function [model] = model_adjoint_syms()
```

STATES

```
% create state syms
syms x1

% create state vector
model.sym.x = [x1];
```

PARAMETERS (for these sensitivities will be computed)

```
% create parameter syms
syms p1 p2 p3

% create parameter vector
model.sym.p = [p1 p2 p3];


% set the parametrisation of the problem options are 'log', 'log10' and
% 'lin' (default).
model.param = 'log10';
```

SYSTEM EQUATIONS

```
% create symbolic variable for time
syms t

model.sym.xdot = sym(zeros(size(model.sym.x)));

% piecewise defined function
model.sym.xdot(1) = -p1*x1*heaviside(t-2) + p2;
```

INITIAL CONDITIONS

```
model.sym.x0 = sym(zeros(size(model.sym.x)));

model.sym.x0(1) = p3;
```

OBSERVALES

```
model.sym.y = sym(zeros(1,1));

model.sym.y(1) = x1;


end


ans =
      sym: [1x1 struct]
    param: 'log10'
```

3.8.2 Simulation

```
function example_adjoint()
```

COMPILATION

```
    [exdir,~,~]=fileparts(which('example_adjoint.m'));
    % compile the model
    amiwrap('model_adjoint','model_adjoint_syms',exdir)


Generating model struct ...
Parsing model struct ...

Generating C code ...
headers | wrapfunctions |
Compiling mex file ...
amici | Building with 'Xcode with Clang'.
MEX completed successfully.
Building with 'Xcode with Clang'.
MEX completed successfully.
```

SIMULATION

```
% time vector
t = [linspace(0,4,5)];
p = [1.1,0.3,1];
k = [];

D.Y = [     1.0171
    1.3423
    1.6585
    0.9814
    0.3288];

D.Sigma_Y = 0.1*ones(size(D.Y));


options.sensi = 1;
options.sensi_meth = 'adjoint';
options.maxsteps = 1e4;
options.rtol = 1e-12;
options.atol = 1e-12;
% load mex into memory
[~] = which('simulate_model_adjoint'); % fix for inaccessability problems
sol = simulate_model_adjoint(t,log10(p),k,D,options);
```

Plot

```
figure
subplot(3,1,1)
errorbar(t,D.Y,D.Sigma_Y)
hold on
% plot(t,sol.y)

xlabel('time t')
ylabel('observable')
title(['log-likelihood: ' num2str(sol.llh) ])

y = (p(2)*t + p(3)).*(t<2) + ( (2*p(2)+p(3)-p(2)/p(1))*exp(-p(1)*(t-2))+p(2)/p(1) ).*(t>=2);


tfine = linspace(0,4,100001);
xfine = (p(2)*tfine + 1).*(tfine<2) + ( (2*p(2)+p(3)-p(2)/p(1))*exp(-p(1)*(tfine-2))+p(2)/p(1) ).*(tfine>=2);

mu = zeros(1,length(tfine));
for it = 1:length(t)
    if(t(it)<=2)
        mu = mu + ((y(it)-D.Y(it))/(D.Sigma_Y(it)^2))*(tfine<=t(it));
    else
        mu = mu + ((y(it)-D.Y(it))/(D.Sigma_Y(it)^2))*exp(p(1)*(tfine-t(it))).*(tfine<=t(it)).*(tfine>2) + ((y(it)-D.Y(it)
    end
end
plot(tfine,xfine)
legend('data','simulation')
xlim([min(t)-0.5,max(t)+0.5])
subplot(3,1,2)
plot(tfine,mu)
ylabel('adjoint')
xlabel('time t')
xlim([min(t)-0.5,max(t)+0.5])

subplot(3,1,3)

plot(fliplr(tfine),-cumsum(fliplr(-mu.*xfine.*(tfine>2)))*p(1)*log(10)*(t(end)/numel(tfine)))
hold on
plot(fliplr(tfine),-cumsum(fliplr(mu))*p(2)*log(10)*(t(end)/numel(tfine)))
plot(tfine,-mu(1)*p(3)*log(10)*(tfine<2))
xlim([min(t)-0.5,max(t)+0.5])
ylabel('integral')
xlabel('time t')

legend('p1','p2','p3')

grad(1,1) = -trapz(tfine,-mu.*xfine.*(tfine>2))*p(1)*log(10);
grad(2,1) = -trapz(tfine,mu)*p(2)*log(10);
grad(3,1) = -mu(1)*p(3)*log(10);

plot(zeros(3,1),grad,'ko')
```

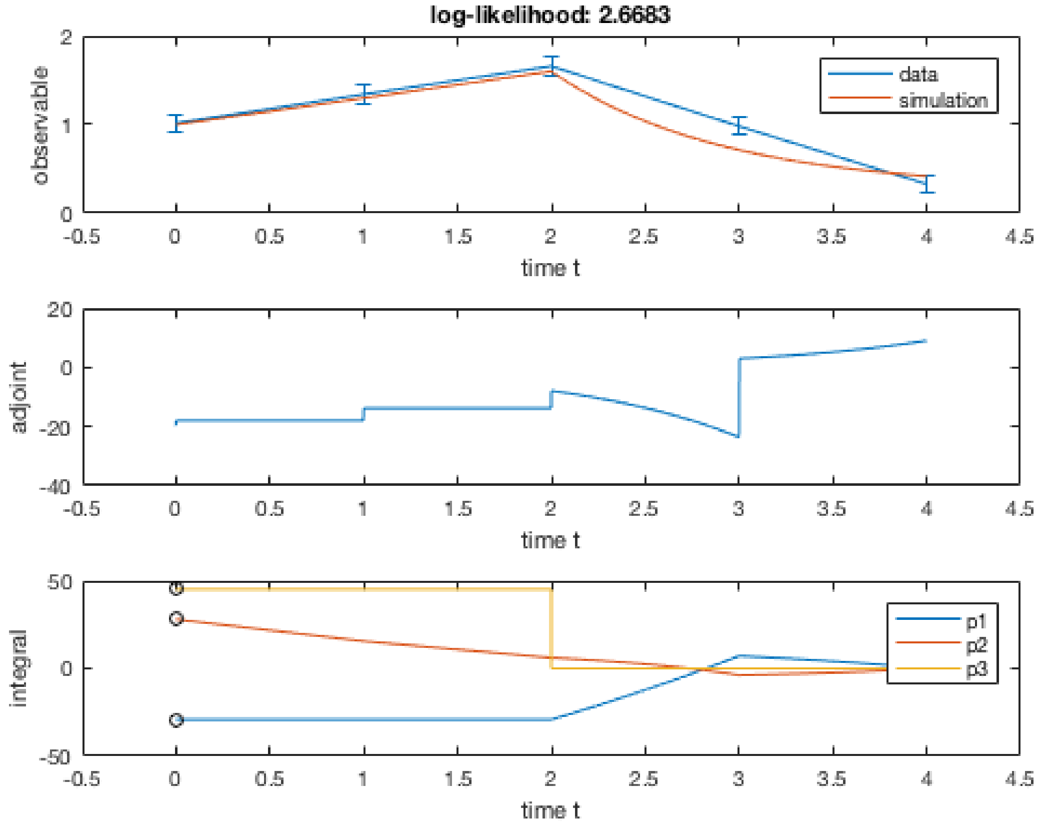

FD

```
eps = 1e-5;
xi = log10(p);
grad_fd_f = NaN(3,1);
grad_fd_b = NaN(3,1);
for ip = 1:3;
    options.sensi = 0;
    xip = xi;
    xip(ip) = xip(ip) + eps;
    solp = simulate_model_adjoint(t,xip,k,D,options);
    grad_fd_f(ip,1) = (solp.llh-sol.llh)/eps;
    xip = xi;
    xip(ip) = xip(ip) - eps;
    solp = simulate_model_adjoint(t,xip,k,D,options);
    grad_fd_b(ip,1) = -(solp.llh-sol.llh)/eps;
end

figure
plot(abs(grad),abs(grad_fd_f),'o')
hold on
plot(abs(grad),abs(grad_fd_b),'o')
plot(abs(grad),mean([abs(grad_fd_b),abs(grad_fd_f)],2),'o')
plot(abs(grad),abs(sol.sllh),'o')
plot([1e1,1e2],[1e1,1e2],'k:')
set(gca,'XScale','log')
set(gca,'YScale','log')
axis square
legend('forward FD','backward FD','central FD','adjoint sensintivity analysis','Location','SouthEast')
xlabel('analytic absolute value of gradient element')
ylabel('computed absolute value of gradient element')
set(gcf,'Position',[100 300 1200 500])

drawnow
```

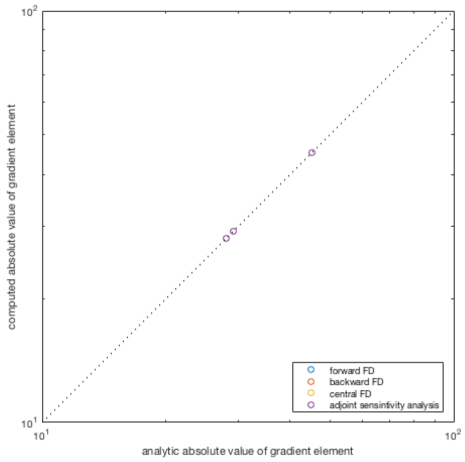


```
end
```

4 Code Organization

In the following we will briefly outline what happens when a model is compiled. For a more detailed description we refer the reader to the documentation of the individual functions.

After specifying a model (see Model Definition) the user will typically compile the model by invoking amiwrap(). amiwrap() first instantiates an object of the class amimodel. The properties of this object are initialised based on the user-defined model. If the o2flag is active, all subsequent computations will also be carried out on the augmented system, which also includes the equations for forward sensitivities. This allows the computation of second order sensitivities in a forward-forward approach. A forward-adjoint approach will be implemented in the future.

The fun fields of this object will then be populated by amimodel::parseModel(). The amimodel::fun field contains all function definitions of type amifun which are required for model compilation. The set of functions to be considered will depend on the user specification of the model fields amimodel::adjoint and amimodel::forward (see Options) as well as the employed solver (CVODES or IDAS, see Differential Equation). For all considered functions amimodel::parseModel() will check their dependencies via amimodel::checkDeps(). These dependencies are a subset of the user-specified fields of amimodel::fun (see Attach to Model Struct). amimodel::parseModel() compares the hashes of all dependencies against the amimodel::HTable of possible previous compilations and will only compute necessary symbolic expressions if changes in these fields occured.

For all functions for which amimodel::fun exists, amimodel::generateC() will generate C files. These files together with their respective header files will be placed in $AMICIDIR/models/*modelname*. amimodel::generateC() will also generate wrapfunctions.h and wrapfunctions.c. These files define and declare model unspecific wrapper functions around model specific functions. This construction allows us to use to build multiple different models against the same simulation routines by linking different realisations of these wrapper functions.

All the generated C functions are subsequently compiled by amimodel::compileC(). For all functions individual object files are created to reduce the computation cost of code optimization. Moreover necessary code from sundials and SuiteSparse is compiled as object files and placed in /models/*mexext*, where mexext stands for the string returned by matlab to the command mexext. The mex simulation file is compiled from amiwrap.c, linked against all object necessary of sundials, SuiteSparse and model specific functions. Depending on the required solver, the compilation will either include cvodewrap.h or idawrap.h. These files implement solver specific realisations of the AMI... functions used in amiwrap.c and amici.c. This allows the use of the same simulation routines for both CVODES and IDAS.

5 Hierarchical Index

5.1 Class Hierarchy

This inheritance list is sorted roughly, but not completely, alphabetically:

amievent 44

amifun 46

ExpData 70

handle

- **amidata** 41
- **amimodel** 51
- **SBMLode** 75

SetGet

- **amioption** 65

modelTest 71

ReturnData 72

sym

- **optsym** 71

TempData 80

UserData 87

6 Class Index

6.1 Class List

Here are the classes, structs, unions and interfaces with brief descriptions:

amidata
AMIDATA provides a data container to pass experimental data to the simulation routine for likelihood computation 41

amievent
AMIEVENT defines events which later on will be transformed into appropriate C code 44

amifun
AMIFUN defines functions which later on will be transformed into appropriate C code 46

amimodel
AMIMODEL carries all model definitions including functions and events 51

amioption
AMIOPTION provides an option container to pass simulation parameters to the simulation routine 65

ExpData
Struct that carries all information about experimental data 70

modelTest
MODELTEST Summary of this class goes here Detailed explanation goes here 71

optsym
OPTSYM is an auxiliary class to gain access to the private symbolic property s which is necessary to be able to call symobj::optimize on it 71

ReturnData
Struct that stores all data which is later returned by the mex function 72

SBMLode
SBMLMODEL provides an intermediate container between the SBML definition and an amimodel object 75

TempData
Struct that provides temporary storage for different variables 80

UserData
Struct that stores all user provided data 87

7 Class Documentation

7.1 amidata Class Reference

AMIDATA provides a data container to pass experimental data to the simulation routine for likelihood computation.

Inheritance diagram for amidata:

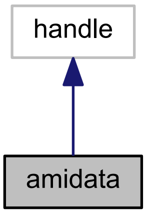

Collaboration diagram for amidata:

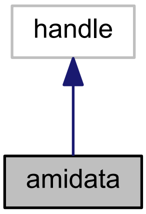

Public Member Functions

- amidata (matlabtypesubstitute varargin)
  initialisation via struct

Public Attributes

- matlabtypesubstitute nt = 0
  number of timepoints
- matlabtypesubstitute ny = 0
  number of observables
- matlabtypesubstitute nz = 0
  number of event observables
- matlabtypesubstitute ne = 0
  number of events
- matlabtypesubstitute nk = 0
  number of conditions/constants
- matlabtypesubstitute t = double.empty("")
  timepoints of observations
- matlabtypesubstitute Y = double.empty("")
  observations
- matlabtypesubstitute Sigma_Y = double.empty("")
  standard deviation of observations
- matlabtypesubstitute Z = double.empty("")
  event observations
- matlabtypesubstitute Sigma_Z = double.empty("")
  standard deviation of event observations
- matlabtypesubstitute condition = double.empty("")
  experimental condition

7.1.1 Detailed Description

Definition at line 17 of file amidata.m.

7.1.2 Member Data Documentation

7.1.2.1 nt = 0

Default: 0

Note

This property has custom functionality when its value is changed.

Definition at line 28 of file amidata.m.

7.1.2.2 ny = 0

Default: 0

Note

This property has custom functionality when its value is changed.

Definition at line 36 of file amidata.m.

7.1.2.3 nz = 0

Default: 0

Note

This property has custom functionality when its value is changed.

Definition at line 44 of file amidata.m.

7.1.2.4 ne = 0

Default: 0

Note

This property has custom functionality when its value is changed.

Definition at line 52 of file amidata.m.

7.1.2.5 nk = 0

Default: 0

Note

This property has custom functionality when its value is changed.

Definition at line 60 of file amidata.m.

7.1.2.6 t = double.empty("")

Default: double.empty("")

Note

This property has custom functionality when its value is changed.

Definition at line 68 of file amidata.m.

7.1.2.7 Y = double.empty("")

Default: double.empty("")

Note

This property has custom functionality when its value is changed.

Definition at line 76 of file amidata.m.

7.1.2.8 Sigma_Y = double.empty("")

Default: double.empty("")

Note

This property has custom functionality when its value is changed.

Definition at line 84 of file amidata.m.

7.1.2.9 Z = double.empty("")

Default: double.empty("")

Note

This property has custom functionality when its value is changed.

Definition at line 92 of file amidata.m.

7.1.2.10 Sigma_Z = double.empty("")

Default: double.empty("")

Note

This property has custom functionality when its value is changed.

Definition at line 100 of file amidata.m.

7.1.2.11 condition = double.empty("")

Default: double.empty("")

Note

This property has custom functionality when its value is changed.

Definition at line 108 of file amidata.m.

7.2 amievent Class Reference

AMIEVENT defines events which later on will be transformed into appropriate C code.

Public Member Functions

- amievent (matlabtypesubstitute trigger, matlabtypesubstitute bolus, matlabtypesubstitute z)

  amievent constructs an amievent object from the provided input.
- mlhsInnerSubst$<$ matlabtypesubstitute $>$ setHflag (::double hflag)

  gethflag sets the hflag property.

Public Attributes

- ::symbolic trigger = sym.empty("")

  the trigger function activates the event on every zero crossing
- ::symbolic bolus = sym.empty("")

  the bolus function defines the change in states that is applied on every event occurence
- ::symbolic z = sym.empty("")

  output function for the event
- matlabtypesubstitute hflag = logical.empty("")

  flag indicating that a heaviside function is present, this helps to speed up symbolic computations

7.2.1 Detailed Description

Definition at line 17 of file amievent.m.

7.2.2 Constructor & Destructor Documentation

7.2.2.1 amievent (matlabtypesubstitute *trigger*, matlabtypesubstitute *bolus*, matlabtypesubstitute *z*)

Parameters

trigger	trigger function, the event will be triggered on at all roots of this function
bolus	the bolus that will be added to all states on every occurence of the event
z	the event output that will be reported on every occurence of the event

Definition at line 75 of file amievent.m.

7.2.3 Member Function Documentation

7.2.3.1 mlhsInnerSubst<::amievent > setHflag (::double *hflag*)

Parameters

hflag	value for the hflag property

Return values

this	updated event definition object

Definition at line 18 of file setHflag.m.

Here is the caller graph for this function:

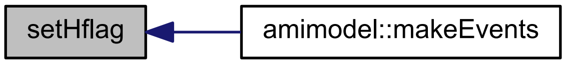

7.2.4 Member Data Documentation

7.2.4.1 trigger = sym.empty("")

Note

This property has non-standard access specifiers: `SetAccess = Private, GetAccess = Public`
`Matlab documentation of property attributes.`
Default: sym.empty("")

Definition at line 27 of file amievent.m.

7.2.4.2 bolus = sym.empty("")

Note

This property has non-standard access specifiers: `SetAccess = Private, GetAccess = Public`
`Matlab documentation of property attributes.`
Default: sym.empty("")

Definition at line 38 of file amievent.m.

7.2.4.3 z = sym.empty("")

Note

This property has non-standard access specifiers: `SetAccess = Private, GetAccess = Public`
`Matlab documentation of property attributes.`
Default: sym.empty("")

Definition at line 49 of file amievent.m.

7.2.4.4 hflag = logical.empty("")

Note

This property has non-standard access specifiers: `SetAccess = Private, GetAccess = Public`
`Matlab documentation of property attributes.`
Default: logical.empty("")

Definition at line 60 of file amievent.m.

7.3 amifun Class Reference

AMIFUN defines functions which later on will be transformed into appropriate C code.

Public Member Functions

- amifun (matlabtypesubstitute funstr, matlabtypesubstitute model)
  amievent constructs an amifun object from the provided input.
- noret::substitute printLocalVars (::amimodel model,::fileid fid)
  printlocalvars prints the C code for the initialisation of local variables into the file specified by fid.
- noret::substitute writeCcode_sensi (::amimodel model,::fileid fid)
  writeCcode_sensi is a wrapper for writeCcode which loops over parameters and reduces overhead by check nonzero values
- noret::substitute writeCcode (::amimodel model,::fileid fid)
  writeCcode is a wrapper for gccode which initialises data and reduces overhead by check nonzero values
- noret::substitute gccode (::amimodel model,::fileid fid)
  gccode transforms symbolic expressions into c code and writes the respective expression into a specified file
- mlhsInnerSubst< matlabtypesubstitute > getDeps (::amimodel model)
  getDeps populates the sensiflag for the requested function
- mlhsInnerSubst< matlabtypesubstitute > getArgs (::amimodel model)
  getFArgs populates the fargstr property with the argument string of the respective model function (if applicable). model functions are not wrapped versions of functions which have a model specific name and for which the call is solver specific.
- mlhsInnerSubst< matlabtypesubstitute > getNVecs ()
  getfunargs populates the nvecs property with the names of the N_Vector elements which are required in the execution of the function (if applicable). the information is directly extracted from the argument string

- mlhsInnerSubst< matlabtypesubstitute > getCVar ()

  getCVar populates the cvar property
- mlhsInnerSubst< matlabtypesubstitute > getSensiFlag ()

  getSensiFlag populates the sensiflag property

Public Attributes

- ::symbolic sym = sym.empty("")

  symbolic definition struct
- ::symbolic strsym = sym.empty("")

  short symbolic string which can be used for the reuse of precomputed values
- ::symbolic strsym_old = sym.empty("")

  short symbolic string which can be used for the reuse of old values
- ::char funstr = char.empty("")

  name of the model
- ::char cvar = char.empty("")

  name of the c variable
- ::char argstr = char.empty("")

  argument string (solver specific)
- ::char fargstr = char.empty("")

  argument string (solver unspecific)
- ::cell deps = cell.empty("")

  dependencies on other functions
- matlabtypesubstitute nvecs = cell.empty("")

  nvec dependencies
- matlabtypesubstitute sensiflag = logical.empty("")

  indicates whether the function is a sensitivity or derivative with respect to parameters

7.3.1 Detailed Description

Definition at line 17 of file amifun.m.

7.3.2 Constructor & Destructor Documentation

7.3.2.1 amifun (matlabtypesubstitute *funstr,* matlabtypesubstitute *model*)

Parameters

funstr	name of the requested function
model	amimodel object which carries all symbolic definitions to construct the funtion

Definition at line 111 of file amifun.m.

7.3.3 Member Function Documentation

7.3.3.1 noret::substitute printLocalVars (::amimodel *model,* ::fileid *fid*)

Parameters

model	this struct must contain all necessary symbolic definitions
fid	file id in which the final expression is written

Return values

fid	Nothing

Definition at line 18 of file printLocalVars.m.

7.3.3.2 noret::substitute writeCcode_sensi (::amimodel *model,* ::fileid *fid*)

Parameters

model	model defintion object
fid	file id in which the final expression is written

Return values

fid	void

Definition at line 18 of file writeCcode_sensi.m.

7.3.3.3 noret::substitute writeCcode (::amimodel *model,* ::fileid *fid*)

Parameters

model	model defintion object
fid	file id in which the final expression is written

Return values

fid	void

Definition at line 18 of file writeCcode.m.

Here is the call graph for this function:

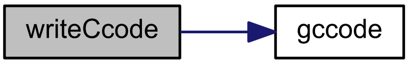

7.3.3.4 mlhsInnerSubst<::amifun > gccode (::amimodel *model,* ::fileid *fid*)

Parameters

model	model defintion object
fid	file id in which the expression should be written

Return values

this	function definition object

Definition at line 18 of file gccode.m.

Here is the caller graph for this function:

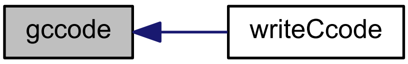

7.3.3.5 mlhsInnerSubst<::amifun > getDeps (::amimodel *model*)

Parameters

model	model definition object

Return values

this	updated function definition object

Definition at line 18 of file getDeps.m.

7.3.3.6 mlhsInnerSubst<::amifun > getArgs (::amimodel *model*)

Parameters

model	model definition object

Return values

this	updated function definition object

Definition at line 18 of file getArgs.m.

7.3.3.7 mlhsInnerSubst<::amifun > getNVecs ( )

Return values

this	updated function definition object

Definition at line 18 of file getNVecs.m.

7.3.3.8 mlhsInnerSubst<::amifun > getCVar ( )

Return values

this	updated function definition object

Definition at line 18 of file getCVar.m.

7.3.3.9 mlhsInnerSubst<::amifun > getSensiFlag ( )

Return values

this	updated function definition object

Definition at line 18 of file getSensiFlag.m.

7.3.4 Member Data Documentation

7.3.4.1 sym = sym.empty("")

Default: sym.empty("")

Definition at line 27 of file amifun.m.

7.3.4.2 strsym = sym.empty("")

Default: sym.empty("")

Definition at line 35 of file amifun.m.

7.3.4.3 strsym_old = sym.empty("")

Default: sym.empty("")

Definition at line 43 of file amifun.m.

7.3.4.4 funstr = char.empty("")

Default: char.empty("")

Definition at line 51 of file amifun.m.

7.3.4.5 cvar = char.empty("")

Default: char.empty("")

Definition at line 59 of file amifun.m.

7.3.4.6 argstr = char.empty("")

Default: char.empty("")

Definition at line 67 of file amifun.m.

7.3.4.7 fargstr = char.empty("")

Default: char.empty("")

Definition at line 75 of file amifun.m.

7.3.4.8 deps = cell.empty("")

Default: cell.empty("")

Definition at line 83 of file amifun.m.

7.3.4.9 nvecs = cell.empty("")

Default: cell.empty("")

Definition at line 91 of file amifun.m.

7.3.4.10 sensiflag = logical.empty("")

Default: logical.empty("")

Definition at line 99 of file amifun.m.

7.4 amimodel Class Reference

AMIMODEL carries all model definitions including functions and events.

Inheritance diagram for amimodel:

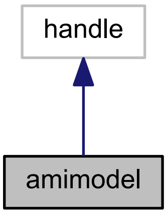

Collaboration diagram for amimodel:

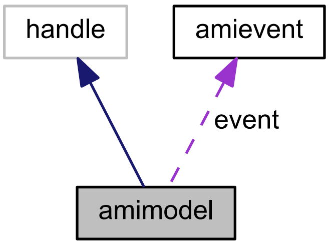

Public Member Functions

- amimodel (::string symfun,::string modelname)

  amimodel initializes the model object based on the provided symfun and modelname
- noret::substitute updateRHS (matlabtypesubstitute xdot)

  updateRHS updates the private fun property .fun.xdot.sym (right hand side of the differential equation)
- noret::substitute parseModel ()

  parseModel parses the model definition and computes all necessary symbolic expressions.
- noret::substitute generateC ()

  generateC generates the c files which will be used in the compilation.

- noret::substitute compileC ()

  compileC compiles the mex simulation file
- noret::substitute generateM (::amimodel amimodelo2)

  generateM generates the matlab wrapper for the compiled C files.
- noret::substitute getFun (::struct HTable,::string funstr)

  getFun generates symbolic expressions for the requested function.
- noret::substitute makeEvents ()

  makeEvents extracts discontiniuties from the model right hand side and converts them into events
- noret::substitute makeSyms ()

  makeSyms extracts symbolic definition from the user provided model and checks them for consistency
- mlhsInnerSubst< matlabtypesubstitute > checkDeps (::struct HTable,::cell deps)

  checkDeps checks the dependencies of functions and populates sym fields if necessary
- mlhsInnerSubst< matlabtypesubstitute > loadOldHashes ()

  loadOldHashes loads information from a previous compilation of the model.
- mlhsInnerSubst< matlabtypesubstitute > augmento2 ()

  augmento2 augments the system equation to also include equations for sensitivity equation. This will enable us to compute second order sensitivities in a forward-adjoint or forward-forward apporach later on.
- mlhsInnerSubst< matlabtypesubstitute > augmento2vec ()

  augmento2 augments the system equation to also include equations for sensitivity equation. This will enable us to compute second order sensitivities in a forward-adjoint or forward-forward apporach later on.

Public Attributes

- ::struct sym = struct.empty("")

  symbolic definition struct
- ::struct fun = struct.empty("")

  struct which stores information for which functions c code needs to be generated
- ::amievent event = amievent.empty("")

  struct which stores information for which functions c code needs to be generated
- ::string modelname = char.empty("")

  name of the model
- ::struct HTable = struct.empty("")

  struct that contains hash values for the symbolic model definitions
- ::bool debug = false

  flag indicating whether debugging symbols should be compiled
- ::bool adjoint = true

  flag indicating whether adjoint sensitivities should be enabled
- ::bool forward = true

  flag indicating whether forward sensitivities should be enabled
- ::double t0 = 0

  default initial time
- ::string wtype = char.empty("")

  type of wrapper (cvodes/idas)
- ::int nx = double.empty("")

  number of states
- ::int nxtrue = 0

  number of original states for second order sensitivities
- ::int ny = double.empty("")

  number of observables
- ::int nytrue = 0

  number of original observables for second order sensitivities

- ::int np = double.empty("")

  number of parameters
- ::int nk = double.empty("")

  number of constants
- ::int nevent = double.empty("")

  number of events
- ::int nz = double.empty("")

  number of event outputs
- ::int nztrue = double.empty("")

  number of original event outputs for second order sensitivities
- ::∗int id = double.empty("")

  flag for DAEs
- ::int ubw = double.empty("")

  upper Jacobian bandwidth
- ::int lbw = double.empty("")

  lower Jacobian bandwidth
- ::int nnz = double.empty("")

  number of nonzero entries in Jacobian
- ::∗int sparseidx = double.empty("")

  dataindexes of sparse Jacobian
- ::∗int rowvals = double.empty("")

  rowindexes of sparse Jacobian
- ::∗int colptrs = double.empty("")

  columnindexes of sparse Jacobian
- ::∗int sparseidxB = double.empty("")

  dataindexes of sparse Jacobian
- ::∗int rowvalsB = double.empty("")

  rowindexes of sparse Jacobian
- ::∗int colptrsB = double.empty("")

  columnindexes of sparse Jacobian
- ::∗cell funs = cell.empty("")

  cell array of functions to be compiled
- ::string coptim = "-O3"

  optimisation flag for compilation
- ::string param = "lin"

  default parametrisation
- matlabtypesubstitute wrap_path = char.empty("")

  path to wrapper
- matlabtypesubstitute recompile = false

  flag to enforce recompilation of the model
- matlabtypesubstitute cfun = struct.empty("")

  storage for flags determining recompilation of individual functions
- matlabtypesubstitute o2flag = 0

  flag which identifies augmented models 0 indicates no augmentation 1 indicates augmentation by first order sensitivities (yields second order sensitivities) 2 indicates augmentation by one linear combination of first order sensitivities (yields hessian-vector product)
- matlabtypesubstitute compver = 9

  counter that allows enforcing of recompilation of models after code changes
- matlabtypesubstitute z2event = double.empty("")

  vector that maps outputs to events
- matlabtypesubstitute splineflag = false

flag indicating whether the model contains spline functions

- matlabtypesubstitute minflag = false

  flag indicating whether the model contains min functions

- matlabtypesubstitute maxflag = false

  flag indicating whether the model contains max functions

- ::int nw = 0

  number of derived variables w, w is used for code optimization to reduce the number of frequently occuring expressions

- ::int ndwdx = 0

  number of derivatives of derived variables w, dwdx

- ::int ndwdp = 0

  number of derivatives of derived variables w, dwdp

7.4.1 Detailed Description

Definition at line 17 of file amimodel.m.

7.4.2 Constructor & Destructor Documentation

7.4.2.1 amimodel (::string *symfun*, ::string *modelname*)

Parameters

symfun	this is the string to the function which generates the modelstruct. You can also directly pass the struct here
modelname	name of the model

Definition at line 506 of file amimodel.m.

Here is the caller graph for this function:

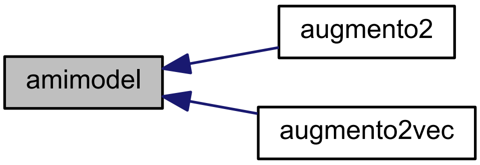

7.4.3 Member Function Documentation

7.4.3.1 noret::substitute updateRHS (matlabtypesubstitute *xdot*)

Parameters

xdot	new right hand side of the differential equation

Definition at line 580 of file amimodel.m.

7.4.3.2 noret::substitute generateC ( )

Return values

this	model definition object

Definition at line 18 of file generateC.m.

7.4.3.3 noret::substitute compileC ( )

Return values

this	model definition object

Definition at line 18 of file compileC.m.

7.4.3.4 noret::substitute generateM (::amimodel *amimodelo2*)

Parameters

amimodelo2	this struct must contain all necessary symbolic definitions for second order sensivities

Return values

this	model definition object

Definition at line 18 of file generateM.m.

7.4.3.5 noret::substitute getFun (::struct *HTable,* ::string *funstr*)

Parameters

HTable	struct with hashes of symbolic definition from the previous compilation
funstr	function for which symbolic expressions should be computed

Definition at line 18 of file getFun.m.

Here is the call graph for this function:

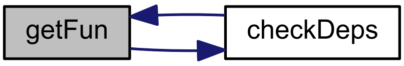

Here is the caller graph for this function:

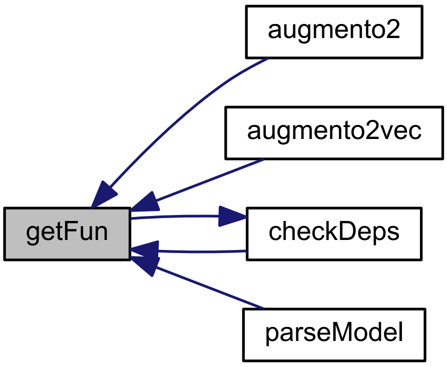

7.4.3.6 mlhsInnerSubst<::bool > checkDeps (::struct *HTable*, ::cell *deps*)

Parameters

HTable	struct with reference hashes of functions in its fields
deps	cell array with containing a list of dependencies

Return values

cflag	boolean indicating whether any of the dependencies have changed with respect to the hashes stored in HTable

Definition at line 18 of file checkDeps.m.

Here is the call graph for this function:

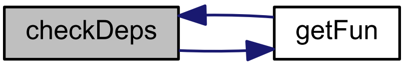

Here is the caller graph for this function:

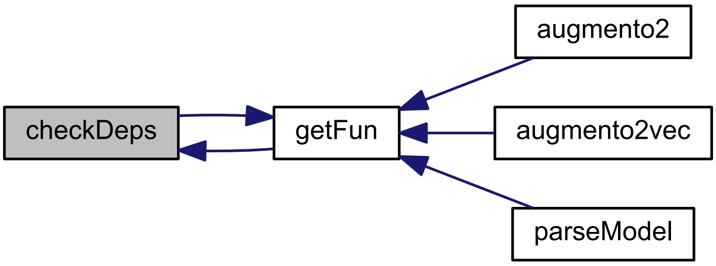

7.4.3.7 mlhsInnerSubst<::struct > loadOldHashes ( )

Return values

HTable	struct with hashes of symbolic definition from the previous compilation

Definition at line 18 of file loadOldHashes.m.

Here is the caller graph for this function:

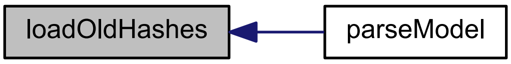

7.4.3.8 mlhsInnerSubst< matlabtypesubstitute > augmento2 ( )

Return values

this	augmented system which contains symbolic definition of the original system and its sensitivities

Definition at line 18 of file augmento2.m.

Here is the call graph for this function:

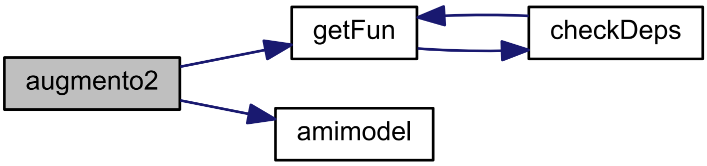

7.4.3.9 mlhsInnerSubst< matlabtypesubstitute > augmento2vec ( )

Return values

this	augmented system which contains symbolic definition of the original system and its sensitivities

Definition at line 18 of file augmento2vec.m.

Here is the call graph for this function:

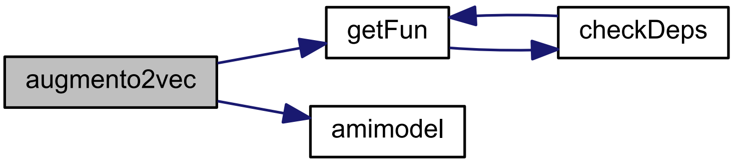

7.4.4 Member Data Documentation

7.4.4.1 sym = struct.empty("")

Note

This property has non-standard access specifiers: `SetAccess = Private, GetAccess = Public`
`Matlab documentation of property attributes.`
Default: struct.empty("")

Definition at line 27 of file amimodel.m.

7.4.4.2 fun = struct.empty("")

Note

This property has non-standard access specifiers: `SetAccess = Private, GetAccess = Public`
`Matlab documentation of property attributes.`
Default: struct.empty("")

Definition at line 38 of file amimodel.m.

7.4.4.3 event = amievent.empty("")

Note

This property has non-standard access specifiers: `SetAccess = Private, GetAccess = Public`
`Matlab documentation of property attributes.`
Default: amievent.empty("")

Definition at line 49 of file amimodel.m.

7.4.4.4 modelname = char.empty("")

Note

This property has non-standard access specifiers: `SetAccess = Private, GetAccess = Public`
`Matlab documentation of property attributes.`
Default: char.empty("")

Definition at line 61 of file amimodel.m.

7.4.4.5 HTable = struct.empty("")

Note

This property has non-standard access specifiers: `SetAccess = Private, GetAccess = Public`
`Matlab documentation of property attributes.`
Default: struct.empty("")

Definition at line 72 of file amimodel.m.

7.4.4.6 debug = false

Note

This property has non-standard access specifiers: `SetAccess = Private, GetAccess = Public`
`Matlab documentation of property attributes.`
Default: false

Definition at line 83 of file amimodel.m.

7.4.4.7 adjoint = true

Note

This property has non-standard access specifiers: `SetAccess = Private, GetAccess = Public`
`Matlab documentation of property attributes.`
Default: true

Definition at line 94 of file amimodel.m.

7.4.4.8 forward = true

Note

This property has non-standard access specifiers: `SetAccess = Private, GetAccess = Public`
`Matlab documentation of property attributes.`
Default: true

Definition at line 105 of file amimodel.m.

7.4.4.9 t0 = 0

Note

This property has non-standard access specifiers: `SetAccess = Private, GetAccess = Public`
`Matlab documentation of property attributes.`
Default: 0

Definition at line 116 of file amimodel.m.

7.4.4.10 wtype = char.empty("")

Note

This property has non-standard access specifiers: `SetAccess = Private, GetAccess = Public`
`Matlab documentation of property attributes.`
Default: char.empty("")

Definition at line 127 of file amimodel.m.

7.4.4.11 nx = double.empty("")

Note

This property has non-standard access specifiers: `SetAccess = Private, GetAccess = Public`
`Matlab documentation of property attributes.`
Default: double.empty("")

Definition at line 138 of file amimodel.m.

7.4.4.12 nxtrue = 0

Note

This property has non-standard access specifiers: `SetAccess = Private, GetAccess = Public`
`Matlab documentation of property attributes.`
Default: 0

Definition at line 149 of file amimodel.m.

7.4.4.13 ny = double.empty("")

Note

This property has non-standard access specifiers: `SetAccess = Private, GetAccess = Public`
`Matlab documentation of property attributes.`
Default: double.empty("")

Definition at line 160 of file amimodel.m.

7.4.4.14 nytrue = 0

Note

This property has non-standard access specifiers: `SetAccess = Private, GetAccess = Public`
`Matlab documentation of property attributes.`
Default: 0

Definition at line 171 of file amimodel.m.

7.4.4.15 np = double.empty("")

Note

This property has non-standard access specifiers: `SetAccess = Private, GetAccess = Public`
`Matlab documentation of property attributes.`
Default: double.empty("")

Definition at line 182 of file amimodel.m.

7.4.4.16 nk = double.empty("")

Note

This property has non-standard access specifiers: `SetAccess = Private, GetAccess = Public`
`Matlab documentation of property attributes.`
Default: double.empty("")

Definition at line 193 of file amimodel.m.

7.4.4.17 nevent = double.empty("")

Note

This property has non-standard access specifiers: `SetAccess = Private, GetAccess = Public`
`Matlab documentation of property attributes.`
Default: double.empty("")

Definition at line 204 of file amimodel.m.

7.4.4.18 nz = double.empty("")

Note

This property has non-standard access specifiers: `SetAccess = Private, GetAccess = Public`
`Matlab documentation of property attributes.`
Default: double.empty("")

Definition at line 215 of file amimodel.m.

7.4.4.19 nztrue = double.empty("")

Note

This property has non-standard access specifiers: `SetAccess = Private, GetAccess = Public`
`Matlab documentation of property attributes.`
Default: double.empty("")

Definition at line 226 of file amimodel.m.

7.4.4.20 id = double.empty("")

Note

This property has non-standard access specifiers: `SetAccess = Private, GetAccess = Public`
`Matlab documentation of property attributes.`
Default: double.empty("")

Definition at line 237 of file amimodel.m.

7.4.4.21 ubw = double.empty("")

Note

This property has non-standard access specifiers: `SetAccess = Private, GetAccess = Public`
`Matlab documentation of property attributes.`
Default: double.empty("")

Definition at line 248 of file amimodel.m.

7.4.4.22 lbw = double.empty("")

Note

This property has non-standard access specifiers: `SetAccess = Private, GetAccess = Public`
`Matlab documentation of property attributes.`
Default: double.empty("")

Definition at line 259 of file amimodel.m.

7.4.4.23 nnz = double.empty("")

Note

This property has non-standard access specifiers: `SetAccess = Private, GetAccess = Public`
`Matlab documentation of property attributes.`
Default: double.empty("")

Definition at line 270 of file amimodel.m.

7.4.4.24 sparseidx = double.empty("")

Note

This property has non-standard access specifiers: `SetAccess = Private, GetAccess = Public`
`Matlab documentation of property attributes.`
Default: double.empty("")

Definition at line 281 of file amimodel.m.

7.4.4.25 rowvals = double.empty("")

Note

This property has non-standard access specifiers: `SetAccess = Private, GetAccess = Public`
`Matlab documentation of property attributes.`
Default: double.empty("")

Definition at line 292 of file amimodel.m.

7.4.4.26 colptrs = double.empty("")

Note

This property has non-standard access specifiers: `SetAccess = Private, GetAccess = Public`
`Matlab documentation of property attributes.`
Default: double.empty("")

Definition at line 303 of file amimodel.m.

7.4.4.27 sparseidxB = double.empty("")

Note

This property has non-standard access specifiers: `SetAccess = Private, GetAccess = Public`
`Matlab documentation of property attributes.`
Default: double.empty("")

Definition at line 314 of file amimodel.m.

7.4.4.28 rowvalsB = double.empty("")

Note

This property has non-standard access specifiers: `SetAccess = Private, GetAccess = Public`
`Matlab documentation of property attributes.`
Default: double.empty("")

Definition at line 325 of file amimodel.m.

7.4.4.29 colptrsB = double.empty("")

Note

This property has non-standard access specifiers: `SetAccess = Private, GetAccess = Public`
`Matlab documentation of property attributes.`
Default: double.empty("")

Definition at line 336 of file amimodel.m.

7.4.4.30 funs = cell.empty("")

Note

This property has non-standard access specifiers: `SetAccess = Private, GetAccess = Public`
`Matlab documentation of property attributes.`
Default: cell.empty("")

Definition at line 347 of file amimodel.m.

7.4.4.31 coptim = "-O3"

Note

This property has non-standard access specifiers: `SetAccess = Private, GetAccess = Public`
`Matlab documentation of property attributes.`
Default: "-O3"

Definition at line 358 of file amimodel.m.

7.4.4.32 param = "lin"

Note

This property has non-standard access specifiers: `SetAccess = Private, GetAccess = Public`
`Matlab documentation of property attributes.`
Default: "lin"

Definition at line 369 of file amimodel.m.

7.4.4.33 wrap_path = char.empty("")

Note

This property has non-standard access specifiers: `SetAccess = Private, GetAccess = Public`
`Matlab documentation of property attributes.`
Default: char.empty("")

Definition at line 380 of file amimodel.m.

7.4.4.34 recompile = false

Note

This property has non-standard access specifiers: `SetAccess = Private, GetAccess = Public`
`Matlab documentation of property attributes.`
Default: false

Definition at line 391 of file amimodel.m.

7.4.4.35 cfun = struct.empty("")

Note

This property has non-standard access specifiers: `SetAccess = Private, GetAccess = Public`
`Matlab documentation of property attributes.`
Default: struct.empty("")

Definition at line 402 of file amimodel.m.

7.4.4.36 o2flag = 0

Note

This property has non-standard access specifiers: `SetAccess = Private, GetAccess = Public`
`Matlab documentation of property attributes.`
Default: 0

Definition at line 414 of file amimodel.m.

7.4.4.37 compver = 9

Note

This property has non-standard access specifiers: `SetAccess = Private, GetAccess = Public`
`Matlab documentation of property attributes.`
Default: 9

Definition at line 431 of file amimodel.m.

7.4.4.38 z2event = double.empty("")

Default: double.empty("")

Definition at line 446 of file amimodel.m.

7.4.4.39 splineflag = false

Default: false

Definition at line 454 of file amimodel.m.

7.4.4.40 minflag = false

Default: false

Definition at line 462 of file amimodel.m.

7.4.4.41 maxflag = false

Default: false

Definition at line 470 of file amimodel.m.

7.4.4.42 nw = 0

Default: 0

Definition at line 478 of file amimodel.m.

7.4.4.43 ndwdx = 0

Default: 0

Definition at line 487 of file amimodel.m.

7.4.4.44 ndwdp = 0

Default: 0

Definition at line 495 of file amimodel.m.

7.5 amioption Class Reference

AMIOPTION provides an option container to pass simulation parameters to the simulation routine.

Inheritance diagram for amioption:

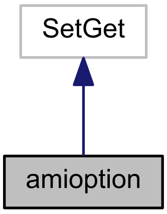

Collaboration diagram for amioption:

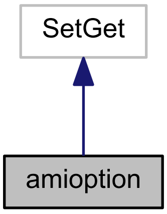

Public Member Functions

- amioption (matlabtypesubstitute varargin)

    amioptions Construct a new amioptions object

Public Attributes

- matlabtypesubstitute atol = 1e-16

    absolute integration tolerace
- matlabtypesubstitute rtol = 1e-8

    relative integration tolerace
- matlabtypesubstitute maxsteps = 1e4

    maximum number of integration steps
- matlabtypesubstitute sens_ind = double.empty("")

    index of parameters for which the sensitivities are computed
- matlabtypesubstitute qpositivex = double.empty("")

    index of states for which positivity should be enforced (currently this has no effect)
- matlabtypesubstitute tstart = 0

    starting time of the simulation
- matlabtypesubstitute lmm = 2

linear multistep method.

- matlabtypesubstitute iter = 2

  iteration method for linear multistep.

- matlabtypesubstitute linsol = 9

  linear solver

- matlabtypesubstitute stldet = true

  stability detection flag

- matlabtypesubstitute interpType = 1

  interpolation type

- matlabtypesubstitute lmmB = 2

  linear multistep method (backwards)

- matlabtypesubstitute iterB = 2

  iteration method for linear multistep (backwards).

- matlabtypesubstitute ism = 1

  forward sensitivity mode

- matlabtypesubstitute sensi_meth = 1

  sensitivity method

- matlabtypesubstitute sensi = 0

  sensitivity order

- matlabtypesubstitute nmaxevent = 10

  number of reported events

- matlabtypesubstitute ordering = 1

  reordering of states

- matlabtypesubstitute ss = 0

  steady state sensitivity flag

- matlabtypesubstitute sx0 = double.empty("")

  custom initial sensitivity

- matlabtypesubstitute z2event = double.empty("")

  mapping of event ouputs to events

- matlabtypesubstitute id = double.empty("")

  flag for DAE variables

7.5.1 Detailed Description

Definition at line 17 of file amioption.m.

7.5.2 Constructor & Destructor Documentation

7.5.2.1 amioption (matlabtypesubstitute *varargin*)

OPTS = amioption() creates a set of options with each option set to its default value.

OPTS = amioption(PARAM, VAL, ...) creates a set of options with the named parameters altered with the specified values.

OPTS = amioption(OLDOPTS, PARAM, VAL, ...) creates a copy of OLDOPTS with the named parameters altered with the specified value

Note to see the parameters, check the documentation page for amioptions

Definition at line 217 of file amioption.m.

7.5.3 Member Data Documentation

7.5.3.1 atol = 1e-16

Default: 1e-16

Definition at line 28 of file amioption.m.

7.5.3.2 rtol = 1e-8

Default: 1e-8

Definition at line 36 of file amioption.m.

7.5.3.3 maxsteps = 1e4

Default: 1e4

Definition at line 44 of file amioption.m.

7.5.3.4 sens_ind = double.empty("")

Default: double.empty("")

Definition at line 52 of file amioption.m.

7.5.3.5 qpositivex = double.empty("")

Default: double.empty("")

Definition at line 60 of file amioption.m.

7.5.3.6 tstart = 0

Default: 0

Definition at line 69 of file amioption.m.

7.5.3.7 lmm = 2

Default: 2

Definition at line 77 of file amioption.m.

7.5.3.8 iter = 2

Default: 2

Definition at line 85 of file amioption.m.

7.5.3.9 linsol = 9

Default: 9

Definition at line 93 of file amioption.m.

7.5.3.10 stldet = true

Default: true

Definition at line 101 of file amioption.m.

7.5.3.11 interpType = 1

Default: 1

Definition at line 109 of file amioption.m.

7.5.3.12 lmmB = 2

Default: 2

Definition at line 117 of file amioption.m.

7.5.3.13 iterB = 2

Default: 2

Definition at line 125 of file amioption.m.

7.5.3.14 ism = 1

Default: 1

Definition at line 133 of file amioption.m.

7.5.3.15 sensi_meth = 1

Default: 1

Note

This property has custom functionality when its value is changed.

Definition at line 141 of file amioption.m.

7.5.3.16 sensi = 0

Default: 0

Note

This property has custom functionality when its value is changed.

Definition at line 149 of file amioption.m.

7.5.3.17 nmaxevent = 10

Default: 10

Definition at line 157 of file amioption.m.

7.5.3.18 ordering = 1

Default: 1

Definition at line 165 of file amioption.m.

7.5.3.19 ss = 0

Default: 0

Definition at line 173 of file amioption.m.

7.5.3.20 sx0 = double.empty("")

Default: double.empty("")

Definition at line 181 of file amioption.m.

7.5.3.21 z2event = double.empty("")

Note

This property has the MATLAB attribute `Hidden` set to true.
`Matlab documentation of property attributes.`
Default: double.empty("")

Definition at line 192 of file amioption.m.

7.5.3.22 id = double.empty("")

Note

This property has the MATLAB attribute `Hidden` set to true.
`Matlab documentation of property attributes.`
Default: double.empty("")

Definition at line 203 of file amioption.m.

7.6 ExpData Struct Reference

struct that carries all information about experimental data

```
#include <edata.h>
```

Public Attributes

- double * am_my
- double * am_ysigma
- double * am_mz
- double * am_zsigma

7.6.1 Detailed Description

Definition at line 18 of file edata.h.

7.6.2 Member Data Documentation

7.6.2.1 double* am_my

observed data

Definition at line 20 of file edata.h.

7.6.2.2 double* am_ysigma

standard deviation of observed data

Definition at line 22 of file edata.h.

7.6.2.3 double* am_mz

observed events

Definition at line 25 of file edata.h.

7.6.2.4 double* am_zsigma

standard deviation of observed events

Definition at line 27 of file edata.h.

7.7 modelTest Class Reference

MODELTEST Summary of this class goes here Detailed explanation goes here.

7.7.1 Detailed Description

Definition at line 17 of file modelTest.m.

7.8 optsym Class Reference

OPTSYM is an auxiliary class to gain access to the private symbolic property `s` which is necessary to be able to call symobj::optimize on it.

Inheritance diagram for optsym:

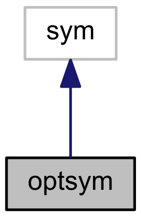

Collaboration diagram for optsym:

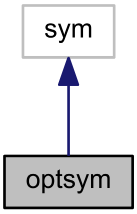

Public Member Functions

- optsym (matlabtypesubstitute symbol)

  optsym converts the symbolic object into a optsym object
- mlhsInnerSubst< matlabtypesubstitute > getoptimized ()

  optsym calls symobj::optimize on the optsym object

7.8.1 Detailed Description

Definition at line 17 of file optsym.m.

7.8.2 Member Function Documentation

7.8.2.1 mlhsInnerSubst< matlabtypesubstitute > getoptimized ( )

Return values

optimized	symbolic object

Definition at line 46 of file optsym.m.

7.9 ReturnData Struct Reference

struct that stores all data which is later returned by the mex function

```
#include <rdata.h>
```

Public Attributes

- double * am_tsdata
- double * am_xdotdata
- double * am_dxdotdpdata
- double * am_dydxdata
- double * am_dydpdata
- double * am_Jdata
- double * am_zdata
- double * am_sigmazdata

- double * am_szdata
- double * am_ssigmazdata
- double * am_xdata
- double * am_sxdata
- double * am_ydata
- double * am_sigmaydata
- double * am_sydata
- double * am_ssigmaydata
- double * am_numstepsdata
- double * am_numstepsSdata
- double * am_numrhsevalsdata
- double * am_numrhsevalsSdata
- double * am_orderdata
- double * am_llhdata
- double * am_chi2data
- double * am_sllhdata
- double * am_s2llhdata

7.9.1 Detailed Description

Definition at line 42 of file rdata.h.

7.9.2 Member Data Documentation

7.9.2.1 double* am_tsdata

timepoints

Definition at line 45 of file rdata.h.

7.9.2.2 double* am_xdotdata

time derivative

Definition at line 47 of file rdata.h.

7.9.2.3 double* am_dxdotdpdata

parameter derivative of time derivative

Definition at line 49 of file rdata.h.

7.9.2.4 double* am_dydxdata

state derivative of observables

Definition at line 51 of file rdata.h.

7.9.2.5 double* am_dydpdata

parameter derivative of observables

Definition at line 53 of file rdata.h.

7.9.2.6 double* am_Jdata

Jacobian of differential equation right hand side

Definition at line 55 of file rdata.h.

7.9.2.7 double∗ am_zdata

event output

Definition at line 57 of file rdata.h.

7.9.2.8 double∗ am_sigmazdata

event output sigma standard deviation

Definition at line 59 of file rdata.h.

7.9.2.9 double∗ am_szdata

parameter derivative of event output

Definition at line 61 of file rdata.h.

7.9.2.10 double∗ am_ssigmazdata

parameter derivative of event output standard deviation

Definition at line 63 of file rdata.h.

7.9.2.11 double∗ am_xdata

state

Definition at line 65 of file rdata.h.

7.9.2.12 double∗ am_sxdata

parameter derivative of state

Definition at line 67 of file rdata.h.

7.9.2.13 double∗ am_ydata

observable

Definition at line 69 of file rdata.h.

7.9.2.14 double∗ am_sigmaydata

observable standard deviation

Definition at line 71 of file rdata.h.

7.9.2.15 double∗ am_sydata

parameter derivative of observable

Definition at line 73 of file rdata.h.

7.9.2.16 double∗ am_ssigmaydata

parameter derivative of observable standard deviation

Definition at line 75 of file rdata.h.

7.9.2.17 double∗ am_numstepsdata

number of integration steps forward problem

Definition at line 78 of file rdata.h.

7.9.2.18 double* am_numstepsSdata

number of integration steps backward problem

Definition at line 80 of file rdata.h.

7.9.2.19 double* am_numrhsevalsdata

number of right hand side evaluations forward problem

Definition at line 82 of file rdata.h.

7.9.2.20 double* am_numrhsevalsSdata

number of right hand side evaluations backwad problem

Definition at line 84 of file rdata.h.

7.9.2.21 double* am_orderdata

employed order forward problem

Definition at line 86 of file rdata.h.

7.9.2.22 double* am_llhdata

likelihood value

Definition at line 89 of file rdata.h.

7.9.2.23 double* am_chi2data

chi2 value

Definition at line 91 of file rdata.h.

7.9.2.24 double* am_sllhdata

parameter derivative of likelihood

Definition at line 93 of file rdata.h.

7.9.2.25 double* am_s2llhdata

second order parameter derivative of likelihood

Definition at line 95 of file rdata.h.

7.10 SBMLode Class Reference

SBMLMODEL provides an intermediate container between the SBML definition and an amimodel object.

Inheritance diagram for SBMLode:

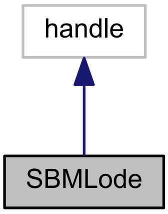

Collaboration diagram for SBMLode:

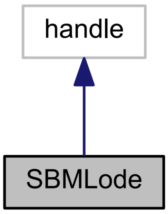

Public Member Functions

- SBMLode (matlabtypesubstitute filename)

    SBMLode extracts information from an SBML definition and stores it in a symbolic format.
- noret::substitute writeAMICI (matlabtypesubstitute filename, matlabtypesubstitute this, matlabtypesubstitute modelname)

    writeAMICI writes the symbolic information from an SBMLode object into an AMICI model definition file

Public Attributes

- matlabtypesubstitute state = sym.empty("")

    states
- matlabtypesubstitute observable = sym.empty("")

    observables
- matlabtypesubstitute observable_name = sym.empty("")

    names of observables
- matlabtypesubstitute param = sym.empty("")

    parameter names
- matlabtypesubstitute parameter = sym.empty("")

    parameter expressions

- matlabtypesubstitute constant = sym.empty("")
  constants
- matlabtypesubstitute reaction = sym.empty("")
  reactions
- matlabtypesubstitute compartment = sym.empty("")
  compartments
- matlabtypesubstitute volume = sym.empty("")
  compartment volumes
- matlabtypesubstitute initState = sym.empty("")
  initial condition of states
- matlabtypesubstitute condition = sym.empty("")
  condition
- matlabtypesubstitute flux = sym.empty("")
  reaction fluxes
- matlabtypesubstitute stochiometry = sym.empty("")
  reaction stochiometry
- matlabtypesubstitute xdot = sym.empty("")
  right hand side of reconstructed differential equation
- matlabtypesubstitute trigger = sym.empty("")
  event triggers
- matlabtypesubstitute bolus = sym.empty("")
  event boli
- matlabtypesubstitute funmath = cell.empty("")
  mathematical experessions for function
- matlabtypesubstitute **funarg** = cell.empty("")
- matlabtypesubstitute time_symbol = char.empty("")
  symbol of time
- matlabtypesubstitute pnom = double.empty("")
  nominal parameters
- matlabtypesubstitute knom = double.empty("")
  nominal conditions

7.10.1 Detailed Description

Definition at line 17 of file SBMLode.m.

7.10.2 Constructor & Destructor Documentation

7.10.2.1 SBMLode (matlabtypesubstitute *filename*)

Parameters

filename	target name of the model (excluding the suffix .xml/.sbml)

Definition at line 193 of file SBMLode.m.

7.10.3 Member Function Documentation

7.10.3.1 noret::substitute writeAMICI (matlabtypesubstitute *filename,* matlabtypesubstitute *this,* matlabtypesubstitute *modelname*)

Parameters

modelname	target name of the model (_syms.m will be appended to the name)

Definition at line 18 of file writeAMICI.m.

7.10.4 Member Data Documentation

7.10.4.1 state = sym.empty("")

Default: sym.empty("")

Definition at line 28 of file SBMLode.m.

7.10.4.2 observable = sym.empty("")

Default: sym.empty("")

Definition at line 36 of file SBMLode.m.

7.10.4.3 observable_name = sym.empty("")

Default: sym.empty("")

Definition at line 44 of file SBMLode.m.

7.10.4.4 param = sym.empty("")

Default: sym.empty("")

Definition at line 52 of file SBMLode.m.

7.10.4.5 parameter = sym.empty("")

Default: sym.empty("")

Definition at line 60 of file SBMLode.m.

7.10.4.6 constant = sym.empty("")

Default: sym.empty("")

Definition at line 68 of file SBMLode.m.

7.10.4.7 reaction = sym.empty("")

Default: sym.empty("")

Definition at line 76 of file SBMLode.m.

7.10.4.8 compartment = sym.empty("")

Default: sym.empty("")

Definition at line 84 of file SBMLode.m.

7.10.4.9 volume = sym.empty("")

Default: sym.empty("")

Definition at line 92 of file SBMLode.m.

7.10.4.10 initState = sym.empty("")

Default: sym.empty("")

Definition at line 100 of file SBMLode.m.

7.10.4.11 condition = sym.empty("")

Default: sym.empty("")

Definition at line 108 of file SBMLode.m.

7.10.4.12 flux = sym.empty("")

Default: sym.empty("")

Definition at line 116 of file SBMLode.m.

7.10.4.13 stochiometry = sym.empty("")

Default: sym.empty("")

Definition at line 124 of file SBMLode.m.

7.10.4.14 xdot = sym.empty("")

Default: sym.empty("")

Definition at line 132 of file SBMLode.m.

7.10.4.15 trigger = sym.empty("")

Default: sym.empty("")

Definition at line 140 of file SBMLode.m.

7.10.4.16 bolus = sym.empty("")

Default: sym.empty("")

Definition at line 148 of file SBMLode.m.

7.10.4.17 funmath = cell.empty("")

Default: cell.empty("")

Definition at line 156 of file SBMLode.m.

7.10.4.18 time_symbol = char.empty("")

Default: char.empty("")

Definition at line 166 of file SBMLode.m.

7.10.4.19 pnom = double.empty("")

Default: double.empty("")

Definition at line 174 of file SBMLode.m.

7.10.4.20 knom = double.empty("")

Default: double.empty("")

Definition at line 182 of file SBMLode.m.

7.11 TempData Struct Reference

struct that provides temporary storage for different variables

```
#include <tdata.h>
```

Public Attributes

- realtype am_t
- N_Vector am_x
- N_Vector am_x_old
- N_Vector * am_x_disc
- N_Vector * am_xdot_disc
- N_Vector * am_xdot_old_disc
- N_Vector am_dx
- N_Vector am_dx_old
- N_Vector am_xdot
- N_Vector am_xdot_old
- N_Vector am_xB
- N_Vector am_xB_old
- N_Vector am_dxB
- N_Vector am_xQB
- N_Vector am_xQB_old
- N_Vector * am_sx
- N_Vector * am_sdx
- N_Vector am_id
- DlsMat am_Jtmp
- realtype * am_llhS0
- realtype am_g
- realtype * am_dgdp
- realtype * am_dgdx
- realtype am_r
- realtype * am_drdp
- realtype * am_drdx
- realtype am_rval
- realtype * am_drvaldp

- realtype * am_drvaldx
- realtype * am_dzdx
- realtype * am_dzdp
- realtype * am_dydp
- realtype * am_dydx
- realtype * am_yS0
- realtype * am_sigma_y
- realtype * am_dsigma_ydp
- realtype * am_sigma_z
- realtype * am_dsigma_zdp
- realtype * am_x_tmp
- realtype * am_sx_tmp
- realtype * am_dx_tmp
- realtype * am_sdx_tmp
- realtype * am_xdot_tmp
- realtype * am_xB_tmp
- realtype * am_xQB_tmp
- realtype * am_dxB_tmp
- realtype * am_id_tmp
- int * am_rootsfound
- int * am_rootidx
- int * am_nroots
- double * am_rootvals
- realtype * am_deltax
- realtype * am_deltasx
- realtype * am_deltaxB
- realtype * am_deltaqB
- int am_which
- realtype * am_discs
- realtype * am_irdiscs

7.11.1 Detailed Description

Definition at line 78 of file tdata.h.

7.11.2 Member Data Documentation

7.11.2.1 realtype am_t

current time

Definition at line 80 of file tdata.h.

7.11.2.2 N_Vector am_x

state vector

Definition at line 84 of file tdata.h.

7.11.2.3 N_Vector am_x_old

old state vector

Definition at line 86 of file tdata.h.

7.11.2.4 N_Vector* am_x_disc

array of state vectors at discontinuities

Definition at line 88 of file tdata.h.

7.11.2.5 N_Vector* am_xdot_disc

array of differential state vectors at discontinuities

Definition at line 90 of file tdata.h.

7.11.2.6 N_Vector* am_xdot_old_disc

array of old differential state vectors at discontinuities

Definition at line 92 of file tdata.h.

7.11.2.7 N_Vector am_dx

differential state vector

Definition at line 94 of file tdata.h.

7.11.2.8 N_Vector am_dx_old

old differential state vector

Definition at line 96 of file tdata.h.

7.11.2.9 N_Vector am_xdot

time derivative state vector

Definition at line 98 of file tdata.h.

7.11.2.10 N_Vector am_xdot_old

old time derivative state vector

Definition at line 100 of file tdata.h.

7.11.2.11 N_Vector am_xB

adjoint state vector

Definition at line 102 of file tdata.h.

7.11.2.12 N_Vector am_xB_old

old adjoint state vector

Definition at line 104 of file tdata.h.

7.11.2.13 N_Vector am_dxB

differential adjoint state vector

Definition at line 106 of file tdata.h.

7.11.2.14 N_Vector am_xQB

quadrature state vector

Definition at line 108 of file tdata.h.

7.11.2.15 N_Vector am_xQB_old

old quadrature state vector

Definition at line 110 of file tdata.h.

7.11.2.16 N_Vector* am_sx

sensitivity state vector array

Definition at line 112 of file tdata.h.

7.11.2.17 N_Vector* am_sdx

differential sensitivity state vector array

Definition at line 114 of file tdata.h.

7.11.2.18 N_Vector am_id

index indicating DAE equations vector

Definition at line 116 of file tdata.h.

7.11.2.19 DlsMat am_Jtmp

Jacobian

Definition at line 118 of file tdata.h.

7.11.2.20 realtype* am_llhS0

parameter derivative of likelihood array

Definition at line 121 of file tdata.h.

7.11.2.21 realtype am_g

data likelihood

Definition at line 123 of file tdata.h.

7.11.2.22 realtype* am_dgdp

parameter derivative of data likelihood

Definition at line 125 of file tdata.h.

7.11.2.23 realtype* am_dgdx

state derivative of data likelihood

Definition at line 127 of file tdata.h.

7.11.2.24 realtype am_r

event likelihood

Definition at line 129 of file tdata.h.

7.11.2.25 realtype* am_drdp

parameter derivative of event likelihood

Definition at line 131 of file tdata.h.

7.11.2.26 realtype* am_drdx

state derivative of event likelihood

Definition at line 133 of file tdata.h.

7.11.2.27 realtype am_rval

root function likelihood

Definition at line 135 of file tdata.h.

7.11.2.28 realtype* am_drvaldp

parameter derivative of root function likelihood

Definition at line 137 of file tdata.h.

7.11.2.29 realtype* am_drvaldx

state derivative of root function likelihood

Definition at line 139 of file tdata.h.

7.11.2.30 realtype* am_dzdx

state derivative of event

Definition at line 141 of file tdata.h.

7.11.2.31 realtype* am_dzdp

parameter derivative of event

Definition at line 143 of file tdata.h.

7.11.2.32 realtype* am_dydp

parameter derivative of observable

Definition at line 145 of file tdata.h.

7.11.2.33 realtype* am_dydx

state derivative of observable

Definition at line 147 of file tdata.h.

7.11.2.34 realtype* am_yS0

initial sensitivity of observable

Definition at line 149 of file tdata.h.

7.11.2.35 realtype* am_sigma_y

data standard deviation

Definition at line 151 of file tdata.h.

7.11.2.36 realtype* am_dsigma_ydp

parameter derivative of data standard deviation

Definition at line 153 of file tdata.h.

7.11.2.37 realtype* am_sigma_z

event standard deviation

Definition at line 155 of file tdata.h.

7.11.2.38 realtype* am_dsigma_zdp

parameter derivative of event standard deviation

Definition at line 157 of file tdata.h.

7.11.2.39 realtype* am_x_tmp

state array

Definition at line 160 of file tdata.h.

7.11.2.40 realtype* am_sx_tmp

sensitivity state array

Definition at line 162 of file tdata.h.

7.11.2.41 realtype* am_dx_tmp

differential state array

Definition at line 164 of file tdata.h.

7.11.2.42 realtype* am_sdx_tmp

differential sensitivity state array

Definition at line 166 of file tdata.h.

7.11.2.43 realtype* am_xdot_tmp

time derivative state array

Definition at line 168 of file tdata.h.

7.11.2.44 realtype* am_xB_tmp

differential adjoint state array

Definition at line 170 of file tdata.h.

7.11.2.45 realtype* am_xQB_tmp

quadrature state array

Definition at line 172 of file tdata.h.

7.11.2.46 realtype* am_dxB_tmp

differential adjoint state array

Definition at line 174 of file tdata.h.

7.11.2.47 realtype* am_id_tmp

index indicating DAE equations array

Definition at line 176 of file tdata.h.

7.11.2.48 int* am_rootsfound

array of flags indicating which root has beend found

array of length nr with the indices of the user functions gi found to have a root. For i = 0, . . . ,nr?1, rootsfound[i]?= 0 if gi has a root, and = 0 if not.

Definition at line 183 of file tdata.h.

7.11.2.49 int* am_rootidx

array of index which root has been found

Definition at line 185 of file tdata.h.

7.11.2.50 int* am_nroots

array of number of found roots for a certain event type

Definition at line 187 of file tdata.h.

7.11.2.51 double* am_rootvals

array of values of the root function

Definition at line 189 of file tdata.h.

7.11.2.52 realtype* am_deltax

change in x

Definition at line 193 of file tdata.h.

7.11.2.53 realtype* am_deltasx

change in sx

Definition at line 195 of file tdata.h.

7.11.2.54 realtype* am_deltaxB

change in xB

Definition at line 197 of file tdata.h.

7.11.2.55 realtype* am_deltaqB

change in qB

Definition at line 199 of file tdata.h.

7.11.2.56 int am_which

integer for indexing of backwards problems

Definition at line 203 of file tdata.h.

7.11.2.57 realtype* am_discs

array containing the time-points of discontinuities

Definition at line 206 of file tdata.h.

7.11.2.58 realtype* am_irdiscs

array containing the index of discontinuities

Definition at line 208 of file tdata.h.

7.12 UserData Struct Reference

struct that stores all user provided data

```
#include <udata.h>
```

Public Attributes

- double ∗ am_qpositivex
- int ∗ am_plist
- int am_np
- int am_ny
- int am_nytrue
- int am_nx
- int am_nz
- int am_nztrue
- int am_ne
- int am_nt
- int am_nw
- int am_ndwdx
- int am_ndwdp
- int am_nnz
- int am_nmaxevent
- double ∗ am_p
- double ∗ am_k
- double am_tstart
- double ∗ am_ts
- double ∗ am_pbar
- double ∗ am_xbar
- double ∗ am_idlist
- int am_sensi
- double am_atol
- double am_rtol
- int am_maxsteps
- int am_ism
- int am_sensi_meth
- int am_linsol
- int am_interpType
- int am_lmm
- int am_iter
- booleantype am_stldet
- int am_ubw
- int am_lbw
- booleantype am_bsx0
- double ∗ am_sx0data
- int am_event_model
- int am_ordering
- double ∗ am_z2event
- double ∗ am_h
- SlsMat am_J
- realtype ∗ am_dxdotdp
- realtype ∗ am_w

- realtype ∗ am_dwdx
- realtype ∗ am_dwdp
- realtype ∗ am_M
- realtype ∗ am_dfdx
- booleantype am_nan_dxdotdp
- booleantype am_nan_J
- booleantype am_nan_JSparse
- booleantype am_nan_xdot
- booleantype am_nan_xBdot
- booleantype am_nan_qBdot

7.12.1 Detailed Description

Definition at line 77 of file udata.h.

7.12.2 Member Data Documentation

7.12.2.1 double∗ am_qpositivex

positivity flag

Definition at line 79 of file udata.h.

7.12.2.2 int∗ am_plist

parameter reordering

Definition at line 82 of file udata.h.

7.12.2.3 int am_np

number of parameters

Definition at line 84 of file udata.h.

7.12.2.4 int am_ny

number of observables

Definition at line 86 of file udata.h.

7.12.2.5 int am_nytrue

number of observables in the unaugmented system

Definition at line 88 of file udata.h.

7.12.2.6 int am_nx

number of states

Definition at line 90 of file udata.h.

7.12.2.7 int am_nz

number of event outputs

Definition at line 92 of file udata.h.

7.12.2.8 int am_nztrue

number of event outputs in the unaugmented system

Definition at line 94 of file udata.h.

7.12.2.9 int am_ne

number of events

Definition at line 96 of file udata.h.

7.12.2.10 int am_nt

number of timepoints

Definition at line 98 of file udata.h.

7.12.2.11 int am_nw

number of common expressions

Definition at line 100 of file udata.h.

7.12.2.12 int am_ndwdx

number of derivatives of common expressions wrt x

Definition at line 102 of file udata.h.

7.12.2.13 int am_ndwdp

number of derivatives of common expressions wrt p

Definition at line 104 of file udata.h.

7.12.2.14 int am_nnz

number of nonzero entries in jacobian

Definition at line 106 of file udata.h.

7.12.2.15 int am_nmaxevent

maximal number of events to track

Definition at line 108 of file udata.h.

7.12.2.16 double* am_p

parameter array

Definition at line 111 of file udata.h.

7.12.2.17 double* am_k

constants array

Definition at line 113 of file udata.h.

7.12.2.18 double am_tstart

starting time

Definition at line 116 of file udata.h.

7.12.2.19 double* am_ts

timepoints

Definition at line 118 of file udata.h.

7.12.2.20 double* am_pbar

scaling of parameters

Definition at line 121 of file udata.h.

7.12.2.21 double* am_xbar

scaling of states

Definition at line 123 of file udata.h.

7.12.2.22 double* am_idlist

flag array for DAE equations

Definition at line 126 of file udata.h.

7.12.2.23 int am_sensi

flag indicating whether sensitivities are supposed to be computed

Definition at line 129 of file udata.h.

7.12.2.24 double am_atol

absolute tolerances for integration

Definition at line 131 of file udata.h.

7.12.2.25 double am_rtol

relative tolerances for integration

Definition at line 133 of file udata.h.

7.12.2.26 int am_maxsteps

maximum number of allowed integration steps

Definition at line 135 of file udata.h.

7.12.2.27 int am_ism

internal sensitivity method

a flag used to select the sensitivity solution method. Its value can be CV SIMULTANEOUS or CV STAGGERED. Only applies for Forward Sensitivities.

Definition at line 141 of file udata.h.

7.12.2.28 int am_sensi_meth

method for sensitivity computation

CW_FSA for forward sensitivity analysis, CW_ASA for adjoint sensitivity analysis

Definition at line 147 of file udata.h.

7.12.2.29 int am_linsol

linear solver specification

Definition at line 149 of file udata.h.

7.12.2.30 int am_interpType

interpolation type

specifies the interpolation type for the forward problem solution which is then used for the backwards problem. can be either CV_POLYNOMIAL or CV_HERMITE

Definition at line 154 of file udata.h.

7.12.2.31 int am_lmm

linear multistep method

specifies the linear multistep method and may be one of two possible values: CV ADAMS or CV BDF.

Definition at line 160 of file udata.h.

7.12.2.32 int am_iter

nonlinear solver

specifies the type of nonlinear solver iteration and may be either CV NEWTON or CV FUNCTIONAL.

Definition at line 166 of file udata.h.

7.12.2.33 booleantype am_stldet

flag controlling stability limit detection

Definition at line 169 of file udata.h.

7.12.2.34 int am_ubw

upper bandwith of the jacobian

Definition at line 172 of file udata.h.

7.12.2.35 int am_lbw

lower bandwith of the jacobian

Definition at line 174 of file udata.h.

7.12.2.36 booleantype am_bsx0

flag for sensitivity initialisation

flag which determines whether analytic sensitivities initialisation or provided initialisation should be used

Definition at line 180 of file udata.h.

7.12.2.37 double* am_sx0data

sensitivity initialisation

Definition at line 183 of file udata.h.

7.12.2.38 int am_event_model

error model for events

Definition at line 186 of file udata.h.

7.12.2.39 int am_ordering

state ordering

Definition at line 189 of file udata.h.

7.12.2.40 double∗ am_z2event

index indicating to which event an event output belongs

Definition at line 192 of file udata.h.

7.12.2.41 double∗ am_h

flag indicating whether a certain heaviside function should be active or not

Definition at line 195 of file udata.h.

7.12.2.42 SlsMat am_J

tempory storage of Jacobian data across functions

Definition at line 198 of file udata.h.

7.12.2.43 realtype∗ am_dxdotdp

tempory storage of dxdotdp data across functions

Definition at line 200 of file udata.h.

7.12.2.44 realtype∗ am_w

tempory storage of w data across functions

Definition at line 202 of file udata.h.

7.12.2.45 realtype∗ am_dwdx

tempory storage of dwdx data across functions

Definition at line 204 of file udata.h.

7.12.2.46 realtype∗ am_dwdp

tempory storage of dwdp data across functions

Definition at line 206 of file udata.h.

7.12.2.47 realtype∗ am_M

tempory storage of M data across functions

Definition at line 208 of file udata.h.

7.12.2.48 realtype∗ am_dfdx

tempory storage of dfdx data across functions

Definition at line 210 of file udata.h.

7.12.2.49 booleantype am_nan_dxdotdp

flag indicating whether a NaN in dxdotdp has been reported

Definition at line 213 of file udata.h.

7.12.2.50 booleantype am_nan_J

flag indicating whether a NaN in J has been reported

Definition at line 215 of file udata.h.

7.12.2.51 booleantype am_nan_JSparse

flag indicating whether a NaN in JSparse has been reported

Definition at line 217 of file udata.h.

7.12.2.52 booleantype am_nan_xdot

flag indicating whether a NaN in xdot has been reported

Definition at line 219 of file udata.h.

7.12.2.53 booleantype am_nan_xBdot

flag indicating whether a NaN in xBdot has been reported

Definition at line 221 of file udata.h.

7.12.2.54 booleantype am_nan_qBdot

flag indicating whether a NaN in qBdot has been reported

Definition at line 223 of file udata.h.

8 File Documentation

8.1 amiwrap.c File Reference

core routines for mex interface

```
#include <stdio.h>
#include <stdlib.h>
#include <string.h>
#include <math.h>
#include <mex.h>
#include "wrapfunctions.h"
#include <include/amici.h>
```

Include dependency graph for amiwrap.c:

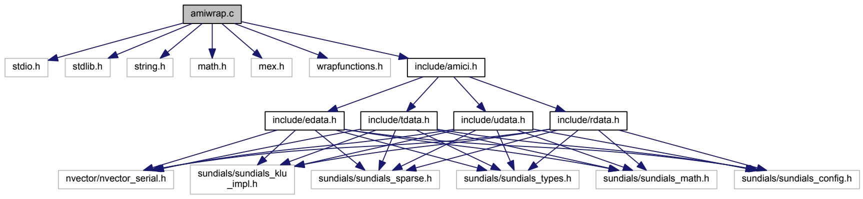

This graph shows which files directly or indirectly include this file:

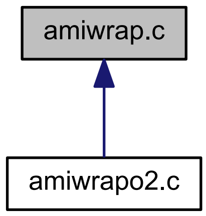

Macros

- #define **_USE_MATH_DEFINES** /∗ MS definition of PI and other constants ∗/
- #define **M_PI** 3.14159265358979323846

Functions

- void mexFunction (int nlhs, mxArray ∗plhs[ ], int nrhs, const mxArray ∗prhs[ ])

8.1.1 Detailed Description

This file defines the fuction mexFunction which is executed upon calling the mex file from matlab

8.1.2 Function Documentation

8.1.2.1 void mexFunction (int *nlhs*, mxArray ∗ *plhs[ ]*, int *nrhs*, const mxArray ∗ *prhs[ ]*)

mexFunction is the main function of the mex simulation file this function carries out all numerical integration and writes results into the sol struct.

Parameters

in	*nlhs*	number of output arguments of the matlab call<br>**Type**: int
out	*plhs*	pointer to the array of output arguments<br>**Type**: mxArray
in	*nrhs*	number of input arguments of the matlab call<br>**Type**: int
in	*prhs*	pointer to the array of input arguments<br>**Type**: mxArray

Returns

void

Definition at line 29 of file amiwrap.c.

Here is the call graph for this function:

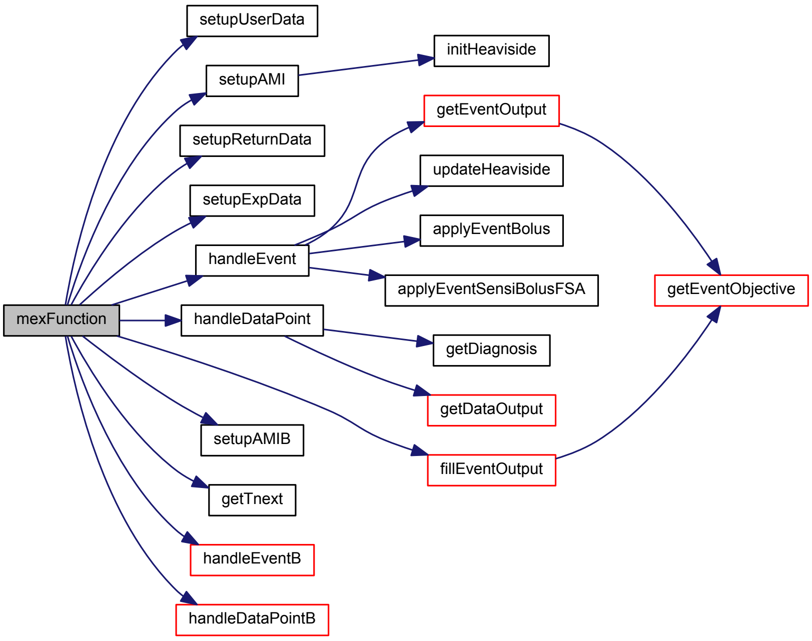

8.2 amiwrap.m File Reference

AMIWRAP generates c mex files for the simulation of systems of differential equations via CVODES and IDAS.

Functions

- noret::substitute amiwrap (matlabtypesubstitute varargin)

  AMIWRAP generates c mex files for the simulation of systems of differential equations via CVODES and IDAS.

8.2.1 Function Documentation

8.2.1.1 noret::substitute amiwrap (matlabtypesubstitute *varargin*)

Parameters

varargin	`1 amiwrap ( modelname, symfun, tdir, o2flag )` *Required Parameters for varargin:* • modelname specifies the name of the model which will be later used for the naming of the simualation file • symfun specifies a function which executes model defition see Model Definition for details • tdir target directory where the simulation file should be placed **Default:** $AMICIDIR/models/modelname • o2flag boolean whether second order sensitivities should be enabled **Default:** false

Return values

o2flag	void

Definition at line 17 of file amiwrap.m.

8.3 SBML2AMICI.m File Reference

SBML2AMICI generates AMICI model definition files from SBML.

Functions

- noret::substitute SBML2AMICI (matlabtypesubstitute filename, matlabtypesubstitute modelname)

  SBML2AMICI generates AMICI model definition files from SBML.

8.3.1 Function Documentation

8.3.1.1 noret::substitute SBML2AMICI (matlabtypesubstitute *filename,* matlabtypesubstitute *modelname*)

Parameters

filename	name of the SBML file (withouth extension)
modelname	name of the model, this will define the name of the output file

Return values

modelname	void

Definition at line 17 of file SBML2AMICI.m.

8.4 src/amici.c File Reference

core routines for integration

```
#include <stdio.h>
#include <stdlib.h>
#include <string.h>
#include <math.h>
#include <mex.h>
#include "wrapfunctions.h"
#include <include/amici.h>
```

Include dependency graph for amici.c:

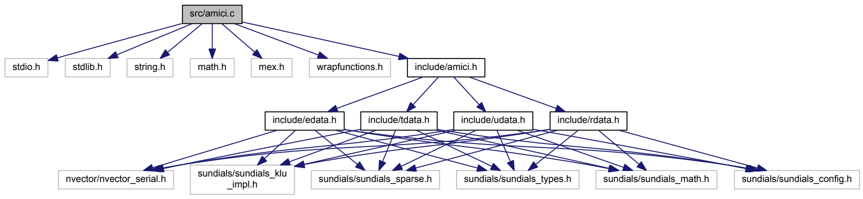

Macros

- #define **_USE_MATH_DEFINES** /∗ MS definition of PI and other constants ∗/
- #define **M_PI** 3.14159265358979323846
- #define initField2(FIELD, D1, D2)
- #define initField3(FIELD, D1, D2, D3)
- #define readOptionScalar(OPTION, TYPE)
- #define readOptionData(OPTION)
- #define AMI_SUCCESS 0

Functions

- UserData setupUserData (const mxArray ∗prhs[ ])
- ReturnData setupReturnData (mxArray ∗plhs[ ], void ∗user_data, double ∗pstatus)
- ExpData setupExpData (const mxArray ∗prhs[ ], void ∗user_data)
- void ∗ setupAMI (int ∗status, void ∗user_data, void ∗temp_data)
- void setupAMIB (int ∗status, void ∗ami_mem, void ∗user_data, void ∗temp_data)
- void getDataSensisFSA (int ∗status, int it, void ∗ami_mem, void ∗user_data, void ∗return_data, void ∗exp_data, void ∗temp_data)
- void getDataSensisASA (int ∗status, int it, void ∗ami_mem, void ∗user_data, void ∗return_data, void ∗exp_data, void ∗temp_data)
- void getDataOutput (int ∗status, int it, void ∗ami_mem, void ∗user_data, void ∗return_data, void ∗exp_data, void ∗temp_data)
- void getEventSensisFSA (int ∗status, int ie, void ∗ami_mem, void ∗user_data, void ∗return_data, void ∗temp_data)
- void getEventSensisFSA_tf (int ∗status, int ie, void ∗ami_mem, void ∗user_data, void ∗return_data, void ∗temp_data)
- void getEventSensisASA (int ∗status, int ie, void ∗ami_mem, void ∗user_data, void ∗return_data, void ∗exp_data, void ∗temp_data)
- void getEventSigma (int ∗status, int ie, int iz, void ∗ami_mem, void ∗user_data, void ∗return_data, void ∗exp_data, void ∗temp_data)
- void getEventObjective (int ∗status, int ie, void ∗ami_mem, void ∗user_data, void ∗return_data, void ∗exp_data, void ∗temp_data)
- void getEventOutput (int ∗status, realtype ∗tlastroot, void ∗ami_mem, void ∗user_data, void ∗return_data, void ∗exp_data, void ∗temp_data)
- void fillEventOutput (int ∗status, void ∗ami_mem, void ∗user_data, void ∗return_data, void ∗exp_data, void ∗temp_data)

- void handleDataPoint (int *status, int it, void *ami_mem, void *user_data, void *return_data, void *exp_data, void *temp_data)
- void handleDataPointB (int *status, int it, void *ami_mem, void *user_data, void *return_data, void *temp_data)
- void handleEvent (int *status, int iroot, realtype *tlastroot, void *ami_mem, void *user_data, void *return_data, void *exp_data, void *temp_data)
- void handleEventB (int *status, int iroot, void *ami_mem, void *user_data, void *temp_data)
- realtype getTnext (realtype *troot, int iroot, realtype *tdata, int it, void *user_data)
- void applyEventBolus (int *status, void *ami_mem, void *user_data, void *temp_data)
- void applyEventSensiBolusFSA (int *status, void *ami_mem, void *user_data, void *temp_data)
- void initHeaviside (int *status, void *user_data, void *temp_data)
- void updateHeaviside (int *status, void *user_data, void *temp_data)
- void updateHeavisideB (int *status, int iroot, void *user_data, void *temp_data)
- void getDiagnosis (int *status, int it, void *ami_mem, void *user_data, void *return_data)
- void getDiagnosisB (int *status, int it, void *ami_mem, void *user_data, void *return_data, void *temp_data)

8.4.1 Macro Definition Documentation

8.4.1.1 #define initField2(*FIELD, D1, D2*)

Value:

```
mxArray *mx ## FIELD; \
mx ## FIELD = mxCreateDoubleMatrix(D1,D2,mxREAL); \
FIELD ## data = mxGetPr(mx ## FIELD); \
mxSetField(mxsol,0,#FIELD,mx ## FIELD)
```

@ brief initialise matrix and attach to the field @ param FIELD name of the field to which the matrix will be attached @ param D1 number of rows in the matrix @ param D2 number of columns in the matrix

Definition at line 25 of file amici.c.

8.4.1.2 #define initField3(*FIELD, D1, D2, D3*)

Value:

```
mxArray *mx ## FIELD; \
const mwSize dims ## FIELD[]={D1,D2,D3}; \
mx ## FIELD = mxCreateNumericArray(3,dims ## FIELD,mxDOUBLE_CLASS,mxREAL); \
FIELD ## data = mxGetPr(mx ## FIELD); \
mxSetField(mxsol,0,#FIELD,mx ## FIELD)
```

@ brief initialise tensor and attach to the field @ param FIELD name of the field to which the tensor will be attached @ param D1 number of rows in the tensor @ param D2 number of columns in the tensor @ param D3 number of elements in the third dimension of the tensor

Definition at line 38 of file amici.c.

8.4.1.3 #define readOptionScalar(*OPTION, TYPE*)

Value:

```
if(mxGetProperty(prhs[3],0,#OPTION)){ \
    OPTION = (TYPE)mxGetScalar(mxGetProperty(prhs[3],0,#OPTION)); \
} else { \
    mexWarnMsgIdAndTxt("AMICI:mex:OPTION","Provided options are not of class amioption!"); \
    return(NULL); \
}
```

@ brief extract information from a property of a matlab class (scalar) @ param OPTION name of the property @ param TYPE class to which the information should be cast

Definition at line 50 of file amici.c.

8.4.1.4 #define readOptionData(*OPTION*)

Value:

```
if(mxGetProperty(prhs[3],0,#OPTION)){ \
    OPTION = mxGetData(mxGetProperty(prhs[3],0,#OPTION)); \
} else { \
    mexWarnMsgIdAndTxt("AMICI:mex:OPTION","Provided options are not of class amioption!"); \
    return(NULL); \
}
```

@ brief extract information from a property of a matlab class (matrix) @ param OPTION name of the property

Definition at line 62 of file amici.c.

8.4.1.5 #define AMI_SUCCESS 0

return value for successful execution

Definition at line 71 of file amici.c.

8.4.2 Function Documentation

8.4.2.1 UserData setupUserData (const mxArray * *prhs[ ]*)

setupUserData extracts information from the matlab call and returns the corresponding UserData struct

Parameters

in	*prhs*	pointer to the array of input arguments<br>**Type**: mxArray

Returns

udata: struct containing all provided user data
Type: UserData

Definition at line 73 of file amici.c.

Here is the caller graph for this function:

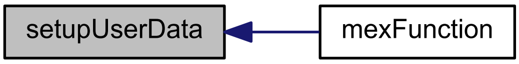

8.4.2.2 ReturnData setupReturnData (mxArray * *plhs[ ],* void * *user_data,* double * *pstatus*)

setupReturnData initialises the return data struct

Parameters

in	*plhs*	user input<br>**Type**: mxArray
in	*user_data*	pointer to the user data struct<br>**Type**: UserData
out	*pstatus*	pointer to the flag indicating the execution status<br>**Type**: double

Returns

rdata: return data struct
Type: ReturnData

user udata

Definition at line 217 of file amici.c.

Here is the caller graph for this function:

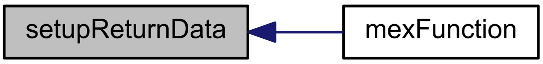

8.4.2.3 ExpData setupExpData (const mxArray ∗ *prhs[ ]*, void ∗ *user_data*)

setupExpData initialises the experimental data struct

Parameters

in	*prhs*	user input<br>**Type**: ∗mxArray
in	*user_data*	pointer to the user data struct<br>**Type**: UserData

Returns

edata: experimental data struct
Type: ExpData

user udata

Definition at line 315 of file amici.c.

Here is the caller graph for this function:

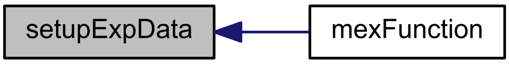

8.4.2.4 void* setupAMI (int * *status*, void * *user_data*, void * *temp_data*)

setupAMIs initialises the ami memory object

Parameters

out	*status*	flag indicating success of execution<br>**Type**: ∗int
in	*user_data*	pointer to the user data struct<br>**Type**: UserData
in	*temp_data*	pointer to the temporary data struct<br>**Type**: TempData

Returns

ami_mem pointer to the cvodes/idas memory block

Definition at line 422 of file amici.c.

Here is the call graph for this function:

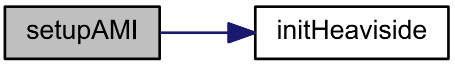

Here is the caller graph for this function:

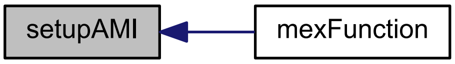

8.4.2.5 void setupAMIB (int ∗ *status*, void ∗ *ami_mem*, void ∗ *user_data*, void ∗ *temp_data*)

setupAMIB initialises the AMI memory object for the backwards problem

Parameters

out	*status*	flag indicating success of execution<br>**Type**: ∗int
in	*ami_mem*	pointer to the solver memory object of the forward problem
in	*user_data*	pointer to the user data struct<br>**Type**: UserData
in	*temp_data*	pointer to the temporary data struct<br>**Type**: TempData

Returns

ami_mem pointer to the cvodes/idas memory block for the backward problem

Definition at line 742 of file amici.c.

Here is the caller graph for this function:

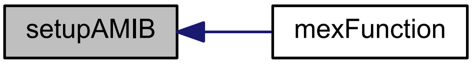

8.4.2.6 void getDataSensisFSA (int * *status,* int *it,* void * *ami_mem,* void * *user_data,* void * *return_data,* void * *exp_data,* void * *temp_data*)

getDataSensisFSA extracts data information for forward sensitivity analysis

Parameters

out	*status*	flag indicating success of execution<br>**Type**: *int
in	*it*	index of current timepoint<br>**Type**: int
in	*ami_mem*	pointer to the solver memory block<br>**Type**: *void
in	*user_data*	pointer to the user data struct<br>**Type**: UserData
out	*return_data*	pointer to the return data struct<br>**Type**: ReturnData
in	*exp_data*	pointer to the experimental data struct<br>**Type**: ExpData
out	*temp_data*	pointer to the temporary data struct<br>**Type**: TempData

Returns

void

Definition at line 937 of file amici.c.

Here is the caller graph for this function:

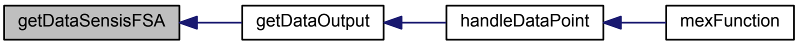

8.4.2.7 void getDataSensisASA (int * *status,* int *it,* void * *ami_mem,* void * *user_data,* void * *return_data,* void * *exp_data,* void * *temp_data*)

getDataSensisASA extracts data information for adjoint sensitivity analysis

Parameters

out	*status*	flag indicating success of execution<br>**Type**: ∗int
in	*it*	index of current timepoint<br>**Type**: int
in	*ami_mem*	pointer to the solver memory block<br>**Type**: ∗void
in	*user_data*	pointer to the user data struct<br>**Type**: UserData
out	*return_data*	pointer to the return data struct<br>**Type**: ReturnData
in	*exp_data*	pointer to the experimental data struct<br>**Type**: ExpData
out	*temp_data*	pointer to the temporary data struct<br>**Type**: TempData

Returns

void

Definition at line 999 of file amici.c.

Here is the caller graph for this function:

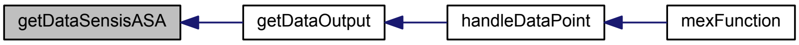

8.4.2.8 void getDataOutput (int ∗ *status*, int *it*, void ∗ *ami_mem*, void ∗ *user_data*, void ∗ *return_data*, void ∗ *exp_data*, void ∗ *temp_data*)

getDataOutput extracts output information for data-points

Parameters

out	*status*	flag indicating success of execution<br>**Type**: ∗int
in	*it*	index of current timepoint<br>**Type**: int
in	*ami_mem*	pointer to the solver memory block<br>**Type**: ∗void
in	*user_data*	pointer to the user data struct<br>**Type**: UserData
out	*return_data*	pointer to the return data struct<br>**Type**: ReturnData
in	*exp_data*	pointer to the experimental data struct<br>**Type**: ExpData
out	*temp_data*	pointer to the temporary data struct<br>**Type**: TempData

Returns

void

Definition at line 1050 of file amici.c.

Here is the call graph for this function:

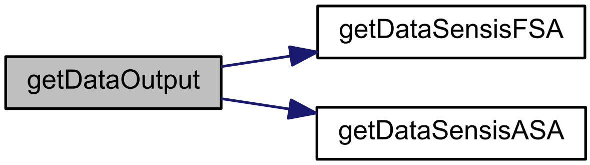

Here is the caller graph for this function:

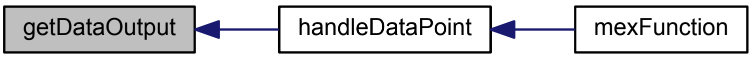

8.4.2.9 void getEventSensisFSA (int * *status*, int *ie*, void * *ami_mem*, void * *user_data*, void * *return_data*, void * *temp_data*)

getEventSensisFSA extracts event information for forward sensitivity analysis

Parameters

out	*status*	flag indicating success of execution<br>**Type**: int
in	*ie*	index of event type<br>**Type**: int
in	*ami_mem*	pointer to the solver memory block<br>**Type**: void
in	*user_data*	pointer to the user data struct<br>**Type**: UserData
out	*return_data*	pointer to the return data struct<br>**Type**: ReturnData
out	*temp_data*	pointer to the temporary data struct<br>**Type**: TempData

Returns

void

Definition at line 1105 of file amici.c.

Here is the caller graph for this function:

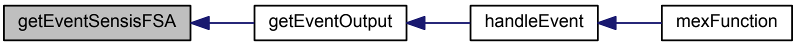

8.4.2.10 void getEventSensisFSA_tf (int * *status*, int *ie*, void * *ami_mem*, void * *user_data*, void * *return_data*, void * *temp_data*)

getEventSensisFSA_tf extracts event information for forward sensitivity analysis for events that happen at the end of the considered interval

Parameters

out	*status*	flag indicating success of execution<br>**Type**: int
in	*ie*	index of event type<br>**Type**: int
in	*ami_mem*	pointer to the solver memory block<br>**Type**: void
in	*user_data*	pointer to the user data struct<br>**Type**: UserData
out	*return_data*	pointer to the return data struct<br>**Type**: ReturnData
out	*temp_data*	pointer to the temporary data struct<br>**Type**: TempData

Returns

void

Definition at line 1135 of file amici.c.

Here is the caller graph for this function:

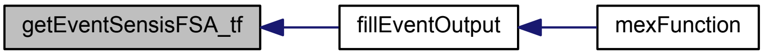

8.4.2.11 void getEventSensisASA (int * *status*, int *ie*, void * *ami_mem*, void * *user_data*, void * *return_data*, void * *exp_data*, void * *temp_data*)

getEventSensisASA extracts event information for adjoint sensitivity analysis

Parameters

out	*status*	flag indicating success of execution<br>**Type**: ∗int
in	*ie*	index of event type<br>**Type**: int
in	*ami_mem*	pointer to the solver memory block<br>**Type**: ∗void
in	*user_data*	pointer to the user data struct<br>**Type**: UserData
out	*return_data*	pointer to the return data struct<br>**Type**: ReturnData
in	*exp_data*	pointer to the experimental data struct<br>**Type**: ExpData
out	*temp_data*	pointer to the temporary data struct<br>**Type**: TempData

Returns

void

Definition at line 1166 of file amici.c.

Here is the caller graph for this function:

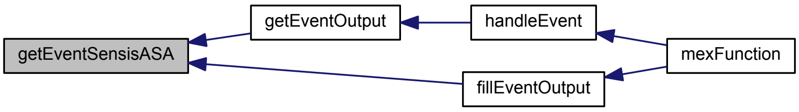

8.4.2.12 void getEventSigma (int ∗ *status*, int *ie*, int *iz*, void ∗ *ami_mem*, void ∗ *user_data*, void ∗ *return_data*, void ∗ *exp_data*, void ∗ *temp_data*)

getEventSigma extracts fills sigma_z either from the user defined function or from user input

Parameters

out	*status*	flag indicating success of execution<br>**Type**: ∗int
in	*ie*	event type index<br>**Type**: int
in	*iz*	event output index<br>**Type**: int
in	*ami_mem*	pointer to the solver memory block<br>**Type**: ∗void
in	*user_data*	pointer to the user data struct<br>**Type**: UserData
out	*return_data*	pointer to the return data struct<br>**Type**: ReturnData

in	*exp_data*	pointer to the experimental data struct<br>**Type**: ExpData
out	*temp_data*	pointer to the temporary data struct<br>**Type**: TempData

Returns

void

Definition at line 1236 of file amici.c.

Here is the caller graph for this function:

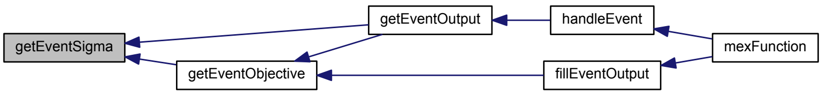

8.4.2.13 void getEventObjective (int ∗ *status*, int *ie*, void ∗ *ami_mem*, void ∗ *user_data*, void ∗ *return_data*, void ∗ *exp_data*, void ∗ *temp_data*)

getEventObjective updates the objective function on the occurence of an event

Parameters

out	*status*	flag indicating success of execution<br>**Type**: ∗int
in	*ie*	event type index<br>**Type**: int
in	*ami_mem*	pointer to the solver memory block<br>**Type**: ∗void
in	*user_data*	pointer to the user data struct<br>**Type**: UserData
out	*return_data*	pointer to the return data struct<br>**Type**: ReturnData
in	*exp_data*	pointer to the experimental data struct<br>**Type**: ExpData
out	*temp_data*	pointer to the temporary data struct<br>**Type**: TempData

Returns

void

Definition at line 1275 of file amici.c.

Here is the call graph for this function:

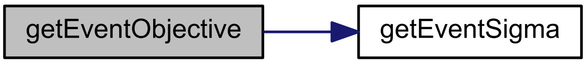

Here is the caller graph for this function:

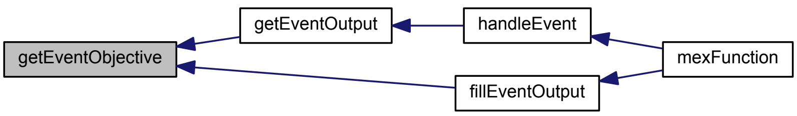

8.4.2.14 void getEventOutput (int ∗ *status*, realtype ∗ *tlastroot*, void ∗ *ami_mem*, void ∗ *user_data*, void ∗ *return_data*, void ∗ *exp_data*, void ∗ *temp_data*)

getEventOutput extracts output information for events

Parameters

out	*status*	flag indicating success of execution<br>**Type**: ∗int
in	*tlastroot*	timepoint of last occured event<br>**Type**: ∗realtype
in	*ami_mem*	pointer to the solver memory block<br>**Type**: ∗void
in	*user_data*	pointer to the user data struct<br>**Type**: UserData
out	*return_data*	pointer to the return data struct<br>**Type**: ReturnData
in	*exp_data*	pointer to the experimental data struct<br>**Type**: ExpData
out	*temp_data*	pointer to the temporary data struct<br>**Type**: TempData

Returns

void

Definition at line 1317 of file amici.c.

Here is the call graph for this function:

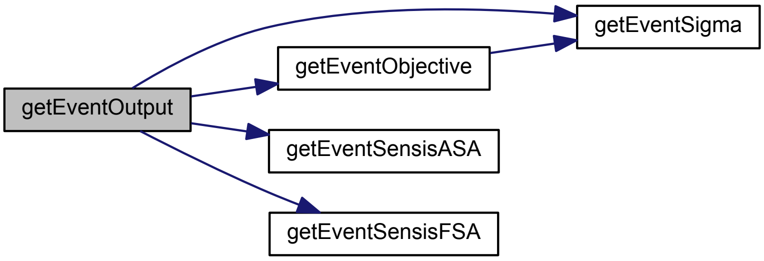

Here is the caller graph for this function:

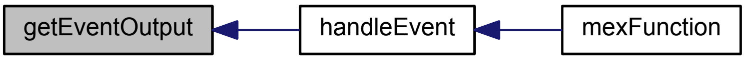

8.4.2.15 void fillEventOutput (int ∗ *status*, void ∗ *ami_mem*, void ∗ *user_data*, void ∗ *return_data*, void ∗ *exp_data*, void ∗ *temp_data*)

fillEventOutput fills missing roots at last timepoint

Parameters

out	*status*	flag indicating success of execution<br>**Type**: ∗int
in	*ami_mem*	pointer to the solver memory block<br>**Type**: ∗void
in	*user_data*	pointer to the user data struct<br>**Type**: UserData
out	*return_data*	pointer to the return data struct<br>**Type**: ReturnData
in	*exp_data*	pointer to the experimental data struct<br>**Type**: ExpData
out	*temp_data*	pointer to the temporary data struct<br>**Type**: TempData

Returns

void

Definition at line 1387 of file amici.c.

Here is the call graph for this function:

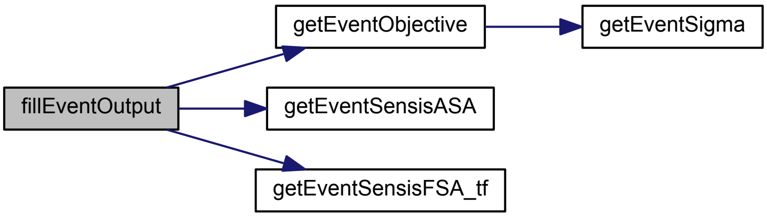

Here is the caller graph for this function:

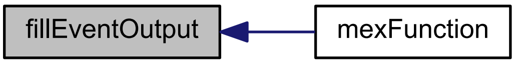

8.4.2.16 void handleDataPoint (int ∗ *status*, int *it*, void ∗ *ami_mem*, void ∗ *user_data*, void ∗ *return_data*, void ∗ *exp_data*, void ∗ *temp_data*)

handleDataPoint executes everything necessary for the handling of data points

Parameters

out	*status*	flag indicating success of execution<br>**Type**: ∗int
in	*it*	index of data point<br>**Type**: int
in	*ami_mem*	pointer to the solver memory block<br>**Type**: ∗void
in	*user_data*	pointer to the user data struct<br>**Type**: UserData
out	*return_data*	pointer to the return data struct<br>**Type**: ReturnData
in	*exp_data*	pointer to the experimental data struct<br>**Type**: ExpData
out	*temp_data*	pointer to the temporary data struct<br>**Type**: TempData

Returns

void

Definition at line 1439 of file amici.c.

Here is the call graph for this function:

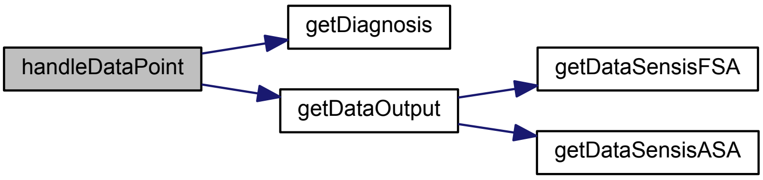

Here is the caller graph for this function:

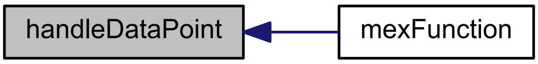

8.4.2.17 void handleDataPointB (int ∗ *status*, int *it*, void ∗ *ami_mem*, void ∗ *user_data*, void ∗ *return_data*, void ∗ *temp_data*)

handleDataPoint executes everything necessary for the handling of data points for the backward problems

Parameters

out	*status*	flag indicating success of execution<br>**Type**: ∗int
in	*it*	index of data point<br>**Type**: int
in	*ami_mem*	pointer to the solver memory block<br>**Type**: ∗void
in	*user_data*	pointer to the user data struct<br>**Type**: UserData
out	*return_data*	pointer to the return data struct<br>**Type**: ReturnData
out	*temp_data*	pointer to the temporary data struct<br>**Type**: TempData

Returns

void

Definition at line 1494 of file amici.c.

Here is the call graph for this function:

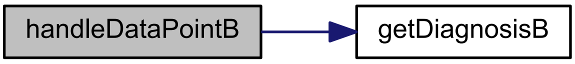

Here is the caller graph for this function:

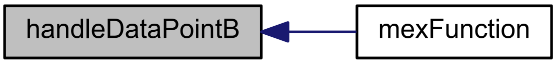

8.4.2.18 void handleEvent (int * *status,* int *iroot,* realtype * *tlastroot,* void * *ami_mem,* void * *user_data,* void * *return_data,* void * *exp_data,* void * *temp_data*)

handleEvent executes everything necessary for the handling of events

Parameters

out	*status*	flag indicating success of execution<br>**Type**: *int
out	*iroot*	index of event<br>**Type**: int
out	*tlastroot*	pointer to the timepoint of the last event<br>**Type**: *realtype
in	*ami_mem*	pointer to the solver memory block<br>**Type**: *void
in	*user_data*	pointer to the user data struct<br>**Type**: UserData
out	*return_data*	pointer to the return data struct<br>**Type**: ReturnData
in	*exp_data*	pointer to the experimental data struct<br>**Type**: ExpData
out	*temp_data*	pointer to the temporary data struct<br>**Type**: TempData

Returns

void

Definition at line 1525 of file amici.c.

Here is the call graph for this function:

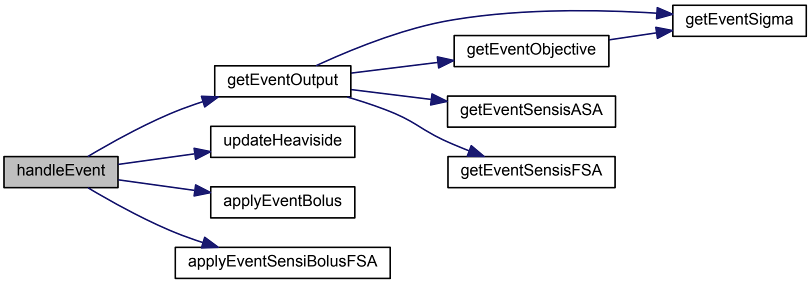

Here is the caller graph for this function:

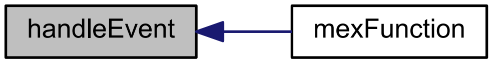

8.4.2.19 void handleEventB (int * *status*, int *iroot*, void * *ami_mem*, void * *user_data*, void * *temp_data*)

handleEventB executes everything necessary for the handling of events for the backward problem

Parameters

out	*status*	flag indicating success of execution<br>**Type**: *int
out	*iroot*	index of event<br>**Type**: int
in	*ami_mem*	pointer to the solver memory block<br>**Type**: *void
in	*user_data*	pointer to the user data struct<br>**Type**: UserData
out	*temp_data*	pointer to the temporary data struct<br>**Type**: TempData

Returns

cv_status updated status flag
Type: int

Definition at line 1626 of file amici.c.

Here is the call graph for this function:

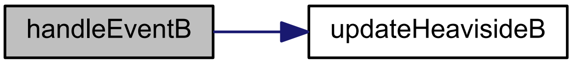

Here is the caller graph for this function:

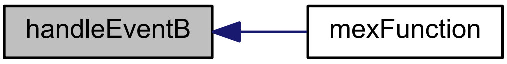

8.4.2.20 realtype getTnext (realtype * *troot*, int *iroot*, realtype * *tdata*, int *it*, void * *user_data*)

getTnext computes the next timepoint to integrate to. This is the maximum of tdata and troot but also takes into account if it<0 or iroot<0 where these expressions do not necessarily make sense

Parameters

in	*troot*	timepoint of next event<br>**Type**: realtype
in	*iroot*	index of next event<br>**Type**: int
in	*tdata*	timepoint of next data point<br>**Type**: realtype
in	*it*	index of next data point<br>**Type**: int
in	*user_data*	pointer to the user data struct<br>**Type**: UserData

Returns

tnext next timepoint
Type: realtype

Definition at line 1684 of file amici.c.

Here is the caller graph for this function:

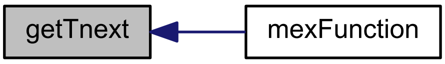

8.4.2.21 void applyEventBolus (int * *status*, void * *ami_mem*, void * *user_data*, void * *temp_data*)

applyEventBolus applies the event bolus to the current state

Parameters

out	*status*	flag indicating success of execution<br>**Type**: ∗int
in	*ami_mem*	pointer to the solver memory block<br>**Type**: ∗void
in	*user_data*	pointer to the user data struct<br>**Type**: UserData
out	*temp_data*	pointer to the temporary data struct<br>**Type**: TempData

Returns

void

Definition at line 1729 of file amici.c.

Here is the caller graph for this function:

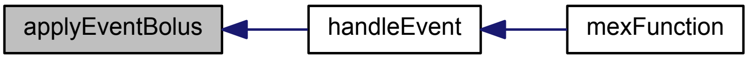

8.4.2.22 void applyEventSensiBolusFSA (int ∗ *status*, void ∗ *ami_mem*, void ∗ *user_data*, void ∗ *temp_data*)

applyEventSensiBolusFSA applies the event bolus to the current sensitivities

Parameters

out	*status*	flag indicating success of execution<br>**Type**: ∗int
in	*ami_mem*	pointer to the solver memory block<br>**Type**: ∗void
in	*user_data*	pointer to the user data struct<br>**Type**: UserData
out	*temp_data*	pointer to the temporary data struct<br>**Type**: TempData

Returns

void

Definition at line 1764 of file amici.c.

Here is the caller graph for this function:

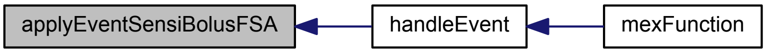

8.4.2.23 void initHeaviside (int * *status*, void * *user_data*, void * *temp_data*)

initHeaviside initialises the heaviside variables h at the intial time t0 heaviside variables activate/deactivate on event occurences

Parameters

out	*status*	flag indicating success of execution<br>**Type**: ∗int
in	*user_data*	pointer to the user data struct<br>**Type**: UserData
out	*temp_data*	pointer to the temporary data struct<br>**Type**: TempData

Returns

void

Definition at line 1802 of file amici.c.

Here is the caller graph for this function:

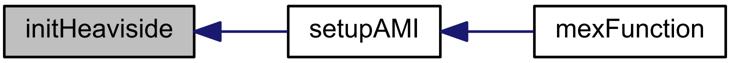

8.4.2.24 void updateHeaviside (int ∗ *status,* void ∗ *user_data,* void ∗ *temp_data*)

updateHeaviside updates the heaviside variables h on event occurences

Parameters

out	*status*	flag indicating success of execution<br>**Type**: ∗int
in	*user_data*	pointer to the user data struct<br>**Type**: UserData
out	*temp_data*	pointer to the temporary data struct<br>**Type**: TempData

Returns

void

Definition at line 1835 of file amici.c.

Here is the caller graph for this function:

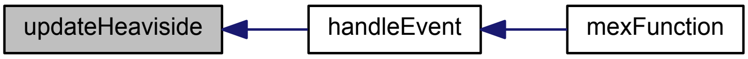

8.4.2.25 void updateHeavisideB (int ∗ *status,* int *iroot,* void ∗ *user_data,* void ∗ *temp_data*)

updateHeavisideB updates the heaviside variables h on event occurences for the backward problem

Parameters

out	*status*	flag indicating success of execution<br>**Type**: ∗int
in	*iroot*	discontinuity occurance index<br>**Type**: int
in	*user_data*	pointer to the user data struct<br>**Type**: UserData
out	*temp_data*	pointer to the temporary data struct<br>**Type**: TempData

Returns

void

Definition at line 1864 of file amici.c.

Here is the caller graph for this function:

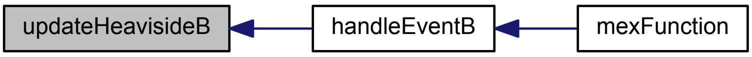

8.4.2.26 void getDiagnosis (int ∗ *status,* int *it,* void ∗ *ami_mem,* void ∗ *user_data,* void ∗ *return_data*)

getDiagnosis extracts diagnosis information from solver memory block and writes them into the return data struct

Parameters

out	*status*	flag indicating success of execution<br>**Type**: ∗int
in	*it*	time-point index<br>**Type**: int
in	*ami_mem*	pointer to the solver memory block<br>**Type**: ∗void
in	*user_data*	pointer to the user data struct<br>**Type**: UserData
out	*return_data*	pointer to the return data struct<br>**Type**: ReturnData

Returns

void

Definition at line 1894 of file amici.c.

Here is the caller graph for this function:

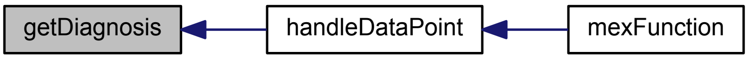

8.4.2.27 void getDiagnosisB (int * *status*, int *it*, void * *ami_mem*, void * *user_data*, void * *return_data*, void * *temp_data*)

getDiagnosisB extracts diagnosis information from solver memory block and writes them into the return data struct for the backward problem

Parameters

out	*status*	flag indicating success of execution<br>**Type**: *int
in	*it*	time-point index<br>**Type**: int
in	*ami_mem*	pointer to the solver memory block<br>**Type**: *void
in	*user_data*	pointer to the user data struct<br>**Type**: UserData
out	*return_data*	pointer to the return data struct<br>**Type**: ReturnData
out	*temp_data*	pointer to the temporary data struct<br>**Type**: TempData

Returns

void

Definition at line 1932 of file amici.c.

Here is the caller graph for this function:

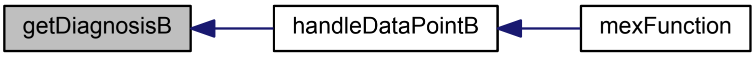

8.5 src/spline.c File Reference

definition of spline functions

This graph shows which files directly or indirectly include this file:

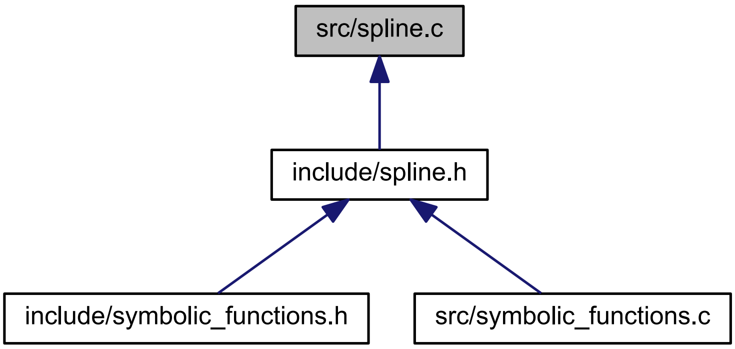

Functions

- static int spline (int n, int end1, int end2, double slope1, double slope2, double x[ ], double y[ ], double b[ ], double c[ ], double d[ ])
- static double seval (int n, double u, double x[ ], double y[ ], double b[ ], double c[ ], double d[ ])
- static double deriv (int n, double u, double x[ ], double b[ ], double c[ ], double d[ ])
- static double sinteg (int n, double u, double x[ ], double y[ ], double b[ ], double c[ ], double d[ ])

8.5.1 Detailed Description

Author

Peter & Nigel, Design Software, 42 Gubberley St, Kenmore, 4069, Australia.

8.5.2 Function Documentation

8.5.2.1 static int spline (int *n,* int *end1,* int *end2,* double *slope1,* double *slope2,* double *x[ ],* double *y[ ],* double *b[ ],* double *c[ ],* double *d[ ]*) [static]

Evaluate the coefficients b[i], c[i], d[i], i = 0, 1, .. n-1 for a cubic interpolating spline

S(xx) = Y[i] + b[i] ∗ w + c[i] ∗ w∗∗2 + d[i] ∗ w∗∗3 where w = xx - x[i] and x[i] <= xx <= x[i+1]

The n supplied data points are x[i], y[i], i = 0 ... n-1.

Parameters

in	*n*	The number of data points or knots (n >= 2)
in	*end1*	0: default condition 1: specify the slopes at x[0]
in	*end2*	0: default condition 1: specify the slopes at x[n-1]
in	*slope1*	slope at x[0]
in	*slope2*	slope at x[n-1]
in	*x[ ]*	the abscissas of the knots in strictly increasing order

in	*y[ ]*	the ordinates of the knots
out	*b[ ]*	array of spline coefficients
out	*c[ ]*	array of spline coefficients
out	*d[ ]*	array of spline coefficients

Return values

0	normal return
1	less than two data points; cannot interpolate
2	x[] are not in ascending order

Notes

- The accompanying function seval() may be used to evaluate the spline while deriv will provide the first derivative.

- Using p to denote differentiation y[i] = S(X[i]) b[i] = Sp(X[i]) c[i] = Spp(X[i])/2 d[i] = Sppp(X[i])/6 (Derivative from the right)

- Since the zero elements of the arrays ARE NOW used here, all arrays to be passed from the main program should be dimensioned at least [n]. These routines will use elements [0 .. n-1].

- Adapted from the text Forsythe, G.E., Malcolm, M.A. and Moler, C.B. (1977) "Computer Methods for Mathematical Computations" Prentice Hall

- Note that although there are only n-1 polynomial segments, n elements are requird in b, c, d. The elements b[n-1], c[n-1] and d[n-1] are set to continue the last segment past x[n-1].

Definition at line 66 of file spline.c.

Here is the caller graph for this function:

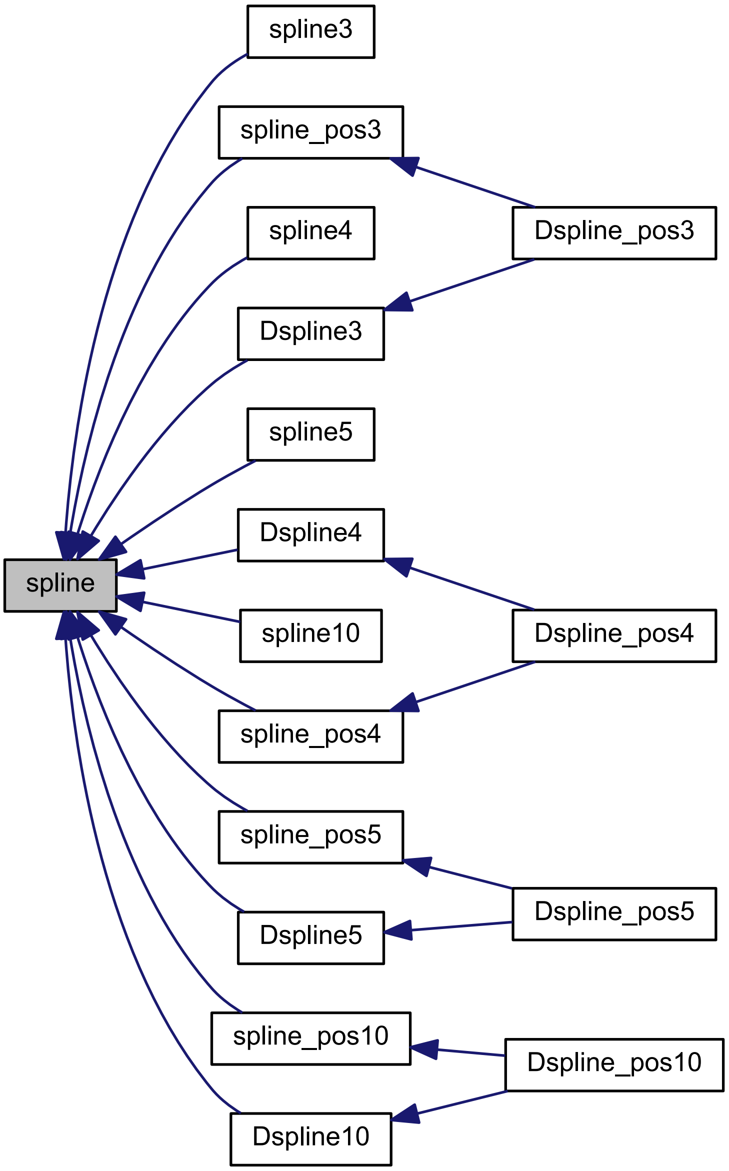

8.5.2.2 static double seval (int *n*, double *u*, double *x[ ]*, double *y[ ]*, double *b[ ]*, double *c[ ]*, double *d[ ]*) `[static]`

Evaluate the cubic spline function

S(xx) = y[i] + b[i] ∗ w + c[i] ∗ w∗∗2 + d[i] ∗ w∗∗3 where w = u - x[i] and x[i] <= u <= x[i+1] Note that Horner's rule is used. If u < x[0] then i = 0 is used. If u > x[n-1] then i = n-1 is used.

Parameters

`in`	*n*	The number of data points or knots (n >= 2)
`in`	*u*	the abscissa at which the spline is to be evaluated
`in`	*x[ ]*	the abscissas of the knots in strictly increasing order
`in`	*y[ ]*	the ordinates of the knots
`in`	*b*	array of spline coefficients computed by spline().
`in`	*c*	array of spline coefficients computed by spline().
`in`	*d*	array of spline coefficients computed by spline().

Returns

the value of the spline function at u

Notes

- If u is not in the same interval as the previous call then a binary search is performed to determine the proper interval.

Definition at line 208 of file spline.c.

Here is the caller graph for this function:

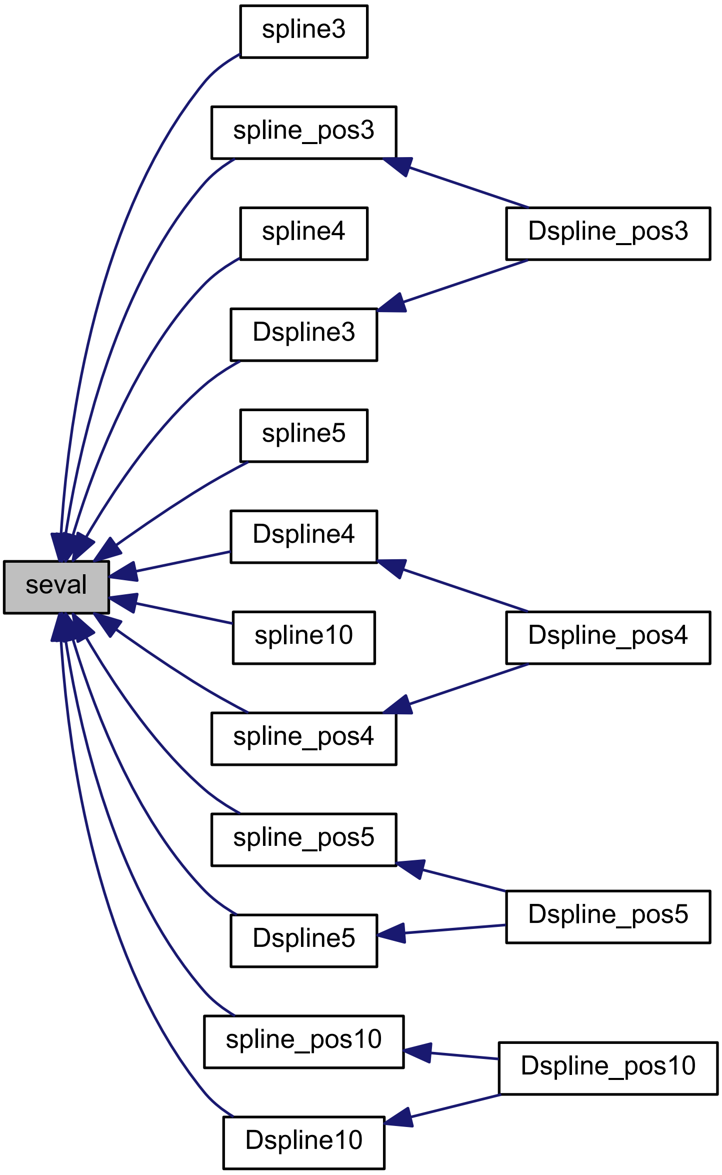

8.5.2.3 static double deriv (int *n*, double *u*, double *x[ ]*, double *b[ ]*, double *c[ ]*, double *d[ ]*) [static]

Evaluate the derivative of the cubic spline function

S(x) = B[i] + 2.0 ∗ C[i] ∗ w + 3.0 ∗ D[i] ∗ w∗∗2 where w = u - X[i] and X[i] <= u <= X[i+1] Note that Horner's rule is used. If U < X[0] then i = 0 is used. If U > X[n-1] then i = n-1 is used.

Parameters

in	*n*	the number of data points or knots (n >= 2)
in	*u*	the abscissa at which the derivative is to be evaluated
in	*x*	the abscissas of the knots in strictly increasing order
in	*b*	array of spline coefficients computed by spline()
in	*c*	array of spline coefficients computed by spline()
in	*d*	array of spline coefficients computed by spline()

Returns

the value of the derivative of the spline function at u

Notes

- If u is not in the same interval as the previous call then a binary search is performed to determine the proper interval.

Definition at line 264 of file spline.c.

8.5.2.4 static double sinteg (int *n*, double *u*, double *x[ ]*, double *y[ ]*, double *b[ ]*, double *c[ ]*, double *d[ ]*) [static]

Integrate the cubic spline function

S(xx) = y[i] + b[i] * w + c[i] * w**2 + d[i] * w**3 where w = u - x[i] and x[i] <= u <= x[i+1]

The integral is zero at u = x[0].

If u < x[0] then i = 0 segment is extrapolated. If u > x[n-1] then i = n-1 segment is extrapolated.

Parameters

in	*n*	the number of data points or knots (n >= 2)
in	*u*	the abscissa at which the spline is to be evaluated
in	*x[ ]*	the abscissas of the knots in strictly increasing order
in	*y[ ]*	the ordinates of the knots
in	*b*	array of spline coefficients computed by spline().
in	*c*	array of spline coefficients computed by spline().
in	*d*	array of spline coefficients computed by spline().

Returns

the value of the spline function at u

Notes

- If u is not in the same interval as the previous call then a binary search is performed to determine the proper interval.

Definition at line 324 of file spline.c.

8.6 src/symbolic_functions.c File Reference

definition of symbolic functions

```
#include <math.h>
#include <mex.h>
#include <float.h>
#include <include/spline.h>
```

Include dependency graph for symbolic_functions.c:

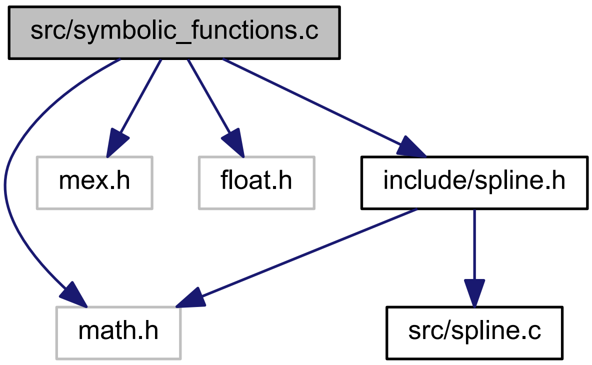

Macros

- #define TRUE 1
- #define FALSE 0

Functions

- double amilog (double x)
- double heaviside (double x)
- double sign (double x)
- double am_min (double a, double b)
- double Dam_min (int id, double a, double b)
- double am_max (double a, double b)
- double Dam_max (int id, double a, double b)
- double spline3 (double t, double t1, double p1, double t2, double p2, double t3, double p3, int ss, double dudt)
- double spline_pos3 (double t, double t1, double p1, double t2, double p2, double t3, double p3, int ss, double dudt)
- double spline4 (double t, double t1, double p1, double t2, double p2, double t3, double p3, double t4, double p4, int ss, double dudt)
- double spline_pos4 (double t, double t1, double p1, double t2, double p2, double t3, double p3, double t4, double p4, int ss, double dudt)
- double spline5 (double t, double t1, double p1, double t2, double p2, double t3, double p3, double t4, double p4, double t5, double p5, int ss, double dudt)
- double spline_pos5 (double t, double t1, double p1, double t2, double p2, double t3, double p3, double t4, double p4, double t5, double p5, int ss, double dudt)
- double spline10 (double t, double t1, double p1, double t2, double p2, double t3, double p3, double t4, double p4, double t5, double p5, double t6, double p6, double t7, double p7, double t8, double p8, double t9, double p9, double t10, double p10, int ss, double dudt)
- double spline_pos10 (double t, double t1, double p1, double t2, double p2, double t3, double p3, double t4, double p4, double t5, double p5, double t6, double p6, double t7, double p7, double t8, double p8, double t9, double p9, double t10, double p10, int ss, double dudt)
- double Dspline3 (int id, double t, double t1, double p1, double t2, double p2, double t3, double p3, int ss, double dudt)

- double Dspline_pos3 (int id, double t, double t1, double p1, double t2, double p2, double t3, double p3, int ss, double dudt)
- double Dspline4 (int id, double t, double t1, double p1, double t2, double p2, double t3, double p3, double t4, double p4, int ss, double dudt)
- double Dspline_pos4 (int id, double t, double t1, double p1, double t2, double p2, double t3, double p3, double t4, double p4, int ss, double dudt)
- double Dspline5 (int id, double t, double t1, double p1, double t2, double p2, double t3, double p3, double t4, double p4, double t5, double p5, int ss, double dudt)
- double Dspline_pos5 (int id, double t, double t1, double p1, double t2, double p2, double t3, double p3, double t4, double p4, double t5, double p5, int ss, double dudt)
- double Dspline10 (int id, double t, double t1, double p1, double t2, double p2, double t3, double p3, double t4, double p4, double t5, double p5, double t6, double p6, double t7, double p7, double t8, double p8, double t9, double p9, double t10, double p10, int ss, double dudt)
- double Dspline_pos10 (int id, double t, double t1, double p1, double t2, double p2, double t3, double p3, double t4, double p4, double t5, double p5, double t6, double p6, double t7, double p7, double t8, double p8, double t9, double p9, double t10, double p10, int ss, double dudt)

8.6.1 Detailed Description

This file contains definitions of various symbolic functions which

8.6.2 Macro Definition Documentation

8.6.2.1 #define TRUE 1

bool return value true

Definition at line 16 of file symbolic_functions.c.

8.6.2.2 #define FALSE 0

bool return value false

Definition at line 18 of file symbolic_functions.c.

8.6.3 Function Documentation

8.6.3.1 double amilog (double *x*)

c implementation of log function, this prevents returning NaN values for negative values

Parameters

x	argument

Returns

if(x>0) then log(x) else -Inf

Definition at line 28 of file symbolic_functions.c.

8.6.3.2 double heaviside (double *x*)

c implementation of matlab function heaviside

Parameters

x	argument

Returns

if(x>0) then 1 else 0

Definition at line 43 of file symbolic_functions.c.

Here is the caller graph for this function:

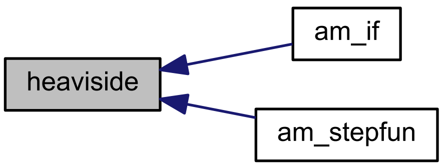

8.6.3.3 double sign (double *x*)

c implementation of matlab function sign

Parameters

x	argument

Returns

0
Type: double

Definition at line 59 of file symbolic_functions.c.

8.6.3.4 double am_min (double *a*, double *b*)

c implementation of matlab function min

Parameters

a	value1<br>**Type**: double
b	value2<br>**Type**: double

Returns

if(a $<$ b) then a else b
Type: double

Definition at line 79 of file symbolic_functions.c.

8.6.3.5 double Dam_min (int *id*, double *a*, double *b*)

parameter derivative of c implementation of matlab function min

Parameters

id	argument index for differentiation
a	bool1<br>**Type**: double
b	bool2<br>**Type**: double

Returns

id == 1: if(a $<$ b) then 1 else 0
Type: double
id == 2: if(a $<$ b) then 0 else 1
Type: double

Definition at line 93 of file symbolic_functions.c.

8.6.3.6 double am_max (double *a*, double *b*)

c implementation of matlab function max

Parameters

a	value1<br>**Type**: double
b	value2<br>**Type**: double

Returns

if(a $>$ b) then a else b
Type: double

Definition at line 117 of file symbolic_functions.c.

8.6.3.7 double Dam_max (int *id*, double *a*, double *b*)

parameter derivative of c implementation of matlab function max

Parameters

id	argument index for differentiation
a	bool1<br>**Type**: double
b	bool2<br>**Type**: double

Returns

id == 1: if(a $>$ b) then 1 else 0
Type: double
id == 2: if(a $>$ b) then 0 else 1
Type: double

Definition at line 131 of file symbolic_functions.c.

8.6.3.8 double spline3 (double *t*, double *t1*, double *p1*, double *t2*, double *p2*, double *t3*, double *p3*, int *ss*, double *dudt*)

spline function with 3 nodes

Parameters

t	point at which the spline should be evaluated
t1	location of node 1
p1	spline value at node 1
t2	location of node 2
p2	spline value at node 2
t3	location of node 3
p3	spline value at node 3
ss	flag indicating whether slope at first node should be user defined
dudt	user defined slope at first node

Returns

spline(t)

Definition at line 164 of file symbolic_functions.c.

Here is the call graph for this function:

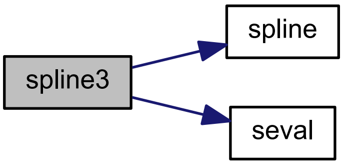

8.6.3.9 double spline_pos3 (double *t*, double *t1*, double *p1*, double *t2*, double *p2*, double *t3*, double *p3*, int *ss*, double *dudt*)

positive spline function with 3 nodes

Parameters

t	point at which the spline should be evaluated
t1	location of node 1
p1	spline value at node 1
t2	location of node 2
p2	spline value at node 2
t3	location of node 3
p3	spline value at node 3
ss	flag indicating whether slope at first node should be user defined
dudt	user defined slope at first node

Returns

spline(t)

Definition at line 205 of file symbolic_functions.c.

Here is the call graph for this function:

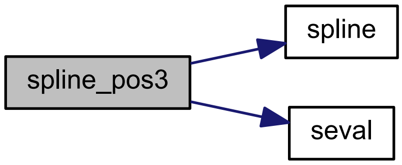

Here is the caller graph for this function:

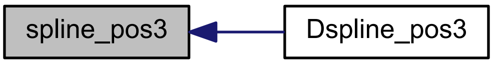

8.6.3.10 double spline4 (double *t*, double *t1*, double *p1*, double *t2*, double *p2*, double *t3*, double *p3*, double *t4*, double *p4*, int *ss*, double *dudt*)

spline function with 4 nodes

Parameters

t	point at which the spline should be evaluated
t1	location of node 1
p1	spline value at node 1
t2	location of node 2
p2	spline value at node 2
t3	location of node 3
p3	spline value at node 3
t4	location of node 4
p4	spline value at node 4
ss	flag indicating whether slope at first node should be user defined
dudt	user defined slope at first node

Returns

spline(t)

Definition at line 253 of file symbolic_functions.c.

Here is the call graph for this function:

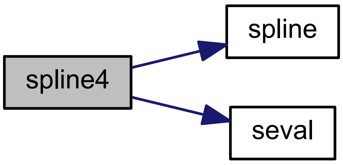

8.6.3.11 double spline_pos4 (double *t*, double *t1*, double *p1*, double *t2*, double *p2*, double *t3*, double *p3*, double *t4*, double *p4*, int *ss*, double *dudt*)

positive spline function with 4 nodes

Parameters

t	point at which the spline should be evaluated
t1	location of node 1
p1	spline value at node 1
t2	location of node 2
p2	spline value at node 2
t3	location of node 3
p3	spline value at node 3
t4	location of node 4
p4	spline value at node 4
ss	flag indicating whether slope at first node should be user defined
dudt	user defined slope at first node

Returns

spline(t)

Definition at line 297 of file symbolic_functions.c.

Here is the call graph for this function:

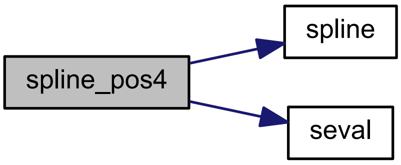

Here is the caller graph for this function:

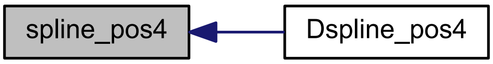

8.6.3.12 double spline5 (double *t*, double *t1*, double *p1*, double *t2*, double *p2*, double *t3*, double *p3*, double *t4*, double *p4*, double *t5*, double *p5*, int *ss*, double *dudt*)

spline function with 5 nodes

Parameters

t	point at which the spline should be evaluated
t1	location of node 1
p1	spline value at node 1
t2	location of node 2
p2	spline value at node 2
t3	location of node 3
p3	spline value at node 3
t4	location of node 4
p4	spline value at node 4
t5	location of node 5
p5	spline value at node 5
ss	flag indicating whether slope at first node should be user defined
dudt	user defined slope at first node

Returns

spline(t)

Definition at line 349 of file symbolic_functions.c.

Here is the call graph for this function:

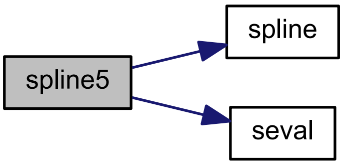

8.6.3.13 double spline_pos5 (double *t*, double *t1*, double *p1*, double *t2*, double *p2*, double *t3*, double *p3*, double *t4*, double *p4*, double *t5*, double *p5*, int *ss*, double *dudt*)

positive spline function with 5 nodes

Parameters

t	point at which the spline should be evaluated
t1	location of node 1
p1	spline value at node 1
t2	location of node 2
p2	spline value at node 2
t3	location of node 3
p3	spline value at node 3
t4	location of node 4
p4	spline value at node 4
t5	location of node 5
p5	spline value at node 5
ss	flag indicating whether slope at first node should be user defined
dudt	user defined slope at first node

Returns

spline(t)

Definition at line 397 of file symbolic_functions.c.

Here is the call graph for this function:

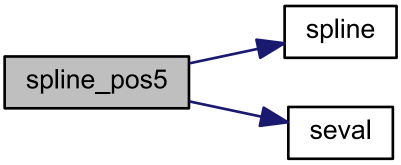

Here is the caller graph for this function:

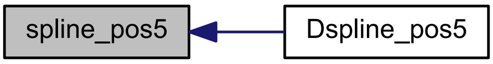

8.6.3.14 double spline10 (double *t*, double *t1*, double *p1*, double *t2*, double *p2*, double *t3*, double *p3*, double *t4*, double *p4*, double *t5*, double *p5*, double *t6*, double *p6*, double *t7*, double *p7*, double *t8*, double *p8*, double *t9*, double *p9*, double *t10*, double *p10*, int *ss*, double *dudt*)

spline function with 10 nodes

Parameters

t	point at which the spline should be evaluated
t1	location of node 1
p1	spline value at node 1
t2	location of node 2
p2	spline value at node 2
t3	location of node 3
p3	spline value at node 3
t4	location of node 4
p4	spline value at node 4
t5	location of node 5
p5	spline value at node 5
t6	location of node 6
p6	spline value at node 6
t7	location of node 7
p7	spline value at node 7
t8	location of node 8
p8	spline value at node 8
t9	location of node 9
p9	spline value at node 9
t10	location of node 10
p10	spline value at node 10
ss	flag indicating whether slope at first node should be user defined
dudt	user defined slope at first node

Returns

spline(t)

Definition at line 461 of file symbolic_functions.c.

Here is the call graph for this function:

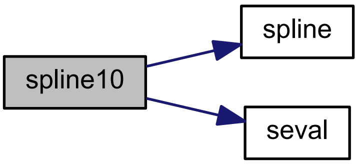

8.6.3.15 double spline_pos10 (double *t*, double *t1*, double *p1*, double *t2*, double *p2*, double *t3*, double *p3*, double *t4*, double *p4*, double *t5*, double *p5*, double *t6*, double *p6*, double *t7*, double *p7*, double *t8*, double *p8*, double *t9*, double *p9*, double *t10*, double *p10*, int *ss*, double *dudt*)

positive spline function with 10 nodes

Parameters

t	point at which the spline should be evaluated
t1	location of node 1
p1	spline value at node 1
t2	location of node 2
p2	spline value at node 2
t3	location of node 3
p3	spline value at node 3
t4	location of node 4
p4	spline value at node 4
t5	location of node 5
p5	spline value at node 5
t6	location of node 6
p6	spline value at node 6
t7	location of node 7
p7	spline value at node 7
t8	location of node 8
p8	spline value at node 8
t9	location of node 9
p9	spline value at node 9
t10	location of node 10
p10	spline value at node 10
ss	flag indicating whether slope at first node should be user defined
dudt	user defined slope at first node

Returns

spline(t)

Definition at line 529 of file symbolic_functions.c.

Here is the call graph for this function:

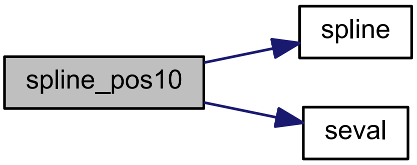

Here is the caller graph for this function:

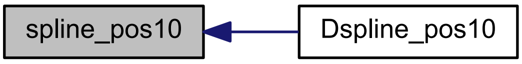

8.6.3.16 double Dspline3 (int *id*, double *t*, double *t1*, double *p1*, double *t2*, double *p2*, double *t3*, double *p3*, int *ss*, double *dudt*)

parameter derivative of spline function with 3 nodes

Parameters

id	argument index for differentiation
t	point at which the spline should be evaluated
t1	location of node 1
p1	spline value at node 1
t2	location of node 2
p2	spline value at node 2
t3	location of node 3
p3	spline value at node 3
ss	flag indicating whether slope at first node should be user defined
dudt	user defined slope at first node

Returns

dspline(t)dp(id)

Definition at line 590 of file symbolic_functions.c.

Here is the call graph for this function:

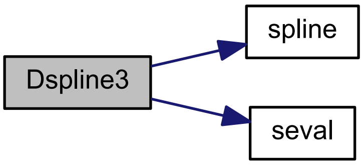

Here is the caller graph for this function:

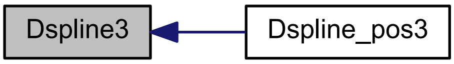

8.6.3.17 double Dspline_pos3 (int *id*, double *t*, double *t1*, double *p1*, double *t2*, double *p2*, double *t3*, double *p3*, int *ss*, double *dudt*)

parameter derivative of positive spline function with 3 nodes

Parameters

id	argument index for differentiation
t	point at which the spline should be evaluated
t1	location of node 1
p1	spline value at node 1
t2	location of node 2
p2	spline value at node 2
t3	location of node 3
p3	spline value at node 3
ss	flag indicating whether slope at first node should be user defined
dudt	user defined slope at first node

Returns

dspline(t)dp(id)

Definition at line 635 of file symbolic_functions.c.

Here is the call graph for this function:

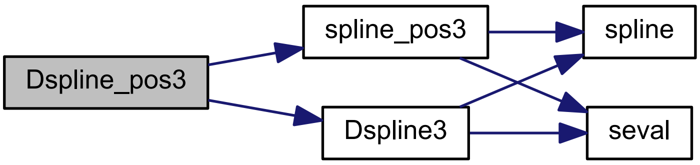

8.6.3.18 double Dspline4 (int *id*, double *t*, double *t1*, double *p1*, double *t2*, double *p2*, double *t3*, double *p3*, double *t4*, double *p4*, int *ss*, double *dudt*)

parameter derivative of spline function with 4 nodes

Parameters

id	argument index for differentiation
t	point at which the spline should be evaluated
t1	location of node 1
p1	spline value at node 1
t2	location of node 2
p2	spline value at node 2
t3	location of node 3
p3	spline value at node 3
t4	location of node 4
p4	spline value at node 4
ss	flag indicating whether slope at first node should be user defined

dudt	user defined slope at first node

Returns

dspline(t)dp(id)

Definition at line 678 of file symbolic_functions.c.

Here is the call graph for this function:

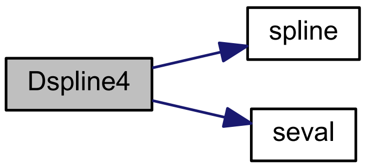

Here is the caller graph for this function:

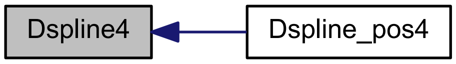

8.6.3.19 double Dspline_pos4 (int *id*, double *t*, double *t1*, double *p1*, double *t2*, double *p2*, double *t3*, double *p3*, double *t4*, double *p4*, int *ss*, double *dudt*)

parameter derivative of positive spline function with 4 nodes

Parameters

id	argument index for differentiation
t	point at which the spline should be evaluated
t1	location of node 1
p1	spline value at node 1
t2	location of node 2
p2	spline value at node 2
t3	location of node 3
p3	spline value at node 3
t4	location of node 4

p4	spline value at node 4
ss	flag indicating whether slope at first node should be user defined
dudt	user defined slope at first node

Returns

dspline(t)dp(id)

Definition at line 727 of file symbolic_functions.c.

Here is the call graph for this function:

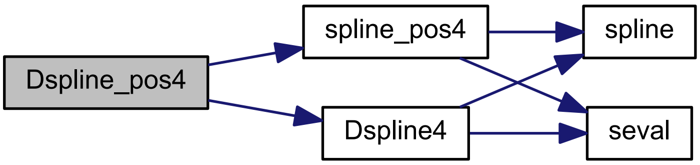

8.6.3.20 double Dspline5 (int *id*, double *t*, double *t1*, double *p1*, double *t2*, double *p2*, double *t3*, double *p3*, double *t4*, double *p4*, double *t5*, double *p5*, int *ss*, double *dudt*)

parameter derivative of spline function with 5 nodes

Parameters

id	argument index for differentiation
t	point at which the spline should be evaluated
t1	location of node 1
p1	spline value at node 1
t2	location of node 2
p2	spline value at node 2
t3	location of node 3
p3	spline value at node 3
t4	location of node 4
p4	spline value at node 4
t5	location of node 5
p5	spline value at node 5
ss	flag indicating whether slope at first node should be user defined
dudt	user defined slope at first node

Returns

dspline(t)dp(id)

Definition at line 772 of file symbolic_functions.c.

Here is the call graph for this function:

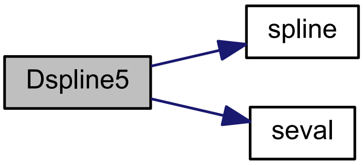

Here is the caller graph for this function:

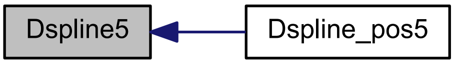

8.6.3.21 double Dspline_pos5 (int *id*, double *t*, double *t1*, double *p1*, double *t2*, double *p2*, double *t3*, double *p3*, double *t4*, double *p4*, double *t5*, double *p5*, int *ss*, double *dudt*)

parameter derivative of positive spline function with 5 nodes

Parameters

id	argument index for differentiation
t	point at which the spline should be evaluated
t1	location of node 1
p1	spline value at node 1
t2	location of node 2
p2	spline value at node 2
t3	location of node 3
p3	spline value at node 3
t4	location of node 4
p4	spline value at node 4
t5	location of node 5
p5	spline value at node 5

ss	flag indicating whether slope at first node should be user defined
dudt	user defined slope at first node

Returns

dspline(t)dp(id)

Definition at line 825 of file symbolic_functions.c.

Here is the call graph for this function:

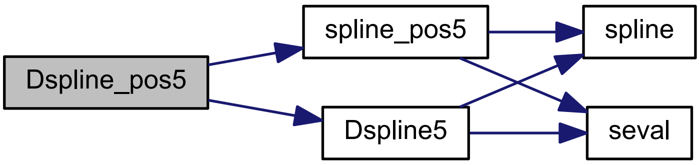

8.6.3.22 double Dspline10 (int *id*, double *t*, double *t1*, double *p1*, double *t2*, double *p2*, double *t3*, double *p3*, double *t4*, double *p4*, double *t5*, double *p5*, double *t6*, double *p6*, double *t7*, double *p7*, double *t8*, double *p8*, double *t9*, double *p9*, double *t10*, double *p10*, int *ss*, double *dudt*)

parameter derivative of spline function with 10 nodes

Parameters

id	argument index for differentiation
t	point at which the spline should be evaluated
t1	location of node 1
p1	spline value at node 1
t2	location of node 2
p2	spline value at node 2
t3	location of node 3
p3	spline value at node 3
t4	location of node 4
p4	spline value at node 4
t5	location of node 5
p5	spline value at node 5
t6	location of node 6
p6	spline value at node 6
t7	location of node 7
p7	spline value at node 7
t8	location of node 8
p8	spline value at node 8

t9	location of node 9
p9	spline value at node 9
t10	location of node 10
p10	spline value at node 10
ss	flag indicating whether slope at first node should be user defined
dudt	user defined slope at first node

Returns

dspline(t)dp(id)

Definition at line 881 of file symbolic_functions.c.

Here is the call graph for this function:

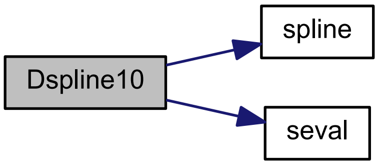

Here is the caller graph for this function:

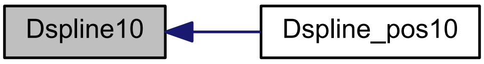

8.6.3.23 double Dspline_pos10 (int *id*, double *t*, double *t1*, double *p1*, double *t2*, double *p2*, double *t3*, double *p3*, double *t4*, double *p4*, double *t5*, double *p5*, double *t6*, double *p6*, double *t7*, double *p7*, double *t8*, double *p8*, double *t9*, double *p9*, double *t10*, double *p10*, int *ss*, double *dudt*)

parameter derivative of positive spline function with 10 nodes

Parameters

id	argument index for differentiation
t	point at which the spline should be evaluated
t1	location of node 1
p1	spline value at node 1

t2	location of node 2
p2	spline value at node 2
t3	location of node 3
p3	spline value at node 3
t4	location of node 4
p4	spline value at node 4
t5	location of node 5
p5	spline value at node 5
t6	location of node 6
p6	spline value at node 6
t7	location of node 7
p7	spline value at node 7
t8	location of node 8
p8	spline value at node 8
t9	location of node 9
p9	spline value at node 9
t10	location of node 10
p10	spline value at node 10
ss	flag indicating whether slope at first node should be user defined
dudt	user defined slope at first node

Returns

dspline(t)dp(id)

Definition at line 954 of file symbolic_functions.c.

Here is the call graph for this function:

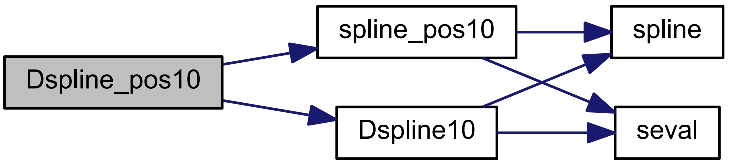

8.7 symbolic/am_and.m File Reference

syms x y f = symfun(sym(`cw_and(x,y)`),[x y]); fun = f(a,b);

Functions

- mlhsInnerSubst< matlabtypesubstitute > am_and (matlabtypesubstitute a, matlabtypesubstitute b)

  syms x y f = symfun(sym(`cw_and(x,y)`),[x y]); fun = f(a,b);

8.8 symbolic/am_ge.m File Reference

syms x y f = symfun(sym(`cw_ge(x,y)`),[x y]); fun = f(a,b);

Functions

- mlhsInnerSubst< matlabtypesubstitute > am_ge (matlabtypesubstitute a, matlabtypesubstitute b)
  syms x y f = symfun(sym(`cw_ge(x,y)`),[x y]); fun = f(a,b);

8.9 symbolic/am_gt.m File Reference

syms x y f = symfun(sym(`cw_gt(x,y)`),[x y]); fun = f(a,b);

Functions

- mlhsInnerSubst< matlabtypesubstitute > am_gt (matlabtypesubstitute a, matlabtypesubstitute b)
  syms x y f = symfun(sym(`cw_gt(x,y)`),[x y]); fun = f(a,b);

8.10 symbolic/am_if.m File Reference

syms x y z f = symfun(sym(`am_if(x,y,z)`),[x y z]); fun = f(condition, truepart, falsepart);

Functions

- mlhsInnerSubst< matlabtypesubstitute > am_if (matlabtypesubstitute condition, matlabtypesubstitute truepart, matlabtypesubstitute falsepart)
  syms x y z f = symfun(sym(`am_if(x,y,z)`),[x y z]); fun = f(condition, truepart, falsepart);

8.11 symbolic/am_le.m File Reference

syms x y f = symfun(sym(`cw_le(x,y)`),[x y]); fun = f(a,b);

Functions

- mlhsInnerSubst< matlabtypesubstitute > am_le (matlabtypesubstitute a, matlabtypesubstitute b)
  syms x y f = symfun(sym(`cw_le(x,y)`),[x y]); fun = f(a,b);

8.12 symbolic/am_lt.m File Reference

syms x y f = symfun(sym(`cw_lt(x,y)`),[x y]); fun = f(a,b);

Functions

- mlhsInnerSubst< matlabtypesubstitute > am_lt (matlabtypesubstitute a, matlabtypesubstitute b)
  syms x y f = symfun(sym(`cw_lt(x,y)`),[x y]); fun = f(a,b);

8.13 symbolic/am_max.m File Reference

syms x y f = symfun(sym(`am_max(x,y)`),[x y]); fun = f(a,b);

Functions

- mlhsInnerSubst< matlabtypesubstitute > am_max (matlabtypesubstitute a, matlabtypesubstitute b)
  syms x y f = symfun(sym(`am_max(x,y)`),[x y]); fun = f(a,b);

8.14 symbolic/am_min.m File Reference

syms x y f = symfun(sym(`am_min(x,y)`),[x y]); fun = f(a,b);

Functions

- mlhsInnerSubst< matlabtypesubstitute > am_min (matlabtypesubstitute a, matlabtypesubstitute b)

    syms x y f = symfun(sym(`am_min(x,y)`),[x y]); fun = f(a,b);

8.15 symbolic/am_or.m File Reference

syms x y f = symfun(sym(`cw_or(x,y)`),[x y]); fun = f(a,b);

Functions

- mlhsInnerSubst< matlabtypesubstitute > am_or (matlabtypesubstitute a, matlabtypesubstitute b)

    syms x y f = symfun(sym(`cw_or(x,y)`),[x y]); fun = f(a,b);

8.16 symbolic/am_stepfun.m File Reference

syms x y f = symfun(sym(`am_min(x,y)`),[x y]); fun = f(a,b);

Functions

- mlhsInnerSubst< matlabtypesubstitute > am_stepfun (matlabtypesubstitute t, matlabtypesubstitute tstart, matlabtypesubstitute vstart, matlabtypesubstitute tend, matlabtypesubstitute vend)

    syms x y f = symfun(sym(`am_min(x,y)`),[x y]); fun = f(a,b);

Index

AMI_SUCCESS
amici.c, 99
adjoint
amimodel, 59
am_J
UserData, 92
am_Jdata
ReturnData, 73
am_Jtmp
TempData, 83
am_M
UserData, 92
am_atol
UserData, 90
am_bsx0
UserData, 91
am_chi2data
ReturnData, 75
am_deltaqB
TempData, 86
am_deltasx
TempData, 86
am_deltax
TempData, 86
am_deltaxB
TempData, 86
am_dfdx
UserData, 92
am_dgdp
TempData, 83
am_dgdx
TempData, 83
am_discs
TempData, 86
am_drdp
TempData, 83
am_drdx
TempData, 83
am_drvaldp
TempData, 84
am_drvaldx
TempData, 84
am_dsigma_ydp
TempData, 84
am_dsigma_zdp
TempData, 85
am_dwdp
UserData, 92
am_dwdx
UserData, 92
am_dx
TempData, 82
am_dx_old
TempData, 82
am_dx_tmp
TempData, 85
am_dxB
TempData, 82
am_dxB_tmp
TempData, 85
am_dxdotdp
UserData, 92
am_dxdotdpdata
ReturnData, 73
am_dydp
TempData, 84
am_dydpdata
ReturnData, 73
am_dydx
TempData, 84
am_dydxdata
ReturnData, 73
am_dzdp
TempData, 84
am_dzdx
TempData, 84
am_event_model
UserData, 91
am_g
TempData, 83
am_h
UserData, 92
am_id
TempData, 83
am_id_tmp
TempData, 85
am_idlist
UserData, 90
am_interpType
UserData, 90
am_irdiscs
TempData, 86
am_ism
UserData, 90
am_iter
UserData, 91
am_k
UserData, 89
am_lbw
UserData, 91
am_linsol
UserData, 90
am_llhS0
TempData, 83
am_llhdata
ReturnData, 75
am_lmm
UserData, 91
am_max
symbolic_functions.c, 130

am_maxsteps
UserData, 90
am_min
symbolic_functions.c, 129
am_my
ExpData, 71
am_mz
ExpData, 71
am_nan_J
UserData, 92
am_nan_JSparse
UserData, 92
am_nan_dxdotdp
UserData, 92
am_nan_qBdot
UserData, 93
am_nan_xBdot
UserData, 93
am_nan_xdot
UserData, 93
am_ndwdp
UserData, 89
am_ndwdx
UserData, 89
am_ne
UserData, 89
am_nmaxevent
UserData, 89
am_nnz
UserData, 89
am_np
UserData, 88
am_nroots
TempData, 86
am_nt
UserData, 89
am_numrhsevalsSdata
ReturnData, 75
am_numrhsevalsdata
ReturnData, 75
am_numstepsSdata
ReturnData, 74
am_numstepsdata
ReturnData, 74
am_nw
UserData, 89
am_nx
UserData, 88
am_ny
UserData, 88
am_nytrue
UserData, 88
am_nz
UserData, 88
am_nztrue
UserData, 88
am_orderdata
ReturnData, 75
am_ordering
UserData, 91
am_p
UserData, 89
am_pbar
UserData, 89
am_plist
UserData, 88
am_qpositivex
UserData, 88
am_r
TempData, 83
am_rootidx
TempData, 86
am_rootsfound
TempData, 85
am_rootvals
TempData, 86
am_rtol
UserData, 90
am_rval
TempData, 84
am_s2llhdata
ReturnData, 75
am_sdx
TempData, 83
am_sdx_tmp
TempData, 85
am_sensi
UserData, 90
am_sensi_meth
UserData, 90
am_sigma_y
TempData, 84
am_sigma_z
TempData, 84
am_sigmaydata
ReturnData, 74
am_sigmazdata
ReturnData, 74
am_sllhdata
ReturnData, 75
am_ssigmaydata
ReturnData, 74
am_ssigmazdata
ReturnData, 74
am_stldet
UserData, 91
am_sx
TempData, 83
am_sx0data
UserData, 91
am_sx_tmp
TempData, 85
am_sxdata
ReturnData, 74
am_sydata
ReturnData, 74

am_szdata
ReturnData, 74
am_t
TempData, 81
am_ts
UserData, 89
am_tsdata
ReturnData, 73
am_tstart
UserData, 89
am_ubw
UserData, 91
am_w
UserData, 92
am_which
TempData, 86
am_x
TempData, 81
am_x_disc
TempData, 81
am_x_old
TempData, 81
am_x_tmp
TempData, 85
am_xB
TempData, 82
am_xB_old
TempData, 82
am_xB_tmp
TempData, 85
am_xQB
TempData, 82
am_xQB_old
TempData, 82
am_xQB_tmp
TempData, 85
am_xbar
UserData, 90
am_xdata
ReturnData, 74
am_xdot
TempData, 82
am_xdot_disc
TempData, 82
am_xdot_old
TempData, 82
am_xdot_old_disc
TempData, 82
am_xdot_tmp
TempData, 85
am_xdotdata
ReturnData, 73
am_yS0
TempData, 84
am_ydata
ReturnData, 74
am_ysigma
ExpData, 71
am_z2event
UserData, 91
am_zdata
ReturnData, 73
am_zsigma
ExpData, 71
amici.c
AMI_SUCCESS, 99
applyEventBolus, 115
applyEventSensiBolusFSA, 116
fillEventOutput, 110
getDataOutput, 104
getDataSensisASA, 103
getDataSensisFSA, 103
getDiagnosis, 119
getDiagnosisB, 120
getEventObjective, 108
getEventOutput, 109
getEventSensisASA, 106
getEventSensisFSA, 105
getEventSensisFSA_tf, 106
getEventSigma, 107
getTnext, 115
handleDataPoint, 111
handleDataPointB, 112
handleEvent, 113
handleEventB, 114
initField2, 98
initField3, 98
initHeaviside, 116
readOptionData, 98
readOptionScalar, 98
setupAMI, 100
setupAMIB, 102
setupExpData, 100
setupReturnData, 99
setupUserData, 99
updateHeaviside, 118
updateHeavisideB, 118
amidata, 41
condition, 44
ne, 43
nk, 43
nt, 42
ny, 42
nz, 42
Sigma_Y, 43
Sigma_Z, 44
t, 43
Y, 43
Z, 43
amievent, 44
amievent, 45
bolus, 45
hflag, 46
setHflag, 45
trigger, 45
z, 46

amifun, 46
    amifun, 47
    argstr, 50
    cvar, 50
    deps, 50
    fargstr, 50
    funstr, 50
    gccode, 48
    getArgs, 49
    getCVar, 49
    getDeps, 49
    getNVecs, 49
    getSensiFlag, 49
    nvecs, 50
    printLocalVars, 47
    sensiflag, 50
    strsym, 50
    strsym_old, 50
    sym, 49
    writeCcode, 48
    writeCcode_sensi, 48
amilog
    symbolic_functions.c, 128
amimodel, 51
    adjoint, 59
    amimodel, 54
    augmento2, 57
    augmento2vec, 58
    cfun, 64
    checkDeps, 56
    colptrs, 62
    colptrsB, 63
    compileC, 55
    compver, 64
    coptim, 63
    debug, 59
    event, 59
    forward, 59
    fun, 58
    funs, 63
    generateC, 54
    generateM, 55
    getFun, 55
    HTable, 59
    id, 61
    lbw, 62
    loadOldHashes, 57
    maxflag, 65
    minflag, 65
    modelname, 59
    ndwdp, 65
    ndwdx, 65
    nevent, 61
    nk, 61
    nnz, 62
    np, 61
    nw, 65
    nx, 60
    nxtrue, 60
    ny, 60
    nytrue, 60
    nz, 61
    nztrue, 61
    o2flag, 64
    param, 63
    recompile, 64
    rowvals, 62
    rowvalsB, 63
    sparseidx, 62
    sparseidxB, 63
    splineflag, 65
    sym, 58
    t0, 60
    ubw, 62
    updateRHS, 54
    wrap_path, 64
    wtype, 60
    z2event, 64
amioption, 65
    amioption, 67
    atol, 68
    id, 70
    interpType, 69
    ism, 69
    iter, 68
    iterB, 69
    linsol, 68
    lmm, 68
    lmmB, 69
    maxsteps, 68
    nmaxevent, 69
    ordering, 69
    qpositivex, 68
    rtol, 68
    sens_ind, 68
    sensi, 69
    sensi_meth, 69
    ss, 70
    stldet, 68
    sx0, 70
    tstart, 68
    z2event, 70
amiwrap
    amiwrap.m, 95
amiwrap.c, 93
    mexFunction, 94
amiwrap.m, 95
    amiwrap, 95
applyEventBolus
    amici.c, 115
applyEventSensiBolusFSA
    amici.c, 116
argstr
    amifun, 50
atol
    amioption, 68

augmento2
amimodel, 57
augmento2vec
amimodel, 58

bolus
amievent, 45
SBMLode, 79

cfun
amimodel, 64
checkDeps
amimodel, 56
colptrs
amimodel, 62
colptrsB
amimodel, 63
compartment
SBMLode, 78
compileC
amimodel, 55
compver
amimodel, 64
condition
amidata, 44
SBMLode, 79
constant
SBMLode, 78
coptim
amimodel, 63
cvar
amifun, 50

Dam_max
symbolic_functions.c, 130
Dam_min
symbolic_functions.c, 129
debug
amimodel, 59
deps
amifun, 50
deriv
spline.c, 125
Dspline10
symbolic_functions.c, 143
Dspline3
symbolic_functions.c, 138
Dspline4
symbolic_functions.c, 139
Dspline5
symbolic_functions.c, 141
Dspline_pos10
symbolic_functions.c, 144
Dspline_pos3
symbolic_functions.c, 138
Dspline_pos4
symbolic_functions.c, 140
Dspline_pos5
symbolic_functions.c, 142

event
amimodel, 59
ExpData, 70
am_my, 71
am_mz, 71
am_ysigma, 71
am_zsigma, 71

FALSE
symbolic_functions.c, 128
fargstr
amifun, 50
fillEventOutput
amici.c, 110
flux
SBMLode, 79
forward
amimodel, 59
fun
amimodel, 58
funmath
SBMLode, 79
funs
amimodel, 63
funstr
amifun, 50

gccode
amifun, 48
generateC
amimodel, 54
generateM
amimodel, 55
getArgs
amifun, 49
getCVar
amifun, 49
getDataOutput
amici.c, 104
getDataSensisASA
amici.c, 103
getDataSensisFSA
amici.c, 103
getDeps
amifun, 49
getDiagnosis
amici.c, 119
getDiagnosisB
amici.c, 120
getEventObjective
amici.c, 108
getEventOutput
amici.c, 109
getEventSensisASA
amici.c, 106
getEventSensisFSA
amici.c, 105
getEventSensisFSA_tf
amici.c, 106

getEventSigma
amici.c, 107
getFun
amimodel, 55
getNVecs
amifun, 49
getSensiFlag
amifun, 49
getTnext
amici.c, 115
getoptimized
optsym, 72

HTable
amimodel, 59
handleDataPoint
amici.c, 111
handleDataPointB
amici.c, 112
handleEvent
amici.c, 113
handleEventB
amici.c, 114
heaviside
symbolic_functions.c, 128
hflag
amievent, 46

id
amimodel, 61
amioption, 70
initField2
amici.c, 98
initField3
amici.c, 98
initHeaviside
amici.c, 116
initState
SBMLode, 79
interpType
amioption, 69
ism
amioption, 69
iter
amioption, 68
iterB
amioption, 69

knom
SBMLode, 80

lbw
amimodel, 62
linsol
amioption, 68
lmm
amioption, 68
lmmB
amioption, 69
loadOldHashes
amimodel, 57

maxflag
amimodel, 65
maxsteps
amioption, 68
mexFunction
amiwrap.c, 94
minflag
amimodel, 65
modelTest, 71
modelname
amimodel, 59

ndwdp
amimodel, 65
ndwdx
amimodel, 65
ne
amidata, 43
nevent
amimodel, 61
nk
amidata, 43
amimodel, 61
nmaxevent
amioption, 69
nnz
amimodel, 62
np
amimodel, 61
nt
amidata, 42
nvecs
amifun, 50
nw
amimodel, 65
nx
amimodel, 60
nxtrue
amimodel, 60
ny
amidata, 42
amimodel, 60
nytrue
amimodel, 60
nz
amidata, 42
amimodel, 61
nztrue
amimodel, 61

o2flag
amimodel, 64
observable
SBMLode, 78
observable_name
SBMLode, 78

optsym, 71
getoptimized, 72
ordering
amioption, 69

param
amimodel, 63
SBMLode, 78
parameter
SBMLode, 78
pnom
SBMLode, 80
printLocalVars
amifun, 47

qpositivex
amioption, 68

reaction
SBMLode, 78
readOptionData
amici.c, 98
readOptionScalar
amici.c, 98
recompile
amimodel, 64
ReturnData, 72
am_Jdata, 73
am_chi2data, 75
am_dxdotdpdata, 73
am_dydpdata, 73
am_dydxdata, 73
am_llhdata, 75
am_numrhsevalsSdata, 75
am_numrhsevalsdata, 75
am_numstepsSdata, 74
am_numstepsdata, 74
am_orderdata, 75
am_s2llhdata, 75
am_sigmaydata, 74
am_sigmazdata, 74
am_sllhdata, 75
am_ssigmaydata, 74
am_ssigmazdata, 74
am_sxdata, 74
am_sydata, 74
am_szdata, 74
am_tsdata, 73
am_xdata, 74
am_xdotdata, 73
am_ydata, 74
am_zdata, 73
rowvals
amimodel, 62
rowvalsB
amimodel, 63
rtol
amioption, 68

SBML2AMICI
SBML2AMICI.m, 96
SBML2AMICI.m, 96
SBML2AMICI, 96
SBMLode, 75
bolus, 79
compartment, 78
condition, 79
constant, 78
flux, 79
funmath, 79
initState, 79
knom, 80
observable, 78
observable_name, 78
param, 78
parameter, 78
pnom, 80
reaction, 78
SBMLode, 77
state, 78
stochiometry, 79
time_symbol, 79
trigger, 79
volume, 78
writeAMICI, 77
xdot, 79
sens_ind
amioption, 68
sensi
amioption, 69
sensi_meth
amioption, 69
sensiflag
amifun, 50
setHflag
amievent, 45
setupAMI
amici.c, 100
setupAMIB
amici.c, 102
setupExpData
amici.c, 100
setupReturnData
amici.c, 99
setupUserData
amici.c, 99
seval
spline.c, 123
Sigma_Y
amidata, 43
Sigma_Z
amidata, 44
sign
symbolic_functions.c, 129
sinteg
spline.c, 126
sparseidx

amimodel, 62
sparseidxB
amimodel, 63
spline
spline.c, 121
spline.c
deriv, 125
seval, 123
sinteg, 126
spline, 121
spline10
symbolic_functions.c, 135
spline3
symbolic_functions.c, 130
spline4
symbolic_functions.c, 132
spline5
symbolic_functions.c, 134
spline_pos10
symbolic_functions.c, 136
spline_pos3
symbolic_functions.c, 131
spline_pos4
symbolic_functions.c, 133
spline_pos5
symbolic_functions.c, 134
splineflag
amimodel, 65
src/amici.c, 96
src/spline.c, 120
src/symbolic_functions.c, 126
ss
amioption, 70
state
SBMLode, 78
stldet
amioption, 68
stochiometry
SBMLode, 79
strsym
amifun, 50
strsym_old
amifun, 50
sx0
amioption, 70
sym
amifun, 49
amimodel, 58
symbolic/am_and.m, 145
symbolic/am_ge.m, 145
symbolic/am_gt.m, 146
symbolic/am_if.m, 146
symbolic/am_le.m, 146
symbolic/am_lt.m, 146
symbolic/am_max.m, 146
symbolic/am_min.m, 147
symbolic/am_or.m, 147
symbolic/am_stepfun.m, 147
symbolic_functions.c
am_max, 130
am_min, 129
amilog, 128
Dam_max, 130
Dam_min, 129
Dspline10, 143
Dspline3, 138
Dspline4, 139
Dspline5, 141
Dspline_pos10, 144
Dspline_pos3, 138
Dspline_pos4, 140
Dspline_pos5, 142
FALSE, 128
heaviside, 128
sign, 129
spline10, 135
spline3, 130
spline4, 132
spline5, 134
spline_pos10, 136
spline_pos3, 131
spline_pos4, 133
spline_pos5, 134
TRUE, 128

t
amidata, 43
t0
amimodel, 60
TRUE
symbolic_functions.c, 128
TempData, 80
am_Jtmp, 83
am_deltaqB, 86
am_deltasx, 86
am_deltax, 86
am_deltaxB, 86
am_dgdp, 83
am_dgdx, 83
am_discs, 86
am_drdp, 83
am_drdx, 83
am_drvaldp, 84
am_drvaldx, 84
am_dsigma_ydp, 84
am_dsigma_zdp, 85
am_dx, 82
am_dx_old, 82
am_dx_tmp, 85
am_dxB, 82
am_dxB_tmp, 85
am_dydp, 84
am_dydx, 84
am_dzdp, 84
am_dzdx, 84
am_g, 83
am_id, 83

am_id_tmp, 85
am_irdiscs, 86
am_llhS0, 83
am_nroots, 86
am_r, 83
am_rootidx, 86
am_rootsfound, 85
am_rootvals, 86
am_rval, 84
am_sdx, 83
am_sdx_tmp, 85
am_sigma_y, 84
am_sigma_z, 84
am_sx, 83
am_sx_tmp, 85
am_t, 81
am_which, 86
am_x, 81
am_x_disc, 81
am_x_old, 81
am_x_tmp, 85
am_xB, 82
am_xB_old, 82
am_xB_tmp, 85
am_xQB, 82
am_xQB_old, 82
am_xQB_tmp, 85
am_xdot, 82
am_xdot_disc, 82
am_xdot_old, 82
am_xdot_old_disc, 82
am_xdot_tmp, 85
am_yS0, 84

time_symbol
SBMLode, 79

trigger
amievent, 45
SBMLode, 79

tstart
amioption, 68

ubw
amimodel, 62

updateHeaviside
amici.c, 118

updateHeavisideB
amici.c, 118

updateRHS
amimodel, 54

UserData, 87
am_J, 92
am_M, 92
am_atol, 90
am_bsx0, 91
am_dfdx, 92
am_dwdp, 92
am_dwdx, 92
am_dxdotdp, 92
am_event_model, 91
am_h, 92
am_idlist, 90
am_interpType, 90
am_ism, 90
am_iter, 91
am_k, 89
am_lbw, 91
am_linsol, 90
am_lmm, 91
am_maxsteps, 90
am_nan_J, 92
am_nan_JSparse, 92
am_nan_dxdotdp, 92
am_nan_qBdot, 93
am_nan_xBdot, 93
am_nan_xdot, 93
am_ndwdp, 89
am_ndwdx, 89
am_ne, 89
am_nmaxevent, 89
am_nnz, 89
am_np, 88
am_nt, 89
am_nw, 89
am_nx, 88
am_ny, 88
am_nytrue, 88
am_nz, 88
am_nztrue, 88
am_ordering, 91
am_p, 89
am_pbar, 89
am_plist, 88
am_qpositivex, 88
am_rtol, 90
am_sensi, 90
am_sensi_meth, 90
am_stldet, 91
am_sx0data, 91
am_ts, 89
am_tstart, 89
am_ubw, 91
am_w, 92
am_xbar, 90
am_z2event, 91

volume
SBMLode, 78

wrap_path
amimodel, 64

writeAMICI
SBMLode, 77

writeCcode
amifun, 48

writeCcode_sensi
amifun, 48

wtype
amimodel, 60

xdot
SBMLode, 79

Y
amidata, 43

Z
amidata, 43
z
amievent, 46
z2event
amimodel, 64
amioption, 70
