# Supplementary material for: Scalable Parameter Estimation for Genome-Scale Biochemical Reaction Networks
Source: PLoS Comput Biol. 2017 Jan 23;13(1):e1005331. doi: 10.1371/journal.pcbi.1005331 (PMC5256869; doi:10.1371/journal.pcbi.1005331)
Supplement: S1 Code — This zip-file contains the MATLAB code for the simulation and application examples presented in the paper. We provide implementations of all models, parameter estimation to allow everybody to reproduce the results. (ZIP) [file pcbi.1005331.s002.zip › code/AMICI/examples/example_dirac_adjoint/html/example_dirac_adjoint.html]

example\_dirac\_adjoint 

```
function example_dirac_adjoint()
```

COMPILATION

```
    [exdir,~,~]=fileparts(which('example_dirac_adjoint.m'));
    % compile the model
    amiwrap('model_dirac_adjoint','model_dirac_adjoint_syms',exdir)
```

```
Generating model struct ...
Parsing model struct ...
Generating C code ...
headers | wrapfunctions | Compiling mex file ...
amici | Building with 'Xcode with Clang'.
MEX completed successfully.
Building with 'Xcode with Clang'.
MEX completed successfully.
```

SIMULATION

```
    % time vector
    tout = linspace(0,4,9);
    tfine = linspace(0,4,10001);
    p = [1;0.4;2;3];
    k = [];

    D.Y = [  0.00714742903826096
        -0.00204966058299775
        0.382159034587845
        0.33298932672138
        0.226111476113441
        0.147028440865854
        0.0882468698791813
        0.0375887796628869
        0.0373422340295005];

    D.Sigma_Y = 0.01*ones(size(D.Y));


    options.sensi = 1;
    options.sensi_meth = 'adjoint';
    options.maxsteps = 1e5;
    sol = simulate_model_dirac_adjoint(tout,log10(p),k,D,options);
    options.sensi = 0;
    solfine = simulate_model_dirac_adjoint(tfine,log10(p),k,[],options);
    figure
    errorbar(tout,D.Y,D.Sigma_Y)
    hold on
    plot(tfine,solfine.y)
    legend('data','simulation')
    xlabel('time t')
    ylabel('observable')
    title(['log-likelihood: ' num2str(sol.llh) ])
```

FD

```
    eps = 1e-4;
    xi = log10(p);
    grad_fd_f = NaN(4,1);
    grad_fd_b = NaN(4,1);
    for ip = 1:4;
        options.sensi = 0;
        xip = xi;
        xip(ip) = xip(ip) + eps;
        solpf = simulate_model_dirac_adjoint(tout,xip,k,D,options);
        grad_fd_f(ip,1) = (solpf.llh-sol.llh)/eps;
        xip = xi;
        xip(ip) = xip(ip) - eps;
        solpb = simulate_model_dirac_adjoint(tout,xip,k,D,options);
        grad_fd_b(ip,1) = -(solpb.llh-sol.llh)/eps;
    end

    figure
    plot(abs(grad_fd_f),abs(sol.sllh),'o')
    hold on
    plot(abs(grad_fd_b),abs(sol.sllh),'o')
    set(gca,'XScale','log')
    set(gca,'YScale','log')
    hold on
    axis square
    plot([1e2,1e4],[1e2,1e4],'k:')
    xlim([1e2,1e4])
    ylim([1e2,1e4])
    legend('forward FD','backward FD','Location','SouthEast')
    xlabel('adjoint sensitivity absolute value of gradient element')
    ylabel('computed absolute value of gradient element')
    set(gcf,'Position',[100 300 1200 500])

    drawnow
```

```
end
```

Published with MATLAB® R2016a
