# Supplementary material for: Scalable Parameter Estimation for Genome-Scale Biochemical Reaction Networks
Source: PLoS Comput Biol. 2017 Jan 23;13(1):e1005331. doi: 10.1371/journal.pcbi.1005331 (PMC5256869; doi:10.1371/journal.pcbi.1005331)
Supplement: S1 Code — This zip-file contains the MATLAB code for the simulation and application examples presented in the paper. We provide implementations of all models, parameter estimation to allow everybody to reproduce the results. (ZIP) [file pcbi.1005331.s002.zip › code/AMICI/examples/example_dirac_secondorder/html/model_dirac_secondorder_syms.html]

model\_dirac\_secondorder\_syms 

```
function [model] = model_dirac_secondorder_syms()
```

STATES

```
% create state syms
syms x1 x2

% create state vector
model.sym.x = [ x1 x2 ];
```

PARAMETERS ( for these sensitivities will be computed )

```
% create parameter syms
syms p1 p2 p3 p4

% create parameter vector
model.sym.p = [p1,p2,p3,p4];

% set the parametrisation of the problem options are 'log', 'log10' and
% 'lin' (default).
model.param = 'log10';
```

SYSTEM EQUATIONS

```
% create symbolic variable for time
syms t

model.sym.xdot = sym(zeros(size(model.sym.x)));

% piecewise defined function
model.sym.xdot(1) = -p1*x1 + dirac(t-p2);
% inhomogeneous
model.sym.xdot(2) = p3*x1 - p4*x2 ;
```

INITIAL CONDITIONS

```
model.sym.x0 = sym(zeros(size(model.sym.x)));

model.sym.x0(1) = 0;
model.sym.x0(2) = 0;
```

OBSERVALES

```
model.sym.y = sym(zeros(1,1));

model.sym.y(1) = x2;
```

```
end
```

```
ans = 
      sym: [1x1 struct]
    param: 'log10'
```

Published with MATLAB® R2016a
