# Supplementary material for: Scalable Parameter Estimation for Genome-Scale Biochemical Reaction Networks
Source: PLoS Comput Biol. 2017 Jan 23;13(1):e1005331. doi: 10.1371/journal.pcbi.1005331 (PMC5256869; doi:10.1371/journal.pcbi.1005331)
Supplement: S1 Code — This zip-file contains the MATLAB code for the simulation and application examples presented in the paper. We provide implementations of all models, parameter estimation to allow everybody to reproduce the results. (ZIP) [file pcbi.1005331.s002.zip › code/AMICI/examples/example_dirac_secondorder_vectmult/html/example_dirac_secondorder_vectmult.html]

example\_dirac\_secondorder\_vectmult 

```
function example_dirac_secondorder_vectmult()
```

COMPILATION

```
    [exdir,~,~]=fileparts(which('example_dirac_secondorder_vectmult.m'));
    % compile the model
    amiwrap('model_dirac_secondorder_vectmult','model_dirac_secondorder_vectmult_syms',exdir,2)
```

```
Generating model struct ...
x | k | p | deltax | xdot | deltaxdot | ddeltaxdx | ddeltaxdp | ddeltaxdt | root | drootdx | sx | drootdp | drootdt | dtaudp | sroot | stau | deltasx | sigma_y | dsigma_ydp | y | dydp | sigma_z | dsigma_zdp | z | dzdp | Parsing model struct ...
z | Generating C code ...
deltasx | deltax | dsigma_ydp | dsigma_zdp | dydp | dzdp | root | sigma_y | sigma_z | stau | xdot | y | z | headers | wrapfunctions | headers | wrapfunctions | Compiling mex file ...
amici | Building with 'Xcode with Clang'.
MEX completed successfully.
Building with 'Xcode with Clang'.
MEX completed successfully.
amici | Building with 'Xcode with Clang'.
MEX completed successfully.
Building with 'Xcode with Clang'.
MEX completed successfully.
```

SIMULATION

```
    % time vector
    t = linspace(0,3,1001);
    p = [1;0.5;2;3];
    k = [];
    v = [0.7;0.3;1.4;0.1];

    options = amioption('sensi',0,...
        'maxsteps',1e4);

    % load mex into memory
    [msg] = which('model_dirac_secondorder_vectmult'); % fix for inaccessability problems
    options.sensi = 2;
    sol = simulate_model_dirac_secondorder_vectmult(t,log10(p),k,[],options,v);
```

FORWARD SENSITIVITY ANALYSIS

```
    options.sensi = 2;

    sol = simulate_model_dirac_secondorder_vectmult(t,log10(p),k,[],options,v);
```

FINITE DIFFERENCES

```
    options.sensi = 1;

    eps = 1e-4;
    xi = log10(p);
    for ip = 1:4;
        xip = xi;
        xip(ip) = xip(ip) + eps;
        solp = simulate_model_dirac_secondorder_vectmult(t,xip,k,[],options);
        s2x_fd(:,:,ip) = sum(bsxfun(@times,(solp.sx - sol.sx)/eps,permute(v,[3,2,1])),3);
        s2y_fd(:,:,ip) = sum(bsxfun(@times,(solp.sy - sol.sy)/eps,permute(v,[3,2,1])),3);
    end
```

PLOTTING

```
    figure
    c_x = get(gca,'ColorOrder');
    for ip = 1:4
        subplot(4,2,ip*2-1)
        hold on
        for ix = 1:size(sol.x,2)
            plot(t,sol.s2x(:,ix,ip),'.-','Color',c_x(ix,:))
            plot(t,s2x_fd(:,ix,ip),'--','Color',c_x(ix,:))
        end
        ylim([-10,10])
        legend('x1','x1_{fd}','x2','x2_{fd}','Location','NorthEastOutside')
        legend boxoff
        title(['state sensitivity for p' num2str(ip)])
        xlabel('time t')
        ylabel('x')
        box on

        subplot(4,2,ip*2)
        plot(t,abs(sol.s2x(:,:,ip)-s2x_fd(:,:,ip)),'r--')
        legend('error x1','error x2','Location','NorthEastOutside')
        legend boxoff
        title(['state sensitivity for p' num2str(ip)])
        xlabel('time t')
        ylabel('error')
        ylim([1e-12,1e0])
        set(gca,'YScale','log')
        box on
    end
    set(gcf,'Position',[100 300 1200 500])

    figure
    for ip = 1:4
        subplot(4,2,ip*2-1)
        hold on
        for iy = 1:size(sol.y,2)
            plot(t,sol.s2y(:,iy,ip),'.-','Color',c_x(iy,:))
            plot(t,s2y_fd(:,iy,ip),'--','Color',c_x(iy,:))
        end
        ylim([-10,10])
        legend('y1','y1_{fd}','Location','NorthEastOutside')
        legend boxoff
        title(['observable sensitivity for p' num2str(ip)])
        xlabel('time t')
        ylabel('y')
        box on

        subplot(4,2,ip*2)
        plot(t,abs(sol.s2y(:,:,ip)-s2y_fd(:,:,ip)),'r--')
        legend('error y1','Location','NorthEastOutside')
        legend boxoff
        title(['observable sensitivity for p' num2str(ip)])
        xlabel('time t')
        ylabel('error')
        ylim([1e-12,1e0])
        set(gca,'YScale','log')
        box on
    end
    set(gcf,'Position',[100 300 1200 500])

    drawnow
```

 

```
end
```

Published with MATLAB® R2016a
