# Supplementary material for: Scalable Parameter Estimation for Genome-Scale Biochemical Reaction Networks
Source: PLoS Comput Biol. 2017 Jan 23;13(1):e1005331. doi: 10.1371/journal.pcbi.1005331 (PMC5256869; doi:10.1371/journal.pcbi.1005331)
Supplement: S1 Code — This zip-file contains the MATLAB code for the simulation and application examples presented in the paper. We provide implementations of all models, parameter estimation to allow everybody to reproduce the results. (ZIP) [file pcbi.1005331.s002.zip › code/AMICI/examples/example_events/html/example_events.html]

example\_events 

```
function example_events()
```

COMPILATION

```
[exdir,~,~]=fileparts(which('example_events.m'));
% compile the model
amiwrap('model_events','model_events_syms',exdir)
```

```
Generating model struct ...
Parsing model struct ...
Generating C code ...
headers | wrapfunctions | Compiling mex file ...
amici | Building with 'Xcode with Clang'.
MEX completed successfully.
Building with 'Xcode with Clang'.
MEX completed successfully.
```

SIMULATION

```
% time vector
t = linspace(0,10,20);
p = [0.5;2;0.5;0.5];
k = [4,8,10,4];

options = amioption('sensi',0,...
    'maxsteps',1e4,...
    'nmaxevent', 2);
D = amidata(length(t),1,2,2,4);
% load mex into memory
[~] = which('simulate_model_events'); % fix for inaccessability problems
sol = simulate_model_events(t,log10(p),k,D,options);

tic
sol = simulate_model_events(t,log10(p),k,D,options);
disp(['Time elapsed with cvodes: ' num2str(toc) ])
```

```
Time elapsed with cvodes: 0.0033863
```

ODE15S

```
ode_system = @(t,x,p,k) [-p(1)*heaviside(t-p(4))*x(1);
    +p(2)*x(1)*exp(-0.1*t)-p(3)*x(2);
    -1.5*x(3)];
% event_fn = @(t,x) [x(3) - x(2);
%     x(3) - x(1)];
% 'Events',event_fn
options_ode15s = odeset('RelTol',options.rtol,'AbsTol',options.atol,'MaxStep',options.maxsteps);

tic
[~, X_ode15s] = ode15s(@(t,x) ode_system(t,x,p,k),t,k(1:3),options_ode15s);
disp(['Time elapsed with ode15s: ' num2str(toc) ])
```

```
Time elapsed with ode15s: 0.13084
```

PLOTTING

```
figure
c_x = get(gca,'ColorOrder');
subplot(2,2,1)
for ix = 1:size(sol.x,2)
    plot(t,sol.x(:,ix),'.-','Color',c_x(ix,:))
    hold on
    plot(t,X_ode15s(:,ix),'d','Color',c_x(ix,:))
end
stem(sol.z(:,1),sol.z(:,1)*0+10,'r')
stem(sol.z(:,2),sol.z(:,2)*0+10,'k')
legend('x1','x1_{ode15s}','x2','x2_{ode15s}','x3','x3_{ode15s}','x3==x2','x3==x1','Location','NorthEastOutside')
legend boxoff
xlabel('time t')
ylabel('x')
box on
subplot(2,2,2)
plot(t,abs(sol.x-X_ode15s),'--')
set(gca,'YScale','log')
legend('error x1','error x2','error x3','Location','NorthEastOutside')
legend boxoff
ylabel('x')

subplot(2,2,3)
plot(t,sol.y,'.-','Color',c_x(1,:))
hold on
plot(t,p(4)*sum(X_ode15s,2),'d','Color',c_x(1,:))
legend('y1','y1_{ode15s}','Location','NorthEastOutside')
legend boxoff
xlabel('time t')
ylabel('y')
box on

subplot(2,2,4)
plot(t,abs(sol.y-p(4)*sum(X_ode15s,2)),'--')
set(gca,'YScale','log')
legend('error y1','Location','NorthEastOutside')
legend boxoff
xlabel('time t')
ylabel('y')
box on

set(gcf,'Position',[100 300 1200 500])
```

FORWARD SENSITIVITY ANALYSIS

```
options.sensi = 1;

sol = simulate_model_events(t,log10(p),k,D,options);
```

FINITE DIFFERENCES

```
eps = 1e-4;
xi = log10(p);
for ip = 1:4;
    xip = xi;
    xip(ip) = xip(ip) + eps;
    solp = simulate_model_events(t,xip,k,D,options);
    sx_fd(:,:,ip) = (solp.x - sol.x)/eps;
    sy_fd(:,:,ip) = (solp.y - sol.y)/eps;
    sz_fd(:,:,ip) = (solp.z - sol.z)/eps;
end
```

PLOTTING

```
figure
for ip = 1:4
    subplot(4,2,ip*2-1)
    hold on
    for ix = 1:size(sol.x,2)
        plot(t,sol.sx(:,ix,ip),'.-','Color',c_x(ix,:))
        plot(t,sx_fd(:,ix,ip),'d','Color',c_x(ix,:))
    end
    legend('sx1','sx1_{fd}','sx2','sx2_{fd}','sx3','sx3_{fd}','Location','NorthEastOutside')
    legend boxoff
    title(['state sensitivity for p' num2str(ip)])
    xlabel('time t')
    ylabel('sx')
    box on

    subplot(4,2,ip*2)
    plot(t,abs(sol.sx(:,:,ip)-sx_fd(:,:,ip)),'--')
    legend('error sx1','error sx2','error sx3','Location','NorthEastOutside')
    legend boxoff
    title(['state sensitivity for p' num2str(ip)])
    xlabel('time t')
    ylabel('error')
    set(gca,'YScale','log')
    box on
end
set(gcf,'Position',[100 300 1200 500])

figure
for ip = 1:4
    subplot(4,2,ip*2-1)
    hold on
    for iy = 1:size(sol.y,2)
        plot(t,sol.sy(:,iy,ip),'.-','Color',c_x(iy,:))
        plot(t,sy_fd(:,iy,ip),'d','Color',c_x(iy,:))
    end
    legend('sy1','sy1_fd','Location','NorthEastOutside')
    legend boxoff
    title(['observable sensitivity for p' num2str(ip)])
    xlabel('time t')
    ylabel('sy')
    box on

    subplot(4,2,ip*2)
    plot(t,abs(sol.sy(:,:,ip)-sy_fd(:,:,ip)),'--')
    legend('error sy1','Location','NorthEastOutside')
    legend boxoff
    title(['error observable sensitivity for p' num2str(ip)])
    xlabel('time t')
    ylabel('error')
    set(gca,'YScale','log')
    box on
end
set(gcf,'Position',[100 300 1200 500])

figure
for ip = 1:4
subplot(4,2,2*ip-1)
bar(1:options.nmaxevent,sol.sz(1:options.nmaxevent,:,ip),0.8)
hold on
bar(1:options.nmaxevent,sz_fd(1:options.nmaxevent,:,ip),0.4)
legend('x3==x2','x3==x1','x3==x2 fd','x3==x1 fd','Location','NorthEastOutside')
legend boxoff
title(['event sensitivity for p' num2str(ip)])
xlabel('event #')
ylabel('sz')
box on

subplot(4,2,2*ip)
bar(1:options.nmaxevent,sol.sz(1:options.nmaxevent,:,ip)-sz_fd(1:options.nmaxevent,:,ip),0.8)
legend('error x3==x2','error x3==x1','Location','NorthEastOutside')
legend boxoff
title(['error event sensitivity for p' num2str(ip)])
xlabel('event #')
ylabel('sz')
box on
end
set(gcf,'Position',[100 300 1200 500])

drawnow
```

  

```
end
```

Published with MATLAB® R2016a
