# Supplementary material for: Scalable Parameter Estimation for Genome-Scale Biochemical Reaction Networks
Source: PLoS Comput Biol. 2017 Jan 23;13(1):e1005331. doi: 10.1371/journal.pcbi.1005331 (PMC5256869; doi:10.1371/journal.pcbi.1005331)
Supplement: S1 Code — This zip-file contains the MATLAB code for the simulation and application examples presented in the paper. We provide implementations of all models, parameter estimation to allow everybody to reproduce the results. (ZIP) [file pcbi.1005331.s002.zip › code/AMICI/examples/example_jakstat_adjoint/html/example_jakstat_adjoint.html]

example\_jakstat\_adjoint 

```
function example_jakstat_adjoint()

    % compile the model
    [exdir,~,~]=fileparts(which('example_jakstat_adjoint.m'));
    amiwrap('model_jakstat','model_jakstat_adjoint_syms',exdir)

    num = xlsread(fullfile(exdir,'pnas_data_original.xls'));

    D.t = num(:,1);
    D.condition= [1.4,0.45];
    D.Y = num(:,[2,4,6]);
    D.Sigma_Y = NaN(size(D.Y));
    D = amidata(D);

    xi =  [0.60
        3
        -0.95
        -0.0075
        0
        -2.8
        -0.26
        -0.075
        -0.41
        -5
        -0.74
        -0.64
        -0.11
        0.027
        -0.5
        0
        -0.5];

    options.sensi = 0;
    sol = simulate_model_jakstat([],xi,[],D,options);

    figure
    for iy = 1:3
        subplot(2,2,iy)
        plot(D.t,D.Y(:,iy),'rx')
        hold on
        plot(sol.t,sol.y(:,iy),'.-')
        xlim([0,60])
        xlabel('t')
        switch(iy)
            case 1
                ylabel('pStat')
            case 2
                ylabel('tStat')
            case 3
                ylabel('pEpoR')
        end
        ylim([0,1.2])
    end
    set(gcf,'Position',[100 300 1200 500])

    % generate new
    xi_rand = xi + 0.1;
    options.sensi = 1;
    options.sensi_meth = 'adjoint';
    sol = simulate_model_jakstat([],xi_rand,[],D,options);

    options.sensi = 0;
    eps = 1e-4;
    fd_grad = NaN(length(xi),1);
    for ip = 1:length(xi)
        xip = xi_rand;
        xip(ip) = xip(ip) + eps;
        psol = simulate_model_jakstat([],xip,[],D,options);
        fd_grad(ip) = (psol.llh-sol.llh)/eps;
    end

    figure
    scatter(abs(sol.sllh),abs(fd_grad))
    set(gca,'XScale','log')
    set(gca,'YScale','log')
    xlim([1e-2,1e2])
    ylim([1e-2,1e2])
    box on
    hold on
    axis square
    plot([1e-2,1e2],[1e-2,1e2],'k:')
    xlabel('adjoint sensitivity absolute value of gradient element')
    ylabel('finite difference absolute value of gradient element')
    set(gcf,'Position',[100 300 1200 500])


    drawnow

end
```

```
Generating model struct ...
Parsing model struct ...
Generating C code ...
headers | wrapfunctions | Compiling mex file ...
amici | Building with 'Xcode with Clang'.
MEX completed successfully.
Building with 'Xcode with Clang'.
MEX completed successfully.
```

 

Published with MATLAB® R2016a
