# Supplementary material for: Scalable Parameter Estimation for Genome-Scale Biochemical Reaction Networks
Source: PLoS Comput Biol. 2017 Jan 23;13(1):e1005331. doi: 10.1371/journal.pcbi.1005331 (PMC5256869; doi:10.1371/journal.pcbi.1005331)
Supplement: S1 Code — This zip-file contains the MATLAB code for the simulation and application examples presented in the paper. We provide implementations of all models, parameter estimation to allow everybody to reproduce the results. (ZIP) [file pcbi.1005331.s002.zip › code/AMICI/examples/example_jakstat_adjoint/html/model_jakstat_adjoint_syms.html]

model\_jakstat\_adjoint\_syms 

```
function [model] = model_jakstat_syms()
```

STATES

```
    syms STAT pSTAT pSTAT_pSTAT npSTAT_npSTAT nSTAT1 nSTAT2 nSTAT3 nSTAT4 nSTAT5

    model.sym.x = [
        STAT, pSTAT, pSTAT_pSTAT, npSTAT_npSTAT, nSTAT1, nSTAT2, nSTAT3, nSTAT4, nSTAT5 ...
        ];
```

PARAMETERS

```
    syms p1 p2 p3 p4 init_STAT Omega_cyt Omega_nuc sp1 sp2 sp3 sp4 sp5 offset_tSTAT offset_pSTAT scale_tSTAT scale_pSTAT sigma_pSTAT sigma_tSTAT sigma_pEpoR

    model.sym.p = [p1,p2,p3,p4,init_STAT,sp1,sp2,sp3,sp4,sp5,offset_tSTAT,offset_pSTAT,scale_tSTAT,scale_pSTAT,sigma_pSTAT,sigma_tSTAT,sigma_pEpoR];

    model.param = 'log10';

    model.sym.k = [Omega_cyt,Omega_nuc];
```

INPUT

```
    syms t
    u(1) = spline_pos5(t, 0.0, sp1, 5.0, sp2, 10.0, sp3, 20.0, sp4, 60.0, sp5, 0, 0.0);
```

```
Warning: Support of strings that are not valid variable names or define a number
will be removed in a future release. To create symbolic expressions, first
create symbolic variables and then use operations on them.
```

SYSTEM EQUATIONS

```
    model.sym.xdot = sym(zeros(size(model.sym.x)));

    model.sym.xdot(1) = (Omega_nuc*p4*nSTAT5 - Omega_cyt*STAT*p1*u(1))/Omega_cyt;
    model.sym.xdot(2) = STAT*p1*u(1) - 2*p2*pSTAT^2;
    model.sym.xdot(3) = p2*pSTAT^2 - p3*pSTAT_pSTAT;
    model.sym.xdot(4) = -(Omega_nuc*p4*npSTAT_npSTAT - Omega_cyt*p3*pSTAT_pSTAT)/Omega_nuc;
    model.sym.xdot(5) = -p4*(nSTAT1 - 2*npSTAT_npSTAT);
    model.sym.xdot(6) = p4*(nSTAT1 - nSTAT2);
    model.sym.xdot(7) = p4*(nSTAT2 - nSTAT3);
    model.sym.xdot(8) = p4*(nSTAT3 - nSTAT4);
    model.sym.xdot(9) = p4*(nSTAT4 - nSTAT5);
```

INITIAL CONDITIONS

```
    model.sym.x0 = sym(zeros(size(model.sym.x)));

    model.sym.x0(1) = init_STAT;
```

OBSERVABLES

```
    model.sym.y = sym(zeros(3,1));

    model.sym.y(1) = offset_pSTAT + scale_pSTAT/init_STAT*(pSTAT + 2*pSTAT_pSTAT);
    model.sym.y(2) = offset_tSTAT + scale_tSTAT/init_STAT*(STAT + pSTAT + 2*(pSTAT_pSTAT));
    model.sym.y(3) = u(1);
```

SIGMA

```
    model.sym.sigma_y = sym(size(model.sym.y));

    model.sym.sigma_y(1) = sigma_pSTAT;
    model.sym.sigma_y(2) = sigma_tSTAT;
    model.sym.sigma_y(3) = sigma_pEpoR;
```

```
end
```

```
ans = 
      sym: [1x1 struct]
    param: 'log10'
```

Published with MATLAB® R2016a
