# Supplementary material for: Scalable Parameter Estimation for Genome-Scale Biochemical Reaction Networks
Source: PLoS Comput Biol. 2017 Jan 23;13(1):e1005331. doi: 10.1371/journal.pcbi.1005331 (PMC5256869; doi:10.1371/journal.pcbi.1005331)
Supplement: S1 Code — This zip-file contains the MATLAB code for the simulation and application examples presented in the paper. We provide implementations of all models, parameter estimation to allow everybody to reproduce the results. (ZIP) [file pcbi.1005331.s002.zip › code/AMICI/examples/example_steadystate/html/example_steadystate.html]

example\_steadystate 

```
function example_steadystate
```

COMPILATION

```
    [exdir,~,~]=fileparts(which('example_steadystate.m'));
    % compile the model
    amiwrap('model_steadystate','model_steadystate_syms',exdir)
```

```
Generating model struct ...
Parsing model struct ...
Generating C code ...
headers | wrapfunctions | Compiling mex file ...
amici | Building with 'Xcode with Clang'.
MEX completed successfully.
Building with 'Xcode with Clang'.
MEX completed successfully.
```

SIMULATION

```
    % time vector
    t = linspace(0,300,20);
    p = [1;0.5;0.4;2;0.1];
    k = [0.1,0.4,0.7,1];

    options = amioption('sensi',0,...
        'maxsteps',1e4);
    % load mex into memory
    sol = simulate_model_steadystate(t,log10(p),k,[],options);

    tic
    sol = simulate_model_steadystate(t,log10(p),k,[],options);
    disp(['Time elapsed with cvodes: ' num2str(toc) ])
```

```
Time elapsed with cvodes: 0.0040652
```

ODE15S

```
    ode_system = @(t,x,p,k) [-2*p(1)*x(1)^2 - p(2)*x(1)*x(2) + 2*p(3)*x(2) + p(4)*x(3) + p(5);
        + p(1)*x(1)^2 - p(2)*x(1)*x(2) - p(3)*x(2) + p(4)*x(3);
        + p(2)*x(1)*x(2) - p(4)*x(3) - k(4)*x(3)];
    options_ode15s = odeset('RelTol',options.rtol,'AbsTol',options.atol,'MaxStep',options.maxsteps);

    tic
    [~, X_ode15s] = ode15s(@(t,x) ode_system(t,x,p,k),t,k(1:3),options_ode15s);
    disp(['Time elapsed with ode15s: ' num2str(toc) ])
```

```
Time elapsed with ode15s: 0.10806
```

PLOTTING

```
    figure
    c_x = get(gca,'ColorOrder');
    subplot(2,2,1)
    for ix = 1:size(sol.x,2)
        plot(t,sol.x(:,ix),'.-','Color',c_x(ix,:))
        hold on
        plot(t,X_ode15s(:,ix),'d','Color',c_x(ix,:))
    end
    legend('x1','x1_{ode15s}','x2','x2_{ode15s}','x3','x3_{ode15s}','Location','NorthEastOutside')
    legend boxoff
    xlabel('time t')
    ylabel('x')
    box on
    subplot(2,2,2)
    plot(t,abs(sol.x-X_ode15s),'--')
    set(gca,'YScale','log')
    legend('error x1','error x2','error x3','Location','NorthEastOutside')
    legend boxoff
    set(gcf,'Position',[100 300 1200 500])
```

FORWARD SENSITIVITY ANALYSIS

```
    options.sensi = 1;
    options.sens_ind = [3,1,2,4];

    sol = simulate_model_steadystate(t,log10(p),k,[],options);
```

FINITE DIFFERENCES

```
    eps = 1e-3;

    xi = log10(p);
    for ip = 1:4;
        xip = xi;
        xip(ip) = xip(ip) + eps;
        solp = simulate_model_steadystate(t,xip,k,[],options);
        sx_fd(:,:,ip) = (solp.x - sol.x)/eps;
        sy_fd(:,:,ip) = (solp.y - sol.y)/eps;
    end
```

PLOTTING

```
    figure
    for ip = 1:4
        subplot(4,2,ip*2-1)
        hold on
        for ix = 1:size(sol.x,2)
            plot(t,sol.sx(:,ix,ip),'.-','Color',c_x(ix,:))
            plot(t,sx_fd(:,ix,options.sens_ind(ip)),'d','Color',c_x(ix,:))
        end
        legend('x1','x1_{fd}','x2','x2_{fd}','x3','x3_{fd}','Location','NorthEastOutside')
        legend boxoff
        title(['state sensitivity for p' num2str(options.sens_ind(ip))])
        xlabel('time t')
        ylabel('x')
        box on

        subplot(4,2,ip*2)
        plot(t,abs(sol.sx(:,:,ip)-sx_fd(:,:,options.sens_ind(ip))),'--')
        legend('error x1','error x2','error x3','Location','NorthEastOutside')
        legend boxoff
        title(['error of state sensitivity for p' num2str(options.sens_ind(ip))])
        xlabel('time t')
        ylabel('error')
        set(gca,'YScale','log')
        box on
    end
    set(gcf,'Position',[100 300 1200 500])
```

STEADY STATE SENSITIVITY

```
    sssens = NaN(size(sol.sx));
    for it = 2:length(t)
        tt = [0,t(it)];
        options.sensi_meth = 'ss';
        solss = simulate_model_steadystate(tt,log10(p),k,[],options);
        sssens(it,:,:) = solss.sx;
        ssxdot(it,:) = solss.xdot;
    end
```

PLOTTING

```
    figure
    for ip = 1:4
        subplot(4,2,ip*2-1)
        hold on
        for ix = 1:size(sol.x,2)
            plot(t,sol.sx(:,ix,ip),'.-','Color',c_x(ix,:))
            plot(t,sssens(:,ix,ip),'d-','Color',c_x(ix,:))
        end
        legend('x1','x1_{ss}','x2','x2_{ss}','x3','x3_{ss}','Location','NorthEastOutside')
        legend boxoff
        title(['state steady sensitivity for p' num2str(ip)])
        xlabel('time t')
        ylabel('x')
        box on

        subplot(4,2,ip*2)
        plot(t,abs(sol.sx(:,:,ip)-sssens(:,:,ip)),'--')
        legend('error x1','error x2','error x3','Location','NorthEastOutside')
        legend boxoff
        title(['error of steady state sensitivity for p' num2str(ip)])
        xlabel('time t')
        ylabel('error')
        set(gca,'YScale','log')
        box on
    end
    set(gcf,'Position',[100 300 1200 500])

    figure
    scatter(sqrt(sum((ssxdot./sol.x).^2,2)),sqrt(sum(sum((sol.sx-sssens).^2,2),3)))
    hold on
    plot([1e-15,1e5],[1e-15,1e5],'k:')
    set(gca,'YScale','log')
    set(gca,'XScale','log')
    box on
    axis square
    xlabel('||dxdt/x||_2')
    ylabel('error steady state approximation')
    set(gca,'FontSize',15)
    set(gca,'LineWidth',1.5)
    set(gcf,'Position',[100 300 1200 500])

    drawnow
```

 

```
end
```

Published with MATLAB® R2016a
