# Supplementary material for: Scalable Parameter Estimation for Genome-Scale Biochemical Reaction Networks
Source: PLoS Comput Biol. 2017 Jan 23;13(1):e1005331. doi: 10.1371/journal.pcbi.1005331 (PMC5256869; doi:10.1371/journal.pcbi.1005331)
Supplement: S1 Code — This zip-file contains the MATLAB code for the simulation and application examples presented in the paper. We provide implementations of all models, parameter estimation to allow everybody to reproduce the results. (ZIP) [file pcbi.1005331.s002.zip › code/AMICI/SuiteSparse/CAMD/Doc/CAMD_UserGuide.pdf]

CAMD User Guide

Patrick R. Amestoy* Yanqing (Morris) Chen Timothy A. Davis† Iain S. Duff‡

VERSION 2.4.1, Oct 10, 2014

Abstract

CAMD is a set of ANSI C routines that implements the approximate minimum degree ordering algorithm to permute sparse matrices prior to numerical factorization. Ordering constraints can be optionally provided. A MATLAB interface is included.

CAMD Copyright©2013 by Timothy A. Davis, Yanqing (Morris) Chen, Patrick R. Amestoy, and Iain S. Duff. All Rights Reserved. CAMD is available under alternate licences; contact T. Davis for details.

CAMD License: Your use or distribution of CAMD or any modified version of CAMD implies that you agree to this License.

This library is free software; you can redistribute it and/or modify it under the terms of the GNU Lesser General Public License as published by the Free Software Foundation; either version 2.1 of the License, or (at your option) any later version.

This library is distributed in the hope that it will be useful, but WITHOUT ANY WARRANTY; without even the implied warranty of MERCHANTABILITY or FITNESS FOR A PARTICULAR PURPOSE. See the GNU Lesser General Public License for more details.

You should have received a copy of the GNU Lesser General Public License along with this library; if not, write to the Free Software Foundation, Inc., 51 Franklin St, Fifth Floor, Boston, MA 02110-1301 USA

Permission is hereby granted to use or copy this program under the terms of the GNU LGPL, provided that the Copyright, this License, and the Availability of the original version is retained on all copies. User documentation of any code that uses this code or any modified version of this code must cite the Copyright, this License, the Availability note, and "Used by permission." Permission to modify the code and to distribute modified code is granted, provided the Copyright, this License, and the Availability note are retained, and a notice that the code was modified is included.

Availability: http://www.suitesparse.com.

Acknowledgments:

This work was supported by the National Science Foundation, under grants ASC-9111263 and DMS-9223088 and CCR-0203270, and by Sandia National Labs (a grant from DOE). The conversion

*ENSEEIHT-IRIT, 2 rue Camichel 31017 Toulouse, France. email: amestoy@enseeiht.fr. http://www.enseeiht.fr/∼amestoy.

†email: DrTimothyAldenDavis@gmail.com, http://www.suitesparse.com. This work was supported by the National Science Foundation, under grants ASC-9111263, DMS-9223088, and CCR-0203270. Portions of the work were done while on sabbatical at Stanford University and Lawrence Berkeley National Laboratory (with funding from Stanford University and the SciDAC program). Ordering constraints added with support from Sandia National Laboratory (Dept. of Energy).

‡Rutherford Appleton Laboratory, Chilton, Didcot, Oxon OX11 0QX, England. email: i.s.duff@rl.ac.uk. http://www.numerical.rl.ac.uk/people/isd/isd.html. This work was supported by the EPSRC under grant GR/R46441.

to C, the addition of the elimination tree post-ordering, and the handling of dense rows and columns were done while Davis was on sabbatical at Stanford University and Lawrence Berkeley National Laboratory. The ordering constraints were added by Chen and Davis.

1 Overview

CAMD is a set of routines for preordering a sparse matrix prior to numerical factorization. It uses an approximate minimum degree ordering algorithm [1, 2] to find a permutation matrix $\mathbf{P}$ so that the Cholesky factorization $\mathbf{PAP}^\mathsf{T} = \mathbf{LL}^\mathsf{T}$ has fewer (often much fewer) nonzero entries than the Cholesky factorization of $\mathbf{A}$. The algorithm is typically much faster than other ordering methods and minimum degree ordering algorithms that compute an exact degree [4]. Some methods, such as approximate deficiency [9] and graph-partitioning based methods [5, 7, 8, 10] can produce better orderings, depending on the matrix.

The algorithm starts with an undirected graph representation of a symmetric sparse matrix $\mathbf{A}$. Node i in the graph corresponds to row and column i of the matrix, and there is an edge (i, j) in the graph if a_{ij} is nonzero. The degree of a node is initialized to the number of off-diagonal nonzeros in row i, which is the size of the set of nodes adjacent to i in the graph.

The selection of a pivot a_{ii} from the diagonal of $\mathbf{A}$ and the first step of Gaussian elimination corresponds to one step of graph elimination. Numerical fill-in causes new nonzero entries in the matrix (fill-in refers to nonzeros in $\mathbf{L}$ that are not in $\mathbf{A}$). Node i is eliminated and edges are added to its neighbors so that they form a clique (or *element*). To reduce fill-in, node i is selected as the node of least degree in the graph. This process repeats until the graph is eliminated.

The clique is represented implicitly. Rather than listing all the new edges in the graph, a single list of nodes is kept which represents the clique. This list corresponds to the nonzero pattern of the first column of $\mathbf{L}$. As the elimination proceeds, some of these cliques become subsets of subsequent cliques, and are removed. This graph can be stored in place, that is using the same amount of memory as the original graph.

The most costly part of the minimum degree algorithm is the recomputation of the degrees of nodes adjacent to the current pivot element. Rather than keep track of the exact degree, the approximate minimum degree algorithm finds an upper bound on the degree that is easier to compute. For nodes of least degree, this bound tends to be tight. Using the approximate degree instead of the exact degree leads to a substantial savings in run time, particularly for very irregularly structured matrices. It has no effect on the quality of the ordering.

The elimination phase is followed by an elimination tree post-ordering. This has no effect on fill-in, but reorganizes the ordering so that the subsequent numerical factorization is more efficient. It also includes a pre-processing phase in which nodes of very high degree are removed (without causing fill-in), and placed last in the permutation $\mathbf{P}$ (subject to the constraints). This reduces the run time substantially if the matrix has a few rows with many nonzero entries, and has little effect on the quality of the ordering. CAMD operates on the symmetric nonzero pattern of $\mathbf{A} + \mathbf{A}^\mathsf{T}$, so it can be given an unsymmetric matrix, or either the lower or upper triangular part of a symmetric matrix.

CAMD has the ability to order the matrix with constraints. Each node i in the graph (row/column i in the matrix) has a constraint, `C[i]`, which is in the range `0` to `n-1`. All nodes with `C[i] = 0` are ordered first, followed by all nodes with constraint `1`, and so on. That is, `C[P[k]]` is monotonically non-decreasing as `k` varies from `0` to `n-1`. If `C` is NULL, no constraints are used (the ordering will be similar to AMD's ordering, except that the postordering is different). The optional `C` parameter is also provided in the MATLAB interface, (`p = camd (A,Control,C)`).

For a discussion of the long history of the minimum degree algorithm, see [4].

2 Availability

CAMD is available at http://www.suitesparse.com. The Fortran version is available as the routine `MC47` in HSL (formerly the Harwell Subroutine Library) [6]. `MC47` does not include ordering constraints.

3 Using CAMD in MATLAB

To use CAMD in MATLAB, you must first compile the CAMD mexFunction. Just type `make` in the Unix system shell, while in the `CAMD` directory. You can also type `camd_make` in MATLAB, while in the `CAMD/MATLAB` directory. Place the `CAMD/MATLAB` directory in your MATLAB path. This can be done on any system with MATLAB, including Windows. See Section 6 for more details on how to install CAMD.

The MATLAB statement `p=camd(A)` finds a permutation vector `p` such that the Cholesky factorization `chol(A(p,p))` is typically sparser than `chol(A)`. If `A` is unsymmetric, `camd(A)` is identical to `camd(A+A')` (ignoring numerical cancellation). If `A` is not symmetric positive definite, but has substantial diagonal entries and a mostly symmetric nonzero pattern, then this ordering is also suitable for LU factorization. A partial pivoting threshold may be required to prevent pivots from being selected off the diagonal, such as the statement `[L,U,P] = lu (A (p,p), 0.1)`. Type `help lu` for more details. The statement `[L,U,P,Q] = lu (A (p,p))` in MATLAB 6.5 is not suitable, however, because it uses UMFPACK Version 4.0 and thus does not attempt to select pivots from the diagonal. UMFPACK Version 4.1 in MATLAB 7.0 and later uses several strategies, including a symmetric pivoting strategy, and will give you better results if you want to factorize an unsymmetric matrix of this type. Refer to the UMFPACK User Guide for more details, at http://www.suitesparse.com.

The CAMD mexFunction is much faster than the built-in MATLAB symmetric minimum degree ordering methods, SYMAMD and SYMMMD. Its ordering quality is essentially identical to AMD, comparable to SYMAMD, and better than SYMMMD [3].

An optional input argument can be used to modify the control parameters for CAMD (aggressive absorption, dense row/column handling, and printing of statistics). An optional output argument provides statistics on the ordering, including an analysis of the fill-in and the floating-point operation count for a subsequent factorization. For more details (once CAMD is installed), type `help camd` in the MATLAB command window.

4 Using CAMD in a C program

The C-callable CAMD library consists of seven user-callable routines and one include file. There are two versions of each of the routines, with `int` and `long` integers. The routines with prefix `camd_l_` use `long` integer arguments; the others use `int` integer arguments. If you compile CAMD in the standard ILP32 mode (32-bit `int`'s, `long`'s, and pointers) then the versions are essentially identical. You will be able to solve problems using up to 2GB of memory. If you compile CAMD in the standard LP64 mode, the size of an `int` remains 32-bits, but the size of a `long` and a pointer both get promoted to 64-bits.

The following routines are fully described in Section 7:

- `camd_order` (`long` version: `camd_l_order`)

```
#include "camd.h"
```

```
int n, Ap [n+1], Ai [nz], P [n], C [n] ;
double Control [CAMD_CONTROL], Info [CAMD_INFO] ;
int result = camd_order (n, Ap, Ai, P, Control, Info, C) ;
```

Computes the approximate minimum degree ordering of an n-by-n matrix $\mathbf{A}$. Returns a permutation vector `P` of size `n`, where `P[k] = i` if row and column `i` are the `k`th row and column in the permuted matrix. This routine allocates its own memory of size $1.2e + 9n$ integers, where e is the number of nonzeros in $\mathbf{A} + \mathbf{A}^\mathsf{T}$. It computes statistics about the matrix $\mathbf{A}$, such as the symmetry of its nonzero pattern, the number of nonzeros in $\mathbf{L}$, and the number of floating-point operations required for Cholesky and LU factorizations (which are returned in the `Info` array). The user's input matrix is not modified. It returns `CAMD_OK` if successful, `CAMD_OK_BUT_JUMBLED` if successful (but the matrix had unsorted and/or duplicate row indices), `CAMD_INVALID` if the matrix is invalid, `CAMD_OUT_OF_MEMORY` if out of memory.

The array `C` provides the ordering constraints. On input, `C` may be null (to denote no constraints); otherwise, it must be an array size `n`, with entries in the range `0` to `n-1`. On output, `C[P[0..n-1]]` is monotonically non-descreasing.

- `camd_defaults` (`long` version: `camd_l_defaults`)

```
#include "camd.h"
double Control [CAMD_CONTROL] ;
camd_defaults (Control) ;
```

Sets the default control parameters in the `Control` array. These can then be modified as desired before passing the array to the other CAMD routines.

- `camd_control` (`long` version: `camd_l_control`)

```
#include "camd.h"
double Control [CAMD_CONTROL] ;
camd_control (Control) ;
```

Prints a description of the control parameters, and their values.

- `camd_info` (`long` version: `camd_l_info`)

```
#include "camd.h"
double Info [CAMD_INFO] ;
camd_info (Info) ;
```

Prints a description of the statistics computed by CAMD, and their values.

- `camd_valid` (`long` version: `camd_valid`)

```
#include "camd.h"
int n, Ap [n+1], Ai [nz] ;
int result = camd_valid (n, n, Ap, Ai) ;
```

Returns `CAMD_OK` or `CAMD_OK_BUT_JUMBLED` if the matrix is valid as input to `camd_order`; the latter is returned if the matrix has unsorted and/or duplicate row indices in one or more columns. Returns `CAMD_INVALID` if the matrix cannot be passed to `camd_order`. For `camd_order`, the matrix must also be square. The first two arguments are the number of rows and the number of columns of the matrix. For its use in CAMD, these must both equal `n`.

- `camd_2` (`long` version: `camd_l2`) CAMD ordering kernel. It is faster than `camd_order`, and can be called by the user, but it is difficult to use. It does not check its inputs for errors. It does not require the columns of its input matrix to be sorted, but it destroys the matrix on output. Additional workspace must be passed. Refer to the source file `CAMD/Source/camd_2.c` for a description.

The nonzero pattern of the matrix $\mathbf{A}$ is represented in compressed column form. For an n-by-n matrix $\mathbf{A}$ with `nz` nonzero entries, the representation consists of two arrays: `Ap` of size `n+1` and `Ai` of size `nz`. The row indices of entries in column `j` are stored in `Ai[Ap[j] ... Ap[j+1]-1]`. For `camd_order`, if duplicate row indices are present, or if the row indices in any given column are not sorted in ascending order, then `camd_order` creates an internal copy of the matrix with sorted rows and no duplicate entries, and orders the copy. This adds slightly to the time and memory usage of `camd_order`, but is not an error condition.

The matrix is 0-based, and thus row indices must be in the range `0` to `n-1`. The first entry `Ap[0]` must be zero. The total number of entries in the matrix is thus `nz = Ap[n]`.

The matrix must be square, but it does not need to be symmetric. The `camd_order` routine constructs the nonzero pattern of $\mathbf{B} = \mathbf{A} + \mathbf{A}^\mathsf{T}$ (without forming $\mathbf{A}^\mathsf{T}$ explicitly if $\mathbf{A}$ has sorted columns and no duplicate entries), and then orders the matrix $\mathbf{B}$. Thus, either the lower triangular part of $\mathbf{A}$, the upper triangular part, or any combination may be passed. The transpose $\mathbf{A}^\mathsf{T}$ may also be passed to `camd_order`. The diagonal entries may be present, but are ignored.

4.1 Control parameters

Control parameters are set in an optional `Control` array. It is optional in the sense that if a `NULL` pointer is passed for the `Control` input argument, then default control parameters are used.

- `Control[CAMD_DENSE]` (or `Control(1)` in MATLAB): controls the threshold for "dense" rows/columns. A dense row/column in $\mathbf{A} + \mathbf{A}^\mathsf{T}$ can cause CAMD to spend significant time in ordering the matrix. If `Control[CAMD_DENSE]` ≥ 0, rows/columns with more than `Control[CAMD_DENSE]` $\sqrt{n}$ entries are ignored during the ordering, and placed last in the output order. The default value of `Control[CAMD_DENSE]` is 10. If negative, no rows/columns are treated as "dense." Rows/columns with 16 or fewer off-diagonal entries are never considered "dense."

- `Control[CAMD_AGGRESSIVE]` (or `Control(2)` in MATLAB): controls whether or not to use aggressive absorption, in which a prior element is absorbed into the current element if it is a subset of the current element, even if it is not adjacent to the current pivot element (refer to [1, 2] for more details). The default value is nonzero, which means that aggressive absorption will be performed. This nearly always leads to a better ordering (because the approximate degrees are more accurate) and a lower execution time. There are cases where it can lead to a slightly worse ordering, however. To turn it off, set `Control[CAMD_AGGRESSIVE]` to 0.

Statistics are returned in the `Info` array (if `Info` is `NULL`, then no statistics are returned). Refer to `camd.h` file, for more details (14 different statistics are returned, so the list is not included here).

4.2 Sample C program

The following program, camd_demo.c, illustrates the basic use of CAMD. See Section 5 for a short description of each calling sequence.

```
#include <stdio.h>
#include "camd.h"

int n = 5 ;
int Ap [ ] = { 0,   2,       6,       10,  12, 14} ;
int Ai [ ] = { 0,1, 0,1,2,4, 1,2,3,4, 2,3, 1,4   } ;
int C [ ] = { 2, 0, 0, 0, 1 } ;
int P [5] ;

int main (void)
{
    int k ;
    (void) camd_order (n, Ap, Ai, P, (double *) NULL, (double *) NULL, C) ;
    for (k = 0 ; k < n ; k++) printf ("P [%d] = %d\n", k, P [k]) ;
    return (0) ;
}
```

The Ap and Ai arrays represent the binary matrix

$$\mathbf{A} = \left[\begin{array}{ccccc} 1 & 1 & 0 & 0 & 0 \\ 1 & 1 & 1 & 0 & 1 \\ 0 & 1 & 1 & 1 & 0 \\ 0 & 0 & 1 & 1 & 0 \\ 0 & 1 & 1 & 0 & 1 \end{array} \right].$$

The diagonal entries are ignored. CAMD constructs the pattern of $\mathbf{A}+\mathbf{A}^{\mathsf{T}}$, and returns a permutation vector of $(3, 2, 1, 4, 0)$. Note that nodes 1, 2, and 3 appear first (they are in the constraint set 0), node 4 appears next (since `C[4] = 1`), and node 0 appears last. Since the matrix is unsymmetric but with a mostly symmetric nonzero pattern, this would be a suitable permutation for an LU factorization of a matrix with this nonzero pattern and whose diagonal entries are not too small. The program uses default control settings and does not return any statistics about the ordering, factorization, or solution (`Control` and `Info` are both `(double *) NULL`). It also ignores the status value returned by camd_order.

More example programs are included with the CAMD package. The camd_demo.c program provides a more detailed demo of CAMD. Another example is the CAMD mexFunction, camd_mex.c.

4.3 A note about zero-sized arrays

CAMD uses several user-provided arrays of size `n` or `nz`. Either `n` or `nz` can be zero. If you attempt to `malloc` an array of size zero, however, `malloc` will return a null pointer which CAMD will report as invalid. If you `malloc` an array of size `n` or `nz` to pass to CAMD, make sure that you handle the `n` = 0 and `nz = 0` cases correctly.

5 Synopsis of C-callable routines

The matrix $\mathbf{A}$ is `n`-by-`n` with `nz` entries.

```
#include "camd.h"
int n, status, Ap [n+1], Ai [nz], P [n], C [n] ;
double Control [CAMD_CONTROL], Info [CAMD_INFO] ;
camd_defaults (Control) ;
status = camd_order (n, Ap, Ai, P, Control, Info, C) ;
camd_control (Control) ;
camd_info (Info) ;
status = camd_valid (n, n, Ap, Ai) ;
```

The `camd_l_*` routines are identical, except that all `int` arguments become `long`:

```
#include "camd.h"
long n, status, Ap [n+1], Ai [nz], P [n], C [n] ;
double Control [CAMD_CONTROL], Info [CAMD_INFO] ;
camd_l_defaults (Control) ;
status = camd_l_order (n, Ap, Ai, P, Control, Info, C) ;
camd_l_control (Control) ;
camd_l_info (Info) ;
status = camd_l_valid (n, n, Ap, Ai) ;
```

6 Installation

The following discussion assumes you have the `make` program, either in Unix, or in Windows with Cygwin.

System-dependent configurations are in the `../SuiteSparse_config/SuiteSparse_config.mk` file. You can edit that file to customize the compilation. The default settings will work on most systems. Sample configuration files are provided for Linux, Sun Solaris, SGI IRIX, IBM AIX, and the DEC/Compaq Alpha.

To compile and install the C-callable CAMD library, go to the `CAMD` directory and type `make`. The library will be placed in `CAMD/Lib/libcamd.a`. Three demo programs of the CAMD ordering routine will be compiled and tested in the `CAMD/Demo` directory. The outputs of these demo programs will then be compared with output files in the distribution.

Typing `make clean` will remove all but the final compiled libraries and demo programs. Typing `make purge` or `make distclean` removes all files not in the original distribution. If you compile CAMD and then later change the `../SuiteSparse_config/SuiteSparse_config.mk` file then you should type `make purge` and then `make` to recompile.

When you compile your program that uses the C-callable CAMD library, you need to add the `CAMD/Lib/libcamd.a` library and you need to tell your compiler to look in the `CAMD/Include` directory for include files. See `CAMD/Demo/Makefile` for an example.

If all you want to use is the CAMD mexFunction in MATLAB, you can skip the use of the `make` command entirely. Simply type `camd_make` in MATLAB while in the `CAMD/MATLAB` directory. This works on any system with MATLAB, including Windows. Alternately, type `make` in the `CAMD/MATLAB` directory.

If you are including CAMD as a subset of a larger library and do not want to link the C standard I/O library, or if you simply do not need to use them, you can safely remove the `camd_control.c` and `camd_info.c` files. Similarly, if you use default parameters (or define your own `Control` array), then you can exclude the `camd_defaults.c` file. Each of these files contains the user-callable routines of the same name. None of these auxiliary routines are directly called by `camd_order`. The `camd_dump.c` file contains debugging routines that are neither used nor compiled unless debugging is enabled. The `camd_internal.h` file must be edited to enable debugging; refer to the instructions in that file. The bare minimum files required to use just `camd_order` are `camd.h` and `camd_internal.h`

in the Include directory, and camd_1.c, camd_2.c, camd_aat.c, camd_global.c, and_order.c, camd_postorder.c, camd_preprocess.c, and camd_valid.c in the Source directory.

7 The CAMD routines

The file `CAMD/Include/camd.h` listed below describes each user-callable routine in CAMD, and gives details on their use.

```
/* ========================================================================= */
/* === CAMD:  approximate minimum degree ordering ========================== */
/* ========================================================================= */

/* ------------------------------------------------------------------------- */
/* CAMD Version 2.4, Copyright (c) 2013 by Timothy A. Davis, Yanqing Chen,   */
/* Patrick R. Amestoy, and Iain S. Duff.  See ../README.txt for License.     */
/* email: DrTimothyAldenDavis@gmail.com                                      */
/* ------------------------------------------------------------------------- */

/* CAMD finds a symmetric ordering P of a matrix A so that the Cholesky
 * factorization of P*A*P' has fewer nonzeros and takes less work than the
 * Cholesky factorization of A.  If A is not symmetric, then it performs its
 * ordering on the matrix A+A'.  Two sets of user-callable routines are
 * provided, one for int integers and the other for SuiteSparse_long integers.
 *
 * The method is based on the approximate minimum degree algorithm, discussed
 * in Amestoy, Davis, and Duff, "An approximate degree ordering algorithm",
 * SIAM Journal of Matrix Analysis and Applications, vol. 17, no. 4, pp.
 * 886-905, 1996.
 */

#ifndef CAMD_H
#define CAMD_H

/* make it easy for C++ programs to include CAMD */
#ifdef __cplusplus
extern "C" {
#endif

/* get the definition of size_t: */
#include <stddef.h>

#include "SuiteSparse_config.h"

int camd_order              /* returns CAMD_OK, CAMD_OK_BUT_JUMBLED,
                             * CAMD_INVALID, or CAMD_OUT_OF_MEMORY */
(
    int n,                  /* A is n-by-n.  n must be >= 0. */
    const int Ap [ ],       /* column pointers for A, of size n+1 */
    const int Ai [ ],       /* row indices of A, of size nz = Ap [n] */
    int P [ ],              /* output permutation, of size n */
    double Control [ ],     /* input Control settings, of size CAMD_CONTROL */
    double Info [ ],        /* output Info statistics, of size CAMD_INFO */
    const int C [ ]         /* Constraint set of A, of size n; can be NULL */
) ;

SuiteSparse_long camd_l_order   /* see above for description of arguments */
(
    SuiteSparse_long n,
    const SuiteSparse_long Ap [ ],
    const SuiteSparse_long Ai [ ],
    SuiteSparse_long P [ ],
    double Control [ ],
```

```
    double Info [ ],
    const SuiteSparse_long C [ ]
) ;

/* Input arguments (not modified):
 *
 *      n: the matrix A is n-by-n.
 *      Ap: an int/SuiteSparse_long array of size n+1, containing column
 *          pointers of A.
 *      Ai: an int/SuiteSparse_long array of size nz, containing the row
 *          indices of A, where nz = Ap [n].
 *      Control:  a double array of size CAMD_CONTROL, containing control
 *          parameters.  Defaults are used if Control is NULL.
 *
 * Output arguments (not defined on input):
 *
 *      P: an int/SuiteSparse_long array of size n, containing the output
 *          permutation. If row i is the kth pivot row, then P [k] = i.  In
 *          MATLAB notation, the reordered matrix is A (P,P).
 *      Info: a double array of size CAMD_INFO, containing statistical
 *          information.  Ignored if Info is NULL.
 *
 * On input, the matrix A is stored in column-oriented form.  The row indices
 * of nonzero entries in column j are stored in Ai [Ap [j] ... Ap [j+1]-1].
 *
 * If the row indices appear in ascending order in each column, and there
 * are no duplicate entries, then camd_order is slightly more efficient in
 * terms of time and memory usage.  If this condition does not hold, a copy
 * of the matrix is created (where these conditions do hold), and the copy is
 * ordered.
 *
 * Row indices must be in the range 0 to
 * n-1.  Ap [0] must be zero, and thus nz = Ap [n] is the number of nonzeros
 * in A.  The array Ap is of size n+1, and the array Ai is of size nz = Ap [n].
 * The matrix does not need to be symmetric, and the diagonal does not need to
 * be present (if diagonal entries are present, they are ignored except for
 * the output statistic Info [CAMD_NZDIAG]).  The arrays Ai and Ap are not
 * modified.  This form of the Ap and Ai arrays to represent the nonzero
 * pattern of the matrix A is the same as that used internally by MATLAB.
 * If you wish to use a more flexible input structure, please see the
 * umfpack_*_triplet_to_col routines in the UMFPACK package, at
 * http://www.suitesparse.com.
 *
 * Restrictions:  n >= 0.  Ap [0] = 0.  Ap [j] <= Ap [j+1] for all j in the
 *      range 0 to n-1.  nz = Ap [n] >= 0.  Ai [0..nz-1] must be in the range 0
 *      to n-1.  Finally, Ai, Ap, and P must not be NULL.  If any of these
 *      restrictions are not met, CAMD returns CAMD_INVALID.
 *
 * CAMD returns:
 *
 *      CAMD_OK if the matrix is valid and sufficient memory can be allocated to
 *          perform the ordering.
 *
 *      CAMD_OUT_OF_MEMORY if not enough memory can be allocated.
 *
 *      CAMD_INVALID if the input arguments n, Ap, Ai are invalid, or if P is
 *          NULL.
 *
 *      CAMD_OK_BUT_JUMBLED if the matrix had unsorted columns, and/or duplicate
```

```
*           entries, but was otherwise valid.
*
* The CAMD routine first forms the pattern of the matrix A+A', and then
* computes a fill-reducing ordering, P.  If P [k] = i, then row/column i of
* the original is the kth pivotal row.  In MATLAB notation, the permuted
* matrix is A (P,P), except that 0-based indexing is used instead of the
* 1-based indexing in MATLAB.
*
* The Control array is used to set various parameters for CAMD.  If a NULL
* pointer is passed, default values are used.  The Control array is not
* modified.
*
*       Control [CAMD_DENSE]:  controls the threshold for "dense" rows/columns.
*           A dense row/column in A+A' can cause CAMD to spend a lot of time in
*           ordering the matrix.  If Control [CAMD_DENSE] >= 0, rows/columns
*           with more than Control [CAMD_DENSE] * sqrt (n) entries are ignored
*           during the ordering, and placed last in the output order.  The
*           default value of Control [CAMD_DENSE] is 10.  If negative, no
*           rows/columns are treated as "dense".  Rows/columns with 16 or
*           fewer off-diagonal entries are never considered "dense".
*
*       Control [CAMD_AGGRESSIVE]: controls whether or not to use aggressive
*           absorption, in which a prior element is absorbed into the current
*           element if is a subset of the current element, even if it is not
*           adjacent to the current pivot element (refer to Amestoy, Davis,
*           & Duff, 1996, for more details).  The default value is nonzero,
*           which means to perform aggressive absorption.  This nearly always
*           leads to a better ordering (because the approximate degrees are
*           more accurate) and a lower execution time.  There are cases where
*           it can lead to a slightly worse ordering, however.  To turn it off,
*           set Control [CAMD_AGGRESSIVE] to 0.
*
*       Control [2..4] are not used in the current version, but may be used in
*           future versions.
*
* The Info array provides statistics about the ordering on output.  If it is
* not present, the statistics are not returned.  This is not an error
* condition.
*
*       Info [CAMD_STATUS]:  the return value of CAMD, either CAMD_OK,
*           CAMD_OK_BUT_JUMBLED, CAMD_OUT_OF_MEMORY, or CAMD_INVALID.
*
*       Info [CAMD_N]: n, the size of the input matrix
*
*       Info [CAMD_NZ]: the number of nonzeros in A, nz = Ap [n]
*
*       Info [CAMD_SYMMETRY]:  the symmetry of the matrix A.  It is the number
*           of "matched" off-diagonal entries divided by the total number of
*           off-diagonal entries.  An entry A(i,j) is matched if A(j,i) is also
*           an entry, for any pair (i,j) for which i != j.  In MATLAB notation,
*               S = spones (A) ;
*               B = tril (S, -1) + triu (S, 1) ;
*               symmetry = nnz (B & B') / nnz (B) ;
*
*       Info [CAMD_NZDIAG]: the number of entries on the diagonal of A.
*
*       Info [CAMD_NZ_A_PLUS_AT]:  the number of nonzeros in A+A', excluding the
*           diagonal.  If A is perfectly symmetric (Info [CAMD_SYMMETRY] = 1)
*           with a fully nonzero diagonal, then Info [CAMD_NZ_A_PLUS_AT] = nz-n
```

```
 *          (the smallest possible value).  If A is perfectly unsymmetric
 *          (Info [CAMD_SYMMETRY] = 0, for an upper triangular matrix, for
 *          example) with no diagonal, then Info [CAMD_NZ_A_PLUS_AT] = 2*nz
 *          (the largest possible value).
 *
 *      Info [CAMD_NDENSE]: the number of "dense" rows/columns of A+A' that were
 *          removed from A prior to ordering.  These are placed last in the
 *          output order P.
 *
 *      Info [CAMD_MEMORY]: the amount of memory used by CAMD, in bytes.  In the
 *          current version, this is 1.2 * Info  [CAMD_NZ_A_PLUS_AT] + 9*n
 *          times the size of an integer.  This is at most 2.4nz + 9n.  This
 *          excludes the size of the input arguments Ai, Ap, and P, which have
 *          a total size of nz + 2*n + 1 integers.
 *
 *      Info [CAMD_NCMPA]: the number of garbage collections performed.
 *
 *      Info [CAMD_LNZ]: the number of nonzeros in L (excluding the diagonal).
 *          This is a slight upper bound because mass elimination is combined
 *          with the approximate degree update.  It is a rough upper bound if
 *          there are many "dense" rows/columns.  The rest of the statistics,
 *          below, are also slight or rough upper bounds, for the same reasons.
 *          The post-ordering of the assembly tree might also not exactly
 *          correspond to a true elimination tree postordering.
 *
 *      Info [CAMD_NDIV]: the number of divide operations for a subsequent LDL'
 *          or LU factorization of the permuted matrix A (P,P).
 *
 *      Info [CAMD_NMULTSUBS_LDL]:  the number of multiply-subtract pairs for a
 *          subsequent LDL' factorization of A (P,P).
 *
 *      Info [CAMD_NMULTSUBS_LU]:  the number of multiply-subtract pairs for a
 *          subsequent LU factorization of A (P,P), assuming that no numerical
 *          pivoting is required.
 *
 *      Info [CAMD_DMAX]:  the maximum number of nonzeros in any column of L,
 *          including the diagonal.
 *
 *      Info [14..19] are not used in the current version, but may be used in
 *          future versions.
 */

/* ------------------------------------------------------------------------- */
/* direct interface to CAMD */
/* ------------------------------------------------------------------------- */

/* camd_2 is the primary CAMD ordering routine.  It is not meant to be
 * user-callable because of its restrictive inputs and because it destroys
 * the user's input matrix.  It does not check its inputs for errors, either.
 * However, if you can work with these restrictions it can be faster than
 * camd_order and use less memory (assuming that you can create your own copy
 * of the matrix for CAMD to destroy).  Refer to CAMD/Source/camd_2.c for a
 * description of each parameter. */

void camd_2
(
    int n,
    int Pe [ ],
    int Iw [ ],
```

```
    int Len [ ],
    int iwlen,
    int pfree,
    int Nv [ ],
    int Next [ ],
    int Last [ ],
    int Head [ ],
    int Elen [ ],
    int Degree [ ],
    int W [ ],
    double Control [ ],
    double Info [ ],
    const int C [ ],
    int BucketSet [ ]
) ;

void camd_l2
(
    SuiteSparse_long n,
    SuiteSparse_long Pe [ ],
    SuiteSparse_long Iw [ ],
    SuiteSparse_long Len [ ],
    SuiteSparse_long iwlen,
    SuiteSparse_long pfree,
    SuiteSparse_long Nv [ ],
    SuiteSparse_long Next [ ],
    SuiteSparse_long Last [ ],
    SuiteSparse_long Head [ ],
    SuiteSparse_long Elen [ ],
    SuiteSparse_long Degree [ ],
    SuiteSparse_long W [ ],
    double Control [ ],
    double Info [ ],
    const SuiteSparse_long C [ ],
    SuiteSparse_long BucketSet [ ]

) ;

/* ------------------------------------------------------------------------- */
/* camd_valid */
/* ------------------------------------------------------------------------- */

/* Returns CAMD_OK or CAMD_OK_BUT_JUMBLED if the matrix is valid as input to
 * camd_order; the latter is returned if the matrix has unsorted and/or
 * duplicate row indices in one or more columns.  Returns CAMD_INVALID if the
 * matrix cannot be passed to camd_order.  For camd_order, the matrix must also
 * be square.  The first two arguments are the number of rows and the number
 * of columns of the matrix.  For its use in CAMD, these must both equal n.
 */

int camd_valid
(
    int n_row,              /* # of rows */
    int n_col,              /* # of columns */
    const int Ap [ ],       /* column pointers, of size n_col+1 */
    const int Ai [ ]        /* row indices, of size Ap [n_col] */
) ;

SuiteSparse_long camd_l_valid
```

```
(
    SuiteSparse_long n_row,
    SuiteSparse_long n_col,
    const SuiteSparse_long Ap [ ],
    const SuiteSparse_long Ai [ ]
) ;

/* ------------------------------------------------------------------------- */
/* camd_cvalid */
/* ------------------------------------------------------------------------- */

/* Returns TRUE if the constraint set is valid as input to camd_order,
 * FALSE otherwise. */

int camd_cvalid
(
   int n,
   const int C [ ]
) ;

SuiteSparse_long camd_l_cvalid
(
   SuiteSparse_long n,
   const SuiteSparse_long C [ ]
) ;

/* ------------------------------------------------------------------------- */
/* CAMD memory manager and printf routines */
/* ------------------------------------------------------------------------- */

    /* moved to SuiteSparse_config.c */

/* ------------------------------------------------------------------------- */
/* CAMD Control and Info arrays */
/* ------------------------------------------------------------------------- */

/* camd_defaults:  sets the default control settings */
void camd_defaults   (double Control [ ]) ;
void camd_l_defaults (double Control [ ]) ;

/* camd_control: prints the control settings */
void camd_control    (double Control [ ]) ;
void camd_l_control  (double Control [ ]) ;

/* camd_info: prints the statistics */
void camd_info       (double Info [ ]) ;
void camd_l_info     (double Info [ ]) ;

#define CAMD_CONTROL 5      /* size of Control array */
#define CAMD_INFO 20        /* size of Info array */

/* contents of Control */
#define CAMD_DENSE 0        /* "dense" if degree > Control [0] * sqrt (n) */
#define CAMD_AGGRESSIVE 1    /* do aggressive absorption if Control [1] != 0 */

/* default Control settings */
#define CAMD_DEFAULT_DENSE 10.0     /* default "dense" degree 10*sqrt(n) */
#define CAMD_DEFAULT_AGGRESSIVE 1    /* do aggressive absorption by default */
```

```
/* contents of Info */
#define CAMD_STATUS 0       /* return value of camd_order and camd_l_order */
#define CAMD_N 1                    /* A is n-by-n */
#define CAMD_NZ 2           /* number of nonzeros in A */
#define CAMD_SYMMETRY 3     /* symmetry of pattern (1 is sym., 0 is unsym.) */
#define CAMD_NZDIAG 4       /* # of entries on diagonal */
#define CAMD_NZ_A_PLUS_AT 5  /* nz in A+A' */
#define CAMD_NDENSE 6       /* number of "dense" rows/columns in A */
#define CAMD_MEMORY 7       /* amount of memory used by CAMD */
#define CAMD_NCMPA 8        /* number of garbage collections in CAMD */
#define CAMD_LNZ 9          /* approx. nz in L, excluding the diagonal */
#define CAMD_NDIV 10        /* number of fl. point divides for LU and LDL' */
#define CAMD_NMULTSUBS_LDL 11 /* number of fl. point (*,-) pairs for LDL' */
#define CAMD_NMULTSUBS_LU 12  /* number of fl. point (*,-) pairs for LU */
#define CAMD_DMAX 13         /* max nz. in any column of L, incl. diagonal */

/* ------------------------------------------------------------------------- */
/* return values of CAMD */
/* ------------------------------------------------------------------------- */

#define CAMD_OK 0               /* success */
#define CAMD_OUT_OF_MEMORY -1   /* malloc failed, or problem too large */
#define CAMD_INVALID -2         /* input arguments are not valid */
#define CAMD_OK_BUT_JUMBLED 1   /* input matrix is OK for camd_order, but
    * columns were not sorted, and/or duplicate entries were present.  CAMD had
    * to do extra work before ordering the matrix.  This is a warning, not an
    * error.  */

/* ========================================================================== */
/* === CAMD version ========================================================= */
/* ========================================================================== */

/*
 * As an example, to test if the version you are using is 1.2 or later:
 *
 *      if (CAMD_VERSION >= CAMD_VERSION_CODE (1,2)) ...
 *
 * This also works during compile-time:
 *
 *      #if (CAMD_VERSION >= CAMD_VERSION_CODE (1,2))
 *          printf ("This is version 1.2 or later\n") ;
 *      #else
 *          printf ("This is an early version\n") ;
 *      #endif
 */

#define CAMD_DATE "Oct 10, 2014"
#define CAMD_VERSION_CODE(main,sub) ((main) * 1000 + (sub))
#define CAMD_MAIN_VERSION 2
#define CAMD_SUB_VERSION 4
#define CAMD_SUBSUB_VERSION 1
#define CAMD_VERSION CAMD_VERSION_CODE(CAMD_MAIN_VERSION,CAMD_SUB_VERSION)

#ifdef __cplusplus
}
#endif

#endif
```

References

[1] P. R. Amestoy, T. A. Davis, and I. S. Duff. An approximate minimum degree ordering algorithm. *SIAM J. Matrix Anal. Applic.*, 17(4):886–905, 1996.

[2] P. R. Amestoy, T. A. Davis, and I. S. Duff. Algorithm 837: An approximate minimum degree ordering algorithm. *ACM Trans. Math. Softw.*, 30(3):381–388, 2004.

[3] T. A. Davis, J. R. Gilbert, S. I. Larimore, and E. G. Ng. A column approximate minimum degree ordering algorithm. *ACM Trans. Math. Softw.*, 30:353–376, 2004.

[4] A. George and J. W. H. Liu. The evolution of the minimum degree ordering algorithm. *SIAM Review*, 31(1):1–19, 1989.

[5] B. Hendrickson and E. Rothberg. Improving the runtime and quality of nested dissection ordering. *SIAM J. Sci. Comput.*, 20:468–489, 1999.

[6] HSL. HSL 2002: A collection of Fortran codes for large scale scientific computation, 2002. `www.cse.clrc.ac.uk/nag/hsl`.

[7] G. Karypis and V. Kumar. A fast and high quality multilevel scheme for partitioning irregular graphs. *SIAM J. Sci. Comput.*, 20:359–392, 1998.

[8] F. Pellegrini, J. Roman, and P. Amestoy. Hybridizing nested dissection and halo approximate minimum degree for efficient sparse matrix ordering. *Concurrency: Practice and Experience*, 12:68–84, 2000.

[9] E. Rothberg and S. C. Eisenstat. Node selection strategies for bottom-up sparse matrix orderings. *SIAM J. Matrix Anal. Applic.*, 19(3):682–695, 1998.

[10] J. Schulze. Towards a tighter coupling of bottom-up and top-down sparse matrix ordering methods. *BIT*, 41(4):800–841, 2001.
